# Network Pharmacology Reveals That Resveratrol Can Alleviate COVID-19-Related Hyperinflammation

**DOI:** 10.1155/2021/4129993

**Published:** 2021-09-22

**Authors:** Zijian Xiao, Qing Ye, Xiaomei Duan, Tao Xiang

**Affiliations:** The First Affiliated Hospital, Department of Neurology, Hengyang Medical School, University of South China, Hengyang, Hunan, China

## Abstract

Hyperinflammation is related to the development of COVID-19. Resveratrol is considered an anti-inflammatory and antiviral agent. Herein, we used a network pharmacological approach and bioinformatic gene analysis to explore the pharmacological mechanism of Resveratrol in COVID-19 therapy. Potential targets of Resveratrol were obtained from public databases. SARS-CoV-2 differentially expressed genes (DEGs) were screened out via bioinformatic analysis Gene Expression Omnibus (GEO) datasets GSE147507, followed by Gene Ontology (GO) and Kyoto Encyclopedia of Genes and Genomes (KEGG) pathway enrichment analysis; then, protein-protein interaction network was constructed. The common targets, GO terms, and KEGG pathways of Resveratrol targets and SARS-CoV-2 DEGs were confirmed. KEGG Mapper queried the location of common targets in the key pathways. A notable overlap of the GO terms and KEGG pathways between Resveratrol targets and SARS-CoV-2 DEGs was revealed. The shared targets between Resveratrol targets and SARS-CoV-2 mainly involved the IL-17 signaling pathway, NF-kappa B signaling pathway, and TNF signaling pathway. Our study uncovered that Resveratrol is a promising therapeutic candidate for COVID-19 and we also revealed the probable key targets and pathways involved. Ultimately, we bring forward new insights and encourage more studies on Resveratol to benefit COVID-19 patients.

## 1. Introduction

The outbreak of coronavirus disease 2019 (COVID-19) has caused a global health emergency. People worldwide are still being challenged by the enhanced infection risk as coronavirus 2 (SARS-CoV-2) is rapidly spreading [1]. Hence, researchers urgently need to accelerate clinical trials of any possible effective and tolerable drug that may reduce the mortality rate in severe SARS-CoV-2 pneumonia patients.

Severe COVID-19 patients often demonstrate acute respiratory distress syndrome (ARDS). Proinflammatory cytokines in the blood were found to be upregulated in COVID-19 patients, including interleukin- (IL-) 1, *IL-6*, tumor necrosis factor (*TNF*), and interferon *γ*. Studies suggest that a subgroup of patients with severe COVID-19 might have acquired cytokine storm syndrome [1]. Accumulated evidence has confirmed that excessive inflammation, oxidation, and an exaggerated immune response may play an important role during a cytokine storm and subsequent progression to acute lung injury (ALI)/ARDS and often death [1, 2]. Approved therapies to alleviate hyperinflammation and improve the prognosis of severe COVID-19 patients are recommended.

Resveratrol (PubChem CID: 445154) is a phytoalexin that can be extracted from grapes and a wide range of plants and possesses antioxidant and potential chemopreventive properties. Resveratrol displays anti-inflammatory effects via regulating immune cells and interfering with the synthesis of proinflammatory cytokines [3–5]. Resveratrol also acts as an antiviral agent through different mechanisms of action. Resveratrol has been shown to inhibit various viruses, including respiratory syncytial virus, influenza virus, human metapneumonia virus, Epstein–Barr virus, enterovirus, and HIV [6–8].

Our previous study predicted that astragaloside IV could alleviate hyperinflammation in COVID-19 using network pharmacology methodology [9]. Resveratrol possesses not only antiviral properties but also anti-inflammatory effects; however, there is still no research focused on Resveratrol in the treatment of COVID-19 at the moment. In this study, a network pharmacological approach and bioinformatic gene analysis strategy were adopted to investigate the mechanism of action underlying the effectiveness of Resveratrol in COVID-19 therapy.

## 2. Materials and Methods

### 2.1. Potential Resveratrol-Related Targets

The word “Resveratrol” was searched in PubChem (https://pubchem.ncbi.nlm.nih.gov/), which is the world's largest collection of freely accessible chemical information [10]; molecular structure and PubChem CID (445154) of Resveratrol were obtained. Comparative Toxicogenomics Database (CTD, http://ctdbase.org/about/), a robust publicly available database that provides manually curated information about chemical–gene/protein interactions and chemical–disease and gene-disease relationships [11], and TargetNet (http://targetnet.scbdd.com), an open web server that is used for netting or predicting the binding of multiple targets for any given molecule [12], were used to predict potential targets for Resveratrol. To improve accuracy, we took the target genes found in the intersecting region of these two databases. Because of the nonstandard naming, the names of targets were listed using the official symbol format from the UniProt Knowledgebase (UniProtKB, http://www.uniprot.org/).

### 2.2. SARS-CoV-2-Related Genes

The GSE147507 dataset containing the host transcriptional response to SARS-CoV-2 was downloaded from GEO [13]. During the publisher's study, SARS-CoV-2 (USA-WA1/2020) was used to stimulate primary human lung epithelium (NHBE) and transformed lung alveolar (A549) cells, which suggested that the unique transcriptional signature of this virus may be responsible for the development of COVID-19 [14]. We chose the transcriptional results of NHBE for analysis. R packages of “impute” and “limma” provided by the Bioconductor project (http://www.bioconductor.org/packages/release/bioc/html/affy.html) [15] were applied to assess the transcriptional results of NHBE. Quantile normalization and log2-transformation were used to create a robust multiarray average (RMA). Adjusted original *p* values were obtained via the Benjamini-Hochberg method; the false discovery rate (FDR) procedure was used to calculate fold changes (FC). Gene expression values of ∣log2 FC | >1 and *p* value < 0.05 were used as a threshold to filter differentially expressed genes (DEGs).

### 2.3. PPI Network Construction

Intersecting target genes of Resveratrol and DEGs related to SARS-CoV-2 were obtained. The Resveratrol-related targets and SARS-CoV-2 DEGs were uploaded to String (https://string-db.org/) [16], with species set as “Homo sapiens,” a confidence score > 0.9 to construct PPI networks, and then, the 2 PPI networks were merged and visualized using Cytoscape 3.7.2 (http://www.cytoscape.org/) [17].

### 2.4. KEGG Pathway and Gene Ontology (GO) Enrichment Analysis

A list of Resveratrol targets and DEGs related to SARS-CoV-2 were submitted to Metascape (http://metascape.org) [18], with species limited to “Homo sapiens.” KEGG pathway, GO Biological Processes, GO Cellular Components, and GO Molecular Functions analysis were carried out with the following ontology sources: the enrichment background are all genes in the genome. *p* value < 0.01, count > 3, and a minimum enrichment factor of 1.5 were used as filtering terms. Common KEGG pathways and GO terms between Resveratrol-related targets and SARS-CoV-2's DEGs were chosen. We listed the overlapping targets and related key pathways. KEGG Mapper queried the location of SARS-CoV-2 DEGs and shared targets in the key pathways.

### 2.5. In Silico Molecular Docking Study of Resveratrol Key Targets

Molecular docking of Resveratrol with the common targets between Resveratrol and SARS-CoV-2 was performed using Autodock Vina [19]. The molecular structure of Resveratrol was downloaded in the PDB format from the PubChem database (https://www.ncbi.nlm.nih.gov/) [20]. The molecular structures of the targets were obtained from the Protein Data Bank (http://www.rcsb.org/) [21]. Before docking, the original crystal ligands and water molecules were removed from the protein-ligand complexes. Hydrogen atoms and charge were added, and default settings were selected for other parameters. Local Search Parameters were selected as the molecular docking model of Resveratrol to the protein targets. The docking score was used to evaluate the theoretical binding affinities of Resveratrol to the common targets.

## 3. Results

### 3.1. Potential Targets of Resveratrol

**Figure 1:**
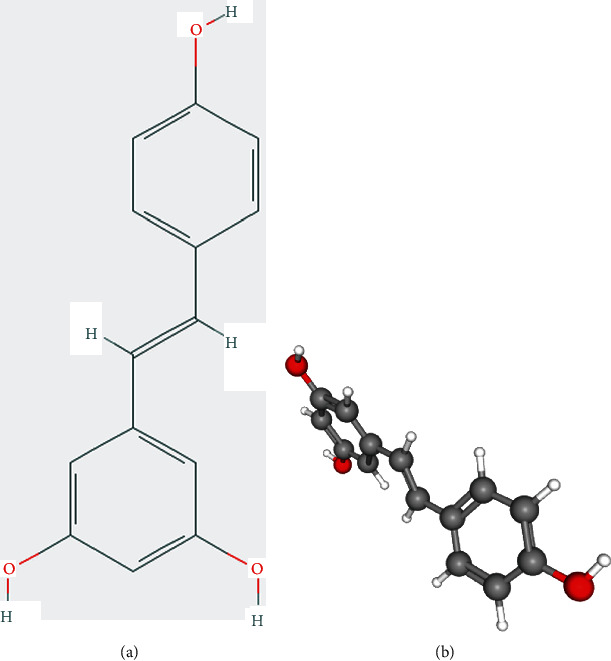
Molecular structure of Resveratrol: (a) 2D molecular structure; (b) 3D molecular structure.

**Figure 2:**
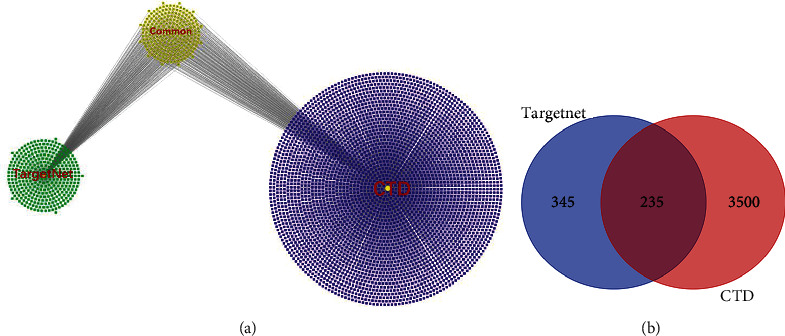
Resveratrol-related targets: (a) the green circle on the left represents targets from TargetNet, the blue circle on the right represents targets from CTD, and the yellow circle in the middle indicates the overlapping targets of TargetNet and CTD. (b) Targets from TargetNet and CTD are shown in the Venn diagram.

The molecular structure of Resveratrol was downloaded from the PubChem database (Figure 1). Then, 616 corresponding potential targets of Resveratrol were extracted from TargetNet, while 3735 corresponding potential targets of Resveratrol were obtained from the Comparative Toxicogenomics Database (CTD). After comparing common targets, 235 potential targets were selected, as shown in Figure 2 and Supplemental file Table S1.

### 3.2. Identification of SARS-CoV-2 DEGs

**Figure 3:**
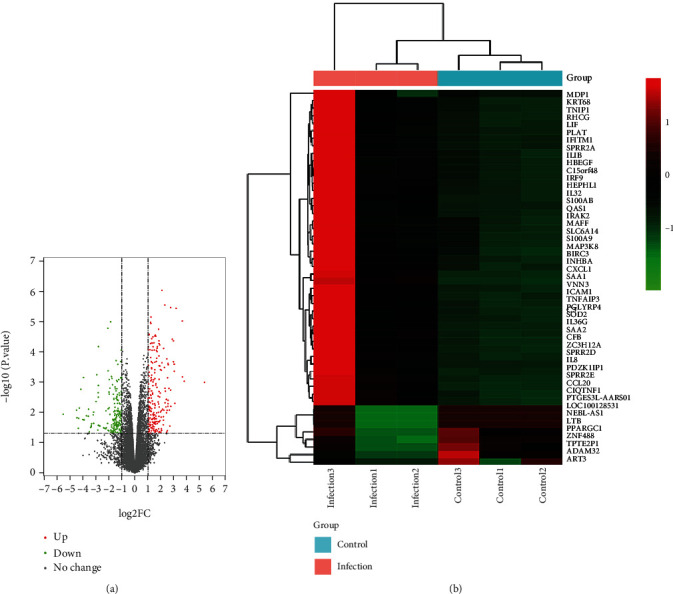
(a) Volcano plots of the significantly expressed (*p* < 0.05, fold change > 1) mRNAs between control and SARS-CoV-2 samples after analysis of the GSE147507 dataset. Red dots represent upregulated genes; green dots represent downregulated genes. (b) Heatmaps depicting the expression levels of the top 50 DEGs ranked by *p* value among SARS-CoV-2 DEGs. Legend on the top right indicates log fold change of genes (Infection1, Infection2, Infection3 = infected with SARS-CoV-2 samples; Control1, Control2, Control3 = mock-treated samples).

In total, we identified 23710 genes when comparing SARS-CoV-2 and control samples and 510 of them were considered significantly differentially expressed, including 270 downregulated genes and 240 upregulated genes. Heatmaps were used to display the expression levels of the top 50 DEGs ranked by *p* value (Figure 3 and Supplemental file Table S2).

### 3.3. PPI Network Analysis

**Figure 4:**
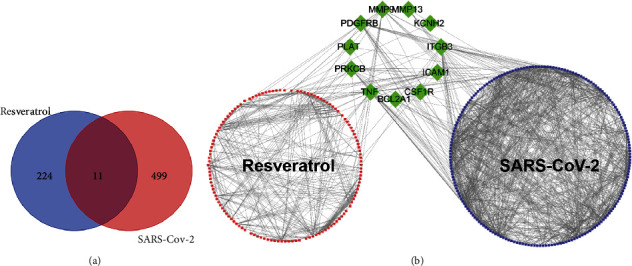
PPI network analysis. (a) Targets of Resveratrol and SARS-CoV-2 DEGs are shown in the Venn diagram. There are 235 Resveratrol-related targets and 510 SARS-CoV-2 DEGs, and both share 11 targets. (b) The red circle on the left represents Resveratrol-related targets, the blue circle on the right represents SARS-CoV-2 DEGs, and the green rhombus in the middle indicates the overlapping targets between Resveratrol and SARS-CoV-2 DEGs; edges represent correlations between targets.

Overlapping targets of Resveratrol and SARS-CoV-2 DEGs included the following: *MMP13*, *PRKCB*, *PLAT*, *KCNH2*, *ICAM1*, *PDGFRB*, *TNF*, *ITGB3*, *CSF1R*, *BCL2A1*, and *MMP9*. PPI network was used to visualize and quantify the function of specific proteins in cells at the systematic level [22]. PPI network of Resveratrol-related targets and SARS-CoV-2 DEGs were constructed, and the common targets were identified (Figure 4).

### 3.4. Gene Ontology and KEGG Enrichment Analysis of Resveratrol-Related Targets and SARS-CoV-2 DEGs

**Figure 5:**
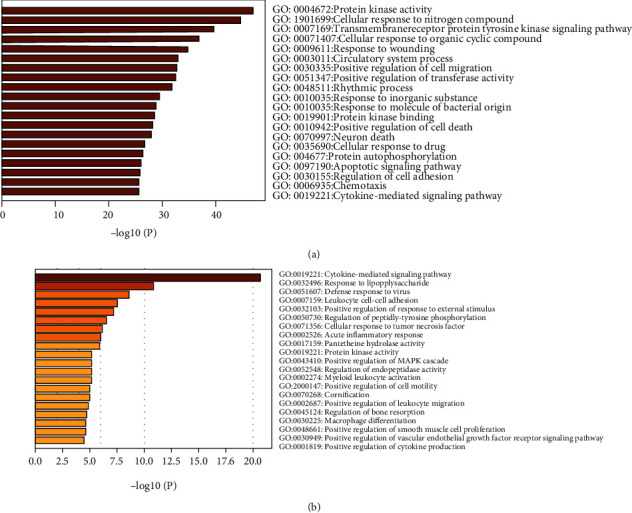
GO enrichment analysis. (a) The most significant items in Resveratrol-related targets are classified according to the *p* value. (b) The most significant items in SARS-CoV-2 DEGs are ranked by *p* value.

GO analysis identified 266 enriched terms for Resveratrol-related targets, including 240 Biological Process terms, 23 Molecular Function terms, and 3 Cellular Component terms. The most significant items ranked by *p* value are listed in Figure 5(a) and Supplemental file Table S3. SARS-CoV-2 DEGs displayed 315 enriched GO terms, including 299 Biological Process terms, 13 Molecular Function terms, and 3 Cellular Component terms. The most significant items were ranked using *p* value and are listed in Figure 5(b) and Supplemental file Table S4. The intersecting region between Resveratrol-related targets and SARS-CoV-2 DEGs contained 89 terms; the top 10 common items were ranked by *p* value and are listed in Table 1.

**Figure 6:**
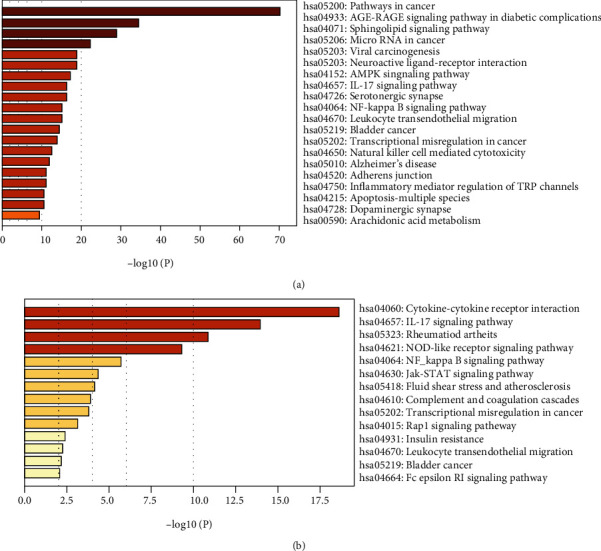
KEGG enrichment analysis. (a) The most significant enriched KEGG pathways are classified according to their *p* value in Resveratrol-related targets. (b) The most significant enriched KEGG pathways are ranked by *p* value in SARS-CoV-2 DEGs.

**Figure 7:**
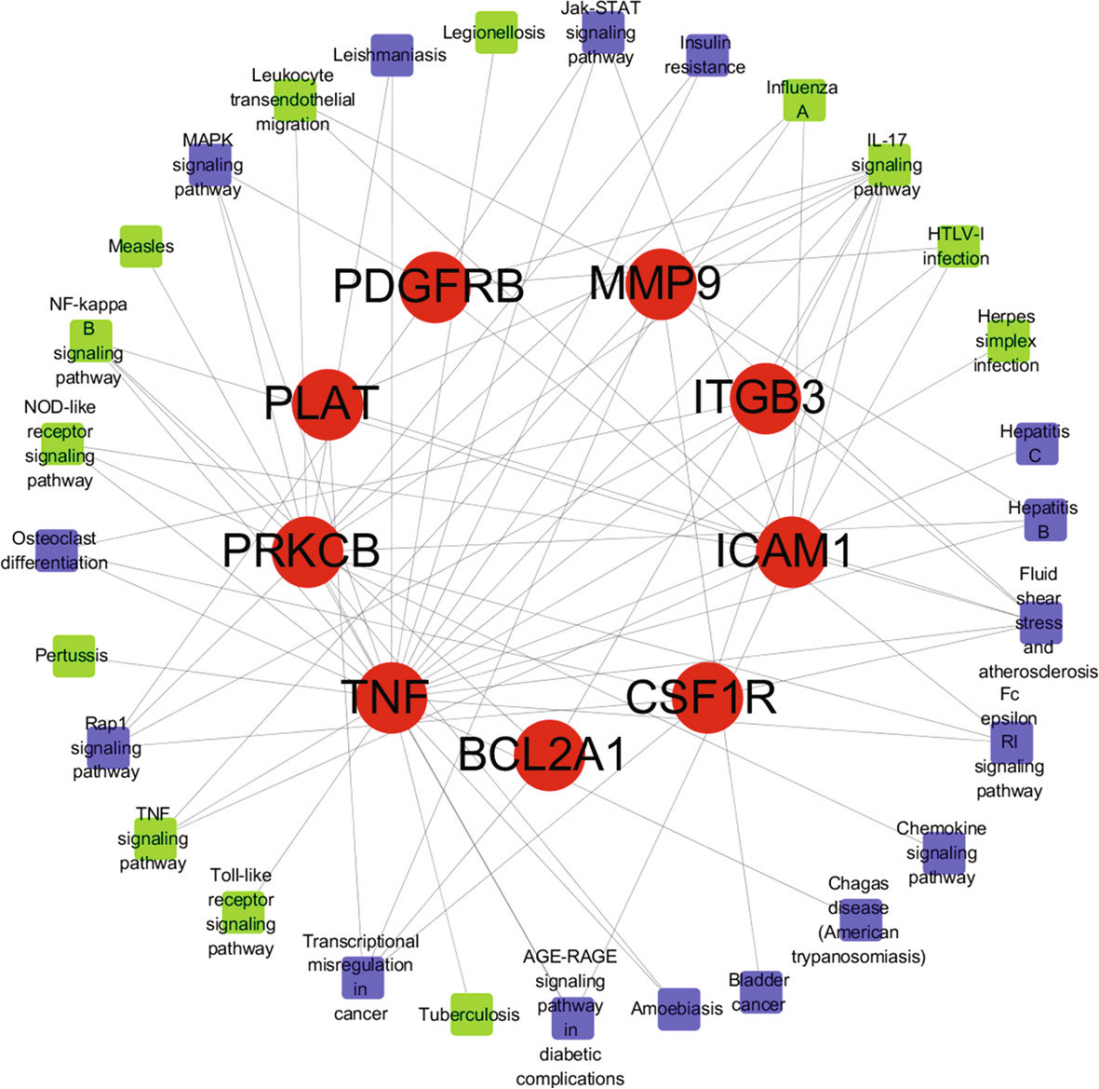
The shared targets have overlapping KEGG pathways. The red circle in the middle represents overlapping Resveratrol-related targets and SARS-CoV-2 DEGs; the green square represents overlapping KEGG pathways in relation to inflammatory and immune response; the blue square represents other items among overlapping KEGG pathways.

**Figure 8:**
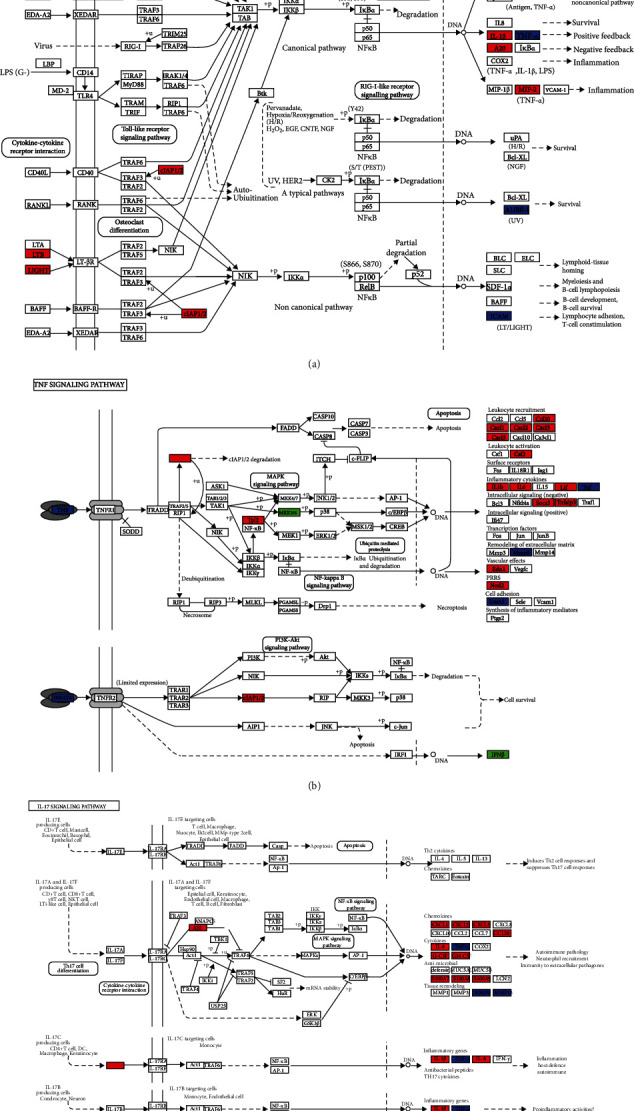
The key KEGG pathways: (a) NF-*κ*B signaling pathway, (b) TNF signaling pathway, and (c) IL-17 signaling pathway. The red nodes represent upregulated SARS-CoV-2 DEGs, the green marked node represents downexpression SARS-CoV-2 DEGs, and the blue marked node represents overlapping targets between Resveratrol and SARS-CoV-2.

For Resveratrol-related targets, KEGG analysis revealed 142 enriched pathways and the most significant pathways were listed by *p* value strength in Figure 6(a) and Supplemental file Table S5. For SARS-CoV-2 DEGs, KEGG analysis showed 40 enriched pathways, ranked by *p* value in Figure 6(b) and Supplemental file Table S6. The intersection of Resveratrol-related targets and SARS-CoV-2 DEGs included 30 terms, the top 10 items in the intersection ordered by *p* value are listed in Table 2. The common targets and overlapping KEGG pathways are listed in Figure 7. The key KEGG pathways and the location of SARS-CoV-2 DEGs and overlapping genes of enriched pathways are listed in Figure 8.

### 3.5. Molecular Docking Analysis

**Figure 9:**
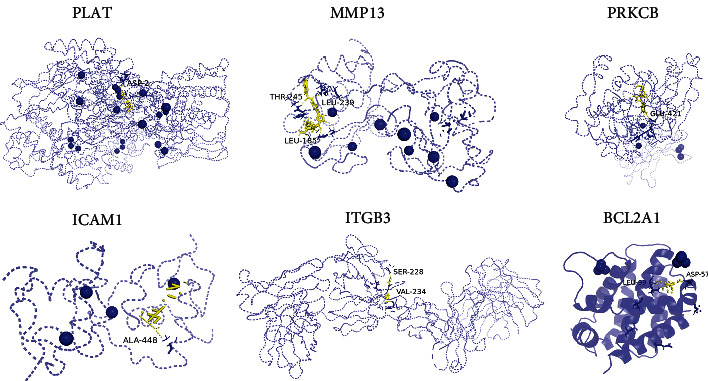
Structural interactions of Resveratrol and key target receptors.

Molecular docking analysis showed that the docking scores of Resveratrol in relation to PLAT, MMP13, PRKCB, ICAM1, and ITGB3 are greater than 5 kcal/mol. Interestingly, Resveratrol displayed the highest docking score with PLAT and MMP13 (docking score: -6.93, -6.27), demonstrating that Resveratrol could form a strong interaction with PLAT and MMP13. Other key targets that showed an affinity with Resveratrol are further listed in Figure 9 and Table 3.

## 4. Discussion

We screened 235 potential Resveratrol-related targets from an online database; GO analysis of these targets revealed enriched terms such as cytokine-mediated signaling pathway, cell chemotaxis, leukocyte chemotaxis, response to lipopolysaccharide, chemotaxis, myeloid leukocyte migration, and cellular response to lipopolysaccharide, which are in line with the findings of a recent study on Resveratrol [23]. 510 DEGs were then confirmed in samples infected with SARS-CoV-2 when compared to mock samples. Similar to the study by Blanco-Melo et al. [24], enriched GO terms such as cellular response to virus infection (GO:0009615), humoral immune response (GO:0006959), and chemokines and cytokines (GO:0005125) were identified.

Among the SARS-CoV-2 DEGs identified, cytokines such as IL-6, IL-8, IL-17, TNF, IL-32, IL-1, and NOD2 were upregulated. These results are in accordance with a recent study that found elevated levels of cytokines in the plasma of COVID-19 patients [1]. GO analysis results of SARS-CoV-2 DEGs reveal that cytokine-mediated signaling pathway, cytokine activity, and neutrophil chemotaxis are the most significant terms. The most relevant KEGG enrichment items were cytokine-cytokine receptor interaction, IL-17 signaling pathway, TNF signaling pathway, NOD-like receptor signaling pathway, and NF-*κ*B signaling pathway. Recent research shows that the NF-*κ*B pathway can be induced by SARS-CoV-2 infection, leading to multiple inflammatory responses [25]. In addition, TNF was present in the blood and disease tissues of patients with COVID-19 [26], which is important in nearly all acute inflammatory reactions, acting as an amplifier of inflammation. Furthermore, *NF-κB*, *IL-6*, and *TNF* are considered promising therapeutic targets in COVID-19 [27].

The shared targets of Resveratrol and SARS-CoV-2 DEGs may represent the potential therapeutic targets of Resveratrol on COVID-19, which include *MMP13*, *PRKCB*, *PLAT*, *KCNH2*, *ICAM1*, *PDGFRB*, *TNF*, *ITGB3*, *CSF1R*, *BCL2A1*, and *MMP9*. These are mainly involved in the IL-17 signaling pathway, NF-*κ*B signaling pathway, and TNF signaling pathway. The activation of these pathways leads to the increased release of cytokines, which have been shown to play an important role in viral infection [28, 29]. We surmise that Resveratrol can reduce the expression level of cytokines and alleviate hyperinflammation in COV19 by inhibiting the activation of these pathways.

Studies have demonstrated that Resveratrol exerts anti-inflammatory effects through various pathways to reduce lung injury [30]. For instance, Resveratrol suppresses TNF-induced activation of nuclear transcription factors NF-*κ*B [31] and also mitigates LPS-induced acute lung inflammation by inhibiting the TLR4/NF-*κ*Bp65/MAPK signaling cascade and *NLRP3* inflammasome [32–34]. Furthermore, Resveratrol improved hyperoxia-induced lung injury via its antioxidant, anti-inflammatory, and antifibrotic effects, promoting the transdifferentiation of alveolar type II epithelial cells into their type I counterpart and suppressing the Wnt/*β*-catenin signaling in preterm rats [35, 36].

By decreasing nucleocapsid (N) protein expression, Resveratrol controlled MERS-CoV infection and improved cellular survival after virus infection [37]. The interaction of nucleocapsid protein and viral RNA in the cytoplasm is necessary for viral RNA nucleocapsid assembly. Resveratrol inhibited the replication of influenza A by nearly 90% by preventing nucleocapsid protein translocation from the nucleus to the cytoplasm [38, 39]. Resveratrol not only reduced the titer of the respiratory syncytial virus but also decreased interferon-*γ* production induced by the respiratory syncytial virus in a mouse model and alleviated airway inflammation and hyperresponsiveness [23, 40]. In human 9HTEo cells, respiratory syncytial virus replication and production of IL-6 were reduced after Resveratrol incubation. Resveratrol treatment also decreased expression of ICAM-1 induced by human rhinoviruses in H1HeLa and nasal epithelial cells [41, 42]. These data demonstrate that Resveratrol might act as a therapeutic drug for viral infections showing both effective anti-inflammatory and antiviral potential. Although there are no experiments to confirm the therapeutic effect of Resveratrol in COVID-19, the molecular docking previously revealed a wide spectrum of interactions between Resveratrol derivatives and two newly released coordinate structures for COVID-19 [43]. The drug for immunosuppression is likely to be beneficial to patients with hyperinflammation. IL-1 blockade (anakinra) showed a significant survival benefit in sepsis patients with hyperinflammation, without increased adverse events [44].

However, our study has some limitations. We failed to prove the therapeutic effect of Resveratrol in COVID-19 through experiments due to complexity reasons. Direct evidence of Resveratrol efficacy is still needed in a SARS-CoV-2 infection experiment model. Nevertheless, we strongly believe that Resveratrol is likely to be beneficial for COVID-19 patients because combined anti-inflammatory and antiviral effects are shown in numerous studies. In light of our findings, related and more in-depth studies on Resveratrol are urgently warranted.

## 5. Conclusion

Taken together, our findings show that Resveratrol is a potential candidate in COVID-19 therapy, based on network pharmacology and bioinformatic gene analysis. More importantly, we also identified the possible key targets and pathways involved in this novel therapeutic strategy.

## Figures and Tables

**Table 1:** Top 10 overlapping GO terms between Resveratrol-related targets and SARS-CoV-2 DEGs ranked by *p* value.

Term	Description	*p* value
GO:0019221	Cytokine-mediated signaling pathway	2.05074*E* − 21
GO:0060326	Cell chemotaxis	5.87962*E* − 12
GO:0030595	Leukocyte chemotaxis	7.40744*E* − 12
GO:0032496	Response to lipopolysaccharide	1.40404*E* − 11
GO:0006935	Chemotaxis	1.68576*E* − 11
GO:0097529	Myeloid leukocyte migration	1.7267*E* − 11
GO:0071222	Cellular response to lipopolysaccharide	1.7267*E* − 11
GO:0042330	Taxis	1.85514*E* − 11
GO:0002237	Response to molecule of bacterial origin	5.64373*E* − 11
GO:0071219	Cellular response to molecule of bacterial origin	5.83252*E* − 11

**Table 2:** Top 10 overlapping KEGG pathways between Resveratrol-related targets and SARS-CoV-2 DEGs ranked by *p* value.

Term	Description	*p* value
hsa04657	IL-17 signaling pathway	9.59366*E* − 15
hsa04668	TNF signaling pathway	1.69723*E* − 13
hsa04621	NOD-like receptor signaling pathway	6.07599*E* − 10
hsa05162	Measles	5.30173*E* − 09
hsa05134	Legionellosis	5.45103*E* − 09
hsa05164	Influenza A	2.00359*E* − 07
hsa05133	Pertussis	1.63392*E* − 06
hsa04064	NF-kappa B signaling pathway	1.8066*E* − 06
hsa05140	Leishmaniasis	9.37339*E* − 06
hsa05146	Amoebiasis	1.38036*E* − 05

**Table 3:** The docking scores of Resveratrol and rapamycin with key proteins.

Target	PDB ID	Drug	Binding energy (kcal/mol)
PLAT	1OLP		-6.93
MMP13	2OW9		-6.27
PRKCB	3PFQ	Resveratrol	-6.17
ICAM1	5E6D		-5.88
ITGB3	4YNY		-5.2
BCL2A1	5UUP		-4.98

## Data Availability

The data used in this study were obtained from open public databases, and data acquisition is explained in the manuscript. SARS-CoV-2 differentially expressed genes (DEGs) were taken from Gene Expression Omnibus (GEO) datasets GSE147507. The molecular structure of Resveratrol was downloaded from the PubChem database (https://pubchem.ncbi.nlm.nih.gov/). TargetNet (http://targetnet.scbdd.com/) was used to predict potential targets for Resveratrol. The molecular structures of the targets were obtained from the Protein Data Bank (http://www.rcsb.org/).

## Abbreviations

COVID-19: Coronavirus disease 2019
CTD: Comparative Toxicogenomics Database
DEGs: Differentially expressed genes
GEO: Gene Expression Omnibus
GO: Gene Ontology
PPI: Protein-protein interaction
NF-*κ*B: NF-kappa B
ARDS: Severe acute respiratory distress syndrome
NHBE: Primary human lung epithelium
A549: Transformed lung alveolar.

## Supplementary Materials

Supplementary 1 Supplementary Table S1: resveratrol-related targets.

Supplementary 2 Supplementary Table S2: SARS-CoV-2 DEGs.

Supplementary 3 Supplementary Table S3: resveratrol-related targets GO analysis-enriched terms.

Supplementary 4 Supplementary Table S4: SARS-CoV-2 DEGs GO analysis-enriched terms.

Supplementary 5 Supplementary Table S5: resveratrol-related targets KEGG-enriched terms.

Supplementary 6 Supplementary Table S6: SARS-CoV-2 DEGs KEGG-enriched terms.
