# Supplementary material for: Network Pharmacology Reveals That Resveratrol Can Alleviate COVID-19-Related Hyperinflammation
Source: Dis Markers. 2021 Sep 22;2021:4129993. doi: 10.1155/2021/4129993 (PMC8463930; doi:10.1155/2021/4129993)
Supplement: Supplementary 1 — Supplementary Table S1: resveratrol-related targets. [file 4129993.f1.pdf]

Resveratrol related targets

Database	Targets	Overlapping targets	Gene ID
Comparative Toxicogenomics Database	AACS	MMP2	4313
Comparative Toxicogenomics Database	AATF	XDH	7498
Comparative Toxicogenomics Database	ABCA1	CXCR4	7852
Comparative Toxicogenomics Database	ABCA6	HSD17B2	3294
Comparative Toxicogenomics Database	ABCB1	MMP7	4316
Comparative Toxicogenomics Database	ABCB1B	PRKCG	5582
Comparative Toxicogenomics Database	ABCB9	NOS2	4843
Comparative Toxicogenomics Database	ABCC1	FGFR1	2260
Comparative Toxicogenomics Database	ABCC2	MET	4233
Comparative Toxicogenomics Database	ABCC3	MTNR1B	4544
Comparative Toxicogenomics Database	ABCC4	CASP6	839
Comparative Toxicogenomics Database	ABCC5	CDC25B	994
Comparative Toxicogenomics Database	ABCC8	CYP2D6	1565
Comparative Toxicogenomics Database	ABCC9	PRKCB	5579
Comparative Toxicogenomics Database	ABCD2	CYP2C19	1557
Comparative Toxicogenomics Database	ABCD4	SMO	6608
Comparative Toxicogenomics Database	ABCF1	ROCK2	9475
Comparative Toxicogenomics Database	ABCF2	IMPDH2	3615
Comparative Toxicogenomics Database	ABCG1	MAOA	4128
Comparative Toxicogenomics Database	ABCG2	GRB2	2885
Comparative Toxicogenomics Database	ABHD11	CDK1	983
Comparative Toxicogenomics Database	ABHD15	PTK2	5747
Comparative Toxicogenomics Database	ABHD6	BCHE	590
Comparative Toxicogenomics Database	ABI1	BCL2	596
Comparative Toxicogenomics Database	ABITRAM	PIK3CB	5291
Comparative Toxicogenomics Database	ABL1	GCK	2645
Comparative Toxicogenomics Database	ABLIM1	THRB	7068
Comparative Toxicogenomics Database	ABTB2	BACE2	25825
Comparative Toxicogenomics Database	ABU-11	OXTR	5021
Comparative Toxicogenomics Database	ABU-15	PTGES	9536
Comparative Toxicogenomics Database	ABU-2	CHRM1	1128
Comparative Toxicogenomics Database	ABU-3	AURKA	6790
Comparative Toxicogenomics Database	ABU-5	SRD5A2	6716
Comparative Toxicogenomics Database	ABU-6	PTGER1	5731
Comparative Toxicogenomics Database	ABU-7	AHR	196
Comparative Toxicogenomics Database	ABU-8	DHFR	1719
Comparative Toxicogenomics Database	ACAA1A	HDAC3	8841
Comparative Toxicogenomics Database	ACAA2	BTK	695
Comparative Toxicogenomics Database	ACACA	ECE1	1889
Comparative Toxicogenomics Database	ACACB	HCK	3055
Comparative Toxicogenomics Database	ACADL	CYP19A1	1588
Comparative Toxicogenomics Database	ACADM	PDGFRB	5159
Comparative Toxicogenomics Database	ACADS	ADRB1	153
Comparative Toxicogenomics Database	ACADSB	TNF	7124
Comparative Toxicogenomics Database	ACAN	SIRT2	22933
Comparative Toxicogenomics Database	ACAT1	FYN	2534
Comparative Toxicogenomics Database	ACE	ITGB1	3688
Comparative Toxicogenomics Database	ACER2	TUBA1A	7846
Comparative Toxicogenomics Database	ACHE	DPP7	29952
Comparative Toxicogenomics Database	ACIN1	JAK2	3717
Comparative Toxicogenomics Database	ACLY	CASP1	834
Comparative Toxicogenomics Database	ACMSD	F2	2147
Comparative Toxicogenomics Database	ACNAT2	XIAP	331
Comparative Toxicogenomics Database	ACOT13	FDFT1	2222
Comparative Toxicogenomics Database	ACOT2	PTGS2	5743
Comparative Toxicogenomics Database	ACOT4	CYP2C9	1559

Comparative Toxicogenomics Database	ACOT7	CTSK	1513
Comparative Toxicogenomics Database	ACOT9	NPY1R	4886
Comparative Toxicogenomics Database	ACOX1	ESR1	2099
Comparative Toxicogenomics Database	ACP1	CDK4	1019
Comparative Toxicogenomics Database	ACP5	TYMS	7298
Comparative Toxicogenomics Database	ACP6	PIM3	415116
Comparative Toxicogenomics Database	ACSL3	PDE4D	5144
Comparative Toxicogenomics Database	ACSL4	MME	4311
Comparative Toxicogenomics Database	ACSL5	PSEN2	5664
Comparative Toxicogenomics Database	ACSM2A	GSK3B	2932
Comparative Toxicogenomics Database	ACSM3	MMP1	4312
Comparative Toxicogenomics Database	ACTA1	PTGER4	5734
Comparative Toxicogenomics Database	ACTA2	WEE1	7465
Comparative Toxicogenomics Database	ACTB	NR2F2	7026
Comparative Toxicogenomics Database	ACTN1	BCL2A1	597
Comparative Toxicogenomics Database	ACTN2	CCR4	1233
Comparative Toxicogenomics Database	ACTN4	PPARD	5467
Comparative Toxicogenomics Database	ACTR3	HMGCR	3156
Comparative Toxicogenomics Database	ACYP1	FLT1	2321
Comparative Toxicogenomics Database	ADAM10	MMP3	4314
Comparative Toxicogenomics Database	ADAM17	DUSP3	1845
Comparative Toxicogenomics Database	ADAM9	CA4	762
Comparative Toxicogenomics Database	ADAMTS4	MAPK1	5594
Comparative Toxicogenomics Database	ADCY4	ALOX5AP	241
Comparative Toxicogenomics Database	ADCY9	PLAT	5327
Comparative Toxicogenomics Database	ADD3	MDM2	4193
Comparative Toxicogenomics Database	ADGB	AKT2	208
Comparative Toxicogenomics Database	ADGRD1	EGFR	1956
Comparative Toxicogenomics Database	ADGRE5	IKBKB	3551
Comparative Toxicogenomics Database	ADGRF2	CXCR3	2833
Comparative Toxicogenomics Database	ADH1B	ERBB2	2064
Comparative Toxicogenomics Database	ADI1	PDE5A	8654
Comparative Toxicogenomics Database	ADIG	MAPK8	5599
Comparative Toxicogenomics Database	ADIPOQ	F2R	2149
Comparative Toxicogenomics Database	ADIPOR1	ADORA3	140
Comparative Toxicogenomics Database	ADIPOR2	ABL1	25
Comparative Toxicogenomics Database	ADM	CCNE2	9134
Comparative Toxicogenomics Database	ADORA1	AKR1B1	231
Comparative Toxicogenomics Database	ADORA2A	HSP90AB1	3326
Comparative Toxicogenomics Database	ADORA3	CHEK2	11200
Comparative Toxicogenomics Database	ADRB1	TERT	7015
Comparative Toxicogenomics Database	AFF4	METAP2	10988
Comparative Toxicogenomics Database	AFTPH	SRC	6714
Comparative Toxicogenomics Database	AGER	CDK2	1017
Comparative Toxicogenomics Database	AGPAT4	PGR	5241
Comparative Toxicogenomics Database	AGPS	THRA	7067
Comparative Toxicogenomics Database	AGR2	CYP17A1	1586
Comparative Toxicogenomics Database	AGR3	CASP9	842
Comparative Toxicogenomics Database	AGT	PRKCQ	5588
Comparative Toxicogenomics Database	AGTR1	ADAM17	6868
Comparative Toxicogenomics Database	AGTR1A	ACACB	32
Comparative Toxicogenomics Database	AGTRAP	PDGFRA	5156
Comparative Toxicogenomics Database	AHCTF1	PRKACA	5566
Comparative Toxicogenomics Database	AHCY	HDAC1	3065
Comparative Toxicogenomics Database	AHNAK	SIRT1	23411
Comparative Toxicogenomics Database	AHR	CHRM3	1131
Comparative Toxicogenomics Database	AHRR	P2RX7	5027
Comparative Toxicogenomics Database	AHSA2	ESR2	2100

Comparative Toxicogenomics Database	AHSG	BRAF	673
Comparative Toxicogenomics Database	AIF1	AKT3	10000
Comparative Toxicogenomics Database	AIF1L	ITGAL	3683
Comparative Toxicogenomics Database	AIFM1	MLYCD	23417
Comparative Toxicogenomics Database	AIMP2	ALPL	249
Comparative Toxicogenomics Database	AK2	PNP	4860
Comparative Toxicogenomics Database	AK6	S1PR3	1903
Comparative Toxicogenomics Database	AKAP8	CCNA1	8900
Comparative Toxicogenomics Database	AKAP9	PLAU	5328
Comparative Toxicogenomics Database	AKR1B1	RAC1	5879
Comparative Toxicogenomics Database	AKR1B10	MMP13	4322
Comparative Toxicogenomics Database	AKR1B3	ALOX5	240
Comparative Toxicogenomics Database	AKR1C3	RXRA	6256
Comparative Toxicogenomics Database	AKT1	NOS1	4842
Comparative Toxicogenomics Database	AKT1S1	NR1H3	10062
Comparative Toxicogenomics Database	AKT2	ACACA	31
Comparative Toxicogenomics Database	AKT3	PLK1	5347
Comparative Toxicogenomics Database	AKTS1	PLA2G2A	5320
Comparative Toxicogenomics Database	ALB	KIF11	3832
Comparative Toxicogenomics Database	ALDH18A1	BDKRB2	624
Comparative Toxicogenomics Database	ALDH1A1	ADORA2A	135
Comparative Toxicogenomics Database	ALDH1A2	KCNH2	3757
Comparative Toxicogenomics Database	ALDH1A3	LIPE	3991
Comparative Toxicogenomics Database	ALDH1B1	CASP8	841
Comparative Toxicogenomics Database	ALDH1L2	PPARG	5468
Comparative Toxicogenomics Database	ALDH2	ALOX15	246
Comparative Toxicogenomics Database	ALDH3A2	PRKCD	5580
Comparative Toxicogenomics Database	ALDH3B2	RET	5979
Comparative Toxicogenomics Database	ALDH4A1	MAPK9	5601
Comparative Toxicogenomics Database	ALDH9A1	SELE	6401
Comparative Toxicogenomics Database	ALDOA	ABCB1	5243
Comparative Toxicogenomics Database	ALDOC	PIM1	5292
Comparative Toxicogenomics Database	ALG14	NR3C1	2908
Comparative Toxicogenomics Database	ALG8	MTOR	2475
Comparative Toxicogenomics Database	ALG9	MAPK14	1432
Comparative Toxicogenomics Database	ALKBH2	CTSS	1520
Comparative Toxicogenomics Database	ALOX12	RAF1	5894
Comparative Toxicogenomics Database	ALOX15	CCR2	729230
Comparative Toxicogenomics Database	ALOX5	ITGAV	3685
Comparative Toxicogenomics Database	ALOX5AP	SCD	6319
Comparative Toxicogenomics Database	ALPI	EGLN1	54583
Comparative Toxicogenomics Database	ALPL	GRIN2B	2904
Comparative Toxicogenomics Database	ALYREF	PDPK1	5170
Comparative Toxicogenomics Database	AMD1	TNK2	10188
Comparative Toxicogenomics Database	AMFR	ADAM10	102
Comparative Toxicogenomics Database	AMH	TGFBR1	7046
Comparative Toxicogenomics Database	AMMECR1	P2RY12	64805
Comparative Toxicogenomics Database	AMOTL1	CDC42	998
Comparative Toxicogenomics Database	ANAPC1	AGTR1	185
Comparative Toxicogenomics Database	ANAPC16	MC1R	4157
Comparative Toxicogenomics Database	ANGPTL4	CASP7	840
Comparative Toxicogenomics Database	ANKRD1	CCKBR	887
Comparative Toxicogenomics Database	ANKRD18A	CTSD	1509
Comparative Toxicogenomics Database	ANLN	ACHE	43
Comparative Toxicogenomics Database	ANO1	SIGMAR1	10280
Comparative Toxicogenomics Database	ANP32E	MTNR1A	4543
Comparative Toxicogenomics Database	ANXA1	MCL1	4170
Comparative Toxicogenomics Database	ANXA11	EPHX2	2053

Comparative Toxicogenomics Database	ANXA2	CDC7	8317
Comparative Toxicogenomics Database	ANXA3	CASP3	836
Comparative Toxicogenomics Database	ANXA4	HSP90AA1	3320
Comparative Toxicogenomics Database	ANXA5	PARP1	142
Comparative Toxicogenomics Database	AOX1	CLK1	1195
Comparative Toxicogenomics Database	AOX3	ACE	1636
Comparative Toxicogenomics Database	AP2A1	ROCK1	6093
Comparative Toxicogenomics Database	AP2A2	KDR	3791
Comparative Toxicogenomics Database	AP2B1	ABCG2	9429
Comparative Toxicogenomics Database	AP3D1	RARA	5914
Comparative Toxicogenomics Database	AP3M1	PTPN1	5770
Comparative Toxicogenomics Database	APAF1	PDE2A	5138
Comparative Toxicogenomics Database	APBB1	PPARA	5465
Comparative Toxicogenomics Database	APCS	MGLL	11343
Comparative Toxicogenomics Database	APEX1	CCR1	1230
Comparative Toxicogenomics Database	API5	ITGA2B	3674
Comparative Toxicogenomics Database	APLN	MAOB	4129
Comparative Toxicogenomics Database	APOA1	CYP1A2	1544
Comparative Toxicogenomics Database	APOA4	PYGL	5836
Comparative Toxicogenomics Database	APOB	DNMT1	1786
Comparative Toxicogenomics Database	APOBEC3B	PIK3CG	5294
Comparative Toxicogenomics Database	APOC1	PIK3CA	5290
Comparative Toxicogenomics Database	APOE	ADORA1	134
Comparative Toxicogenomics Database	APOM	SYK	6850
Comparative Toxicogenomics Database	APOV1	CHRNA4	1137
Comparative Toxicogenomics Database	APP	CCKAR	886
Comparative Toxicogenomics Database	APPBP2	IGF1R	3480
Comparative Toxicogenomics Database	APRT	ICAM1	3383
Comparative Toxicogenomics Database	AQP1	BCL2L1	598
Comparative Toxicogenomics Database	AQP3	SLC9A1	6548
Comparative Toxicogenomics Database	AR	HDAC2	3066
Comparative Toxicogenomics Database	ARAF	CA2	760
Comparative Toxicogenomics Database	ARC	HNF4A	3172
Comparative Toxicogenomics Database	AREG	CDK5R1	8851
Comparative Toxicogenomics Database	ARF1	RELA	5970
Comparative Toxicogenomics Database	ARF4	NOS3	4846
Comparative Toxicogenomics Database	ARF5	KCNA5	3741
Comparative Toxicogenomics Database	ARFGEF1	CHRM2	1129
Comparative Toxicogenomics Database	ARFGEF3	NR1H4	9971
Comparative Toxicogenomics Database	ARG1	SERPINE1	5054
Comparative Toxicogenomics Database	ARG2	VCAM1	7412
Comparative Toxicogenomics Database	ARHGAP5	DYRK1A	1859
Comparative Toxicogenomics Database	ARHGAP9	CDK5	1020
Comparative Toxicogenomics Database	ARHGDIA	JAK1	3716
Comparative Toxicogenomics Database	ARHGEF2	AKT1	207
Comparative Toxicogenomics Database	ARID1B	CCR5	1234
Comparative Toxicogenomics Database	ARID4A	SLC5A1	6523
Comparative Toxicogenomics Database	ARL1	MAP2K1	5604
Comparative Toxicogenomics Database	ARL2	ITGB3	3690
Comparative Toxicogenomics Database	ARL3	HSD11B1	3290
Comparative Toxicogenomics Database	ARL6	GSK3A	2931
Comparative Toxicogenomics Database	ARL8B	AKR1C3	8644
Comparative Toxicogenomics Database	ARNT	PTGS1	5742
Comparative Toxicogenomics Database	ARPC1B	F3	2152
Comparative Toxicogenomics Database	ARPC4	CSF1R	1436
Comparative Toxicogenomics Database	ARPC5	DHODH	1723
Comparative Toxicogenomics Database	ARPC5L	AR	367
Comparative Toxicogenomics Database	ARPP21	FDPS	2224

Comparative Toxicogenomics Database	ARRB2	CYP3A4	1576
Comparative Toxicogenomics Database	ARSG	APP	351
Comparative Toxicogenomics Database	ASAH1	RPS6KB1	6198
Comparative Toxicogenomics Database	ASAP1	MMP9	4318
Comparative Toxicogenomics Database	ASCL1		
Comparative Toxicogenomics Database	ASF1B		
Comparative Toxicogenomics Database	ASH1L		
Comparative Toxicogenomics Database	ASH2L		
Comparative Toxicogenomics Database	ASNS		
Comparative Toxicogenomics Database	ASPH		
Comparative Toxicogenomics Database	ASPM		
Comparative Toxicogenomics Database	ASS1		
Comparative Toxicogenomics Database	ATAD2		
Comparative Toxicogenomics Database	ATE1		
Comparative Toxicogenomics Database	ATF1		
Comparative Toxicogenomics Database	ATF2		
Comparative Toxicogenomics Database	ATF3		
Comparative Toxicogenomics Database	ATF4		
Comparative Toxicogenomics Database	ATF6		
Comparative Toxicogenomics Database	ATG101		
Comparative Toxicogenomics Database	ATG12		
Comparative Toxicogenomics Database	ATG3		
Comparative Toxicogenomics Database	ATG5		
Comparative Toxicogenomics Database	ATL2		
Comparative Toxicogenomics Database	ATL3		
Comparative Toxicogenomics Database	ATM		
Comparative Toxicogenomics Database	ATOX1		
Comparative Toxicogenomics Database	ATP13A1		
Comparative Toxicogenomics Database	ATP1A1		
Comparative Toxicogenomics Database	ATP1B1		
Comparative Toxicogenomics Database	ATP1B3		
Comparative Toxicogenomics Database	ATP2A2		
Comparative Toxicogenomics Database	ATP2A3		
Comparative Toxicogenomics Database	ATP5A1		
Comparative Toxicogenomics Database	ATP5C1		
Comparative Toxicogenomics Database	ATP5E		
Comparative Toxicogenomics Database	ATP5F1A		
Comparative Toxicogenomics Database	ATP5F1B		
Comparative Toxicogenomics Database	ATP5F1C		
Comparative Toxicogenomics Database	ATP5G1		
Comparative Toxicogenomics Database	ATP5G2		
Comparative Toxicogenomics Database	ATP5G3		
Comparative Toxicogenomics Database	ATP5K		
Comparative Toxicogenomics Database	ATP5L		
Comparative Toxicogenomics Database	ATP5MC1		
Comparative Toxicogenomics Database	ATP5O		
Comparative Toxicogenomics Database	ATP5PB		
Comparative Toxicogenomics Database	ATP6V1F		
Comparative Toxicogenomics Database	ATP6V1G2		
Comparative Toxicogenomics Database	ATP8B2		
Comparative Toxicogenomics Database	ATP9B		
Comparative Toxicogenomics Database	ATPAF2		
Comparative Toxicogenomics Database	ATPIF1		
Comparative Toxicogenomics Database	ATR		
Comparative Toxicogenomics Database	ATRX		
Comparative Toxicogenomics Database	ATXN3		
Comparative Toxicogenomics Database	AUH		
Comparative Toxicogenomics Database	AURKA		

Comparative Toxicogenomics Database	AURKB
Comparative Toxicogenomics Database	AUTS2
Comparative Toxicogenomics Database	AXIN2
Comparative Toxicogenomics Database	B2M
Comparative Toxicogenomics Database	B3GALNT1
Comparative Toxicogenomics Database	B3GNT5
Comparative Toxicogenomics Database	BACE2
Comparative Toxicogenomics Database	BAD
Comparative Toxicogenomics Database	BAG2
Comparative Toxicogenomics Database	BAG3
Comparative Toxicogenomics Database	BAG6
Comparative Toxicogenomics Database	BAK1
Comparative Toxicogenomics Database	BAP1
Comparative Toxicogenomics Database	BARD1
Comparative Toxicogenomics Database	BAX
Comparative Toxicogenomics Database	BBC3
Comparative Toxicogenomics Database	BBOX1
Comparative Toxicogenomics Database	BBS1
Comparative Toxicogenomics Database	BBS2
Comparative Toxicogenomics Database	BCAP31
Comparative Toxicogenomics Database	BCAR3
Comparative Toxicogenomics Database	BCAS2
Comparative Toxicogenomics Database	BCAS3
Comparative Toxicogenomics Database	BCAT2
Comparative Toxicogenomics Database	BCHE
Comparative Toxicogenomics Database	BCL10
Comparative Toxicogenomics Database	BCL2
Comparative Toxicogenomics Database	BCL2A1
Comparative Toxicogenomics Database	BCL2L1
Comparative Toxicogenomics Database	BCL2L11
Comparative Toxicogenomics Database	BCL2L12
Comparative Toxicogenomics Database	BCL2L15
Comparative Toxicogenomics Database	BCL2L2
Comparative Toxicogenomics Database	BCL3
Comparative Toxicogenomics Database	BCL6
Comparative Toxicogenomics Database	BCOR
Comparative Toxicogenomics Database	BCR
Comparative Toxicogenomics Database	BCS1L
Comparative Toxicogenomics Database	BDH1
Comparative Toxicogenomics Database	BDKRB2
Comparative Toxicogenomics Database	BDNF
Comparative Toxicogenomics Database	BECN1
Comparative Toxicogenomics Database	BET1
Comparative Toxicogenomics Database	BEX2
Comparative Toxicogenomics Database	BEX3
Comparative Toxicogenomics Database	BEX4
Comparative Toxicogenomics Database	BGLAP
Comparative Toxicogenomics Database	BHLHE40
Comparative Toxicogenomics Database	BHMT
Comparative Toxicogenomics Database	BID
Comparative Toxicogenomics Database	BIK
Comparative Toxicogenomics Database	BIRC2
Comparative Toxicogenomics Database	BIRC3
Comparative Toxicogenomics Database	BIRC5
Comparative Toxicogenomics Database	BIRC7
Comparative Toxicogenomics Database	BLM
Comparative Toxicogenomics Database	BLNK
Comparative Toxicogenomics Database	BLVRA

Comparative Toxicogenomics Database	BMERB1
Comparative Toxicogenomics Database	BMF
Comparative Toxicogenomics Database	BMP2
Comparative Toxicogenomics Database	BMP2K
Comparative Toxicogenomics Database	BMP4
Comparative Toxicogenomics Database	BMP5
Comparative Toxicogenomics Database	BMP6
Comparative Toxicogenomics Database	BMP7
Comparative Toxicogenomics Database	BMPR1B
Comparative Toxicogenomics Database	BMPR2
Comparative Toxicogenomics Database	BNIP1
Comparative Toxicogenomics Database	BNIP2
Comparative Toxicogenomics Database	BOK
Comparative Toxicogenomics Database	BORA
Comparative Toxicogenomics Database	BRAF
Comparative Toxicogenomics Database	BRAP
Comparative Toxicogenomics Database	BRCA1
Comparative Toxicogenomics Database	BRCA2
Comparative Toxicogenomics Database	BRCC3
Comparative Toxicogenomics Database	BRD7
Comparative Toxicogenomics Database	BRF1
Comparative Toxicogenomics Database	BRI3BP
Comparative Toxicogenomics Database	BSCL2
Comparative Toxicogenomics Database	BSG
Comparative Toxicogenomics Database	BST2
Comparative Toxicogenomics Database	BTF3
Comparative Toxicogenomics Database	BTF3L4
Comparative Toxicogenomics Database	BTG1
Comparative Toxicogenomics Database	BTG2
Comparative Toxicogenomics Database	BTK
Comparative Toxicogenomics Database	BUB1
Comparative Toxicogenomics Database	BUB1B
Comparative Toxicogenomics Database	BUB3
Comparative Toxicogenomics Database	BUD31
Comparative Toxicogenomics Database	BYSL
Comparative Toxicogenomics Database	BZW1
Comparative Toxicogenomics Database	C11ORF91
Comparative Toxicogenomics Database	C15ORF48
Comparative Toxicogenomics Database	C1ORF112
Comparative Toxicogenomics Database	C1ORF115
Comparative Toxicogenomics Database	C1ORF116
Comparative Toxicogenomics Database	C1QA
Comparative Toxicogenomics Database	C1QB
Comparative Toxicogenomics Database	C1QC
Comparative Toxicogenomics Database	C1QL2
Comparative Toxicogenomics Database	C1S
Comparative Toxicogenomics Database	C2ORF69
Comparative Toxicogenomics Database	C3
Comparative Toxicogenomics Database	C3ORF52
Comparative Toxicogenomics Database	C4B
Comparative Toxicogenomics Database	C4BPB
Comparative Toxicogenomics Database	C4ORF46
Comparative Toxicogenomics Database	C5
Comparative Toxicogenomics Database	C77080
Comparative Toxicogenomics Database	C9ORF40
Comparative Toxicogenomics Database	CA2
Comparative Toxicogenomics Database	CA4
Comparative Toxicogenomics Database	CACNA1C

Comparative Toxicogenomics Database	CACNA2D1
Comparative Toxicogenomics Database	CACYBP
Comparative Toxicogenomics Database	CAD
Comparative Toxicogenomics Database	CALCA
Comparative Toxicogenomics Database	CALCR
Comparative Toxicogenomics Database	CALD1
Comparative Toxicogenomics Database	CALR
Comparative Toxicogenomics Database	CALU
Comparative Toxicogenomics Database	CAMK2A
Comparative Toxicogenomics Database	CAMK2N1
Comparative Toxicogenomics Database	CAMK4
Comparative Toxicogenomics Database	CAMP
Comparative Toxicogenomics Database	CANX
Comparative Toxicogenomics Database	CAP2
Comparative Toxicogenomics Database	CAPG
Comparative Toxicogenomics Database	CAPN9
Comparative Toxicogenomics Database	CAPNS1
Comparative Toxicogenomics Database	CAPRIN1
Comparative Toxicogenomics Database	CAPSL
Comparative Toxicogenomics Database	CAPZA2
Comparative Toxicogenomics Database	CAPZB
Comparative Toxicogenomics Database	CAR3
Comparative Toxicogenomics Database	CARD16
Comparative Toxicogenomics Database	CARHSP1
Comparative Toxicogenomics Database	CASP1
Comparative Toxicogenomics Database	CASP10
Comparative Toxicogenomics Database	CASP12
Comparative Toxicogenomics Database	CASP2
Comparative Toxicogenomics Database	CASP3
Comparative Toxicogenomics Database	CASP4
Comparative Toxicogenomics Database	CASP6
Comparative Toxicogenomics Database	CASP7
Comparative Toxicogenomics Database	CASP8
Comparative Toxicogenomics Database	CASP8AP2
Comparative Toxicogenomics Database	CASP9
Comparative Toxicogenomics Database	CAT
Comparative Toxicogenomics Database	CAV1
Comparative Toxicogenomics Database	CAV3
Comparative Toxicogenomics Database	CAVIN2
Comparative Toxicogenomics Database	CBFB
Comparative Toxicogenomics Database	CBL
Comparative Toxicogenomics Database	CBR1
Comparative Toxicogenomics Database	CBR2
Comparative Toxicogenomics Database	CBR3
Comparative Toxicogenomics Database	CBX1
Comparative Toxicogenomics Database	CBX5
Comparative Toxicogenomics Database	CCAR2
Comparative Toxicogenomics Database	CCDC113
Comparative Toxicogenomics Database	CCDC117
Comparative Toxicogenomics Database	CCDC12
Comparative Toxicogenomics Database	CCDC137
Comparative Toxicogenomics Database	CCDC14
Comparative Toxicogenomics Database	CCDC80
Comparative Toxicogenomics Database	CCDC88A
Comparative Toxicogenomics Database	CCDC88B
Comparative Toxicogenomics Database	CCK
Comparative Toxicogenomics Database	CCKAR
Comparative Toxicogenomics Database	CCKBR

Comparative Toxicogenomics Database	CCL1
Comparative Toxicogenomics Database	CCL11
Comparative Toxicogenomics Database	CCL12
Comparative Toxicogenomics Database	CCL2
Comparative Toxicogenomics Database	CCL20
Comparative Toxicogenomics Database	CCL21
Comparative Toxicogenomics Database	CCL22
Comparative Toxicogenomics Database	CCL26
Comparative Toxicogenomics Database	CCL3
Comparative Toxicogenomics Database	CCL4
Comparative Toxicogenomics Database	CCL5
Comparative Toxicogenomics Database	CCL6
Comparative Toxicogenomics Database	CCL7
Comparative Toxicogenomics Database	CCL8
Comparative Toxicogenomics Database	CCN2
Comparative Toxicogenomics Database	CCN4
Comparative Toxicogenomics Database	CCN5
Comparative Toxicogenomics Database	CCNA1
Comparative Toxicogenomics Database	CCNA2
Comparative Toxicogenomics Database	CCNB1
Comparative Toxicogenomics Database	CCNB2
Comparative Toxicogenomics Database	CCND1
Comparative Toxicogenomics Database	CCND2
Comparative Toxicogenomics Database	CCND3
Comparative Toxicogenomics Database	CCNE1
Comparative Toxicogenomics Database	CCNE2
Comparative Toxicogenomics Database	CCNF
Comparative Toxicogenomics Database	CCNG1
Comparative Toxicogenomics Database	CCNK
Comparative Toxicogenomics Database	CCNL1
Comparative Toxicogenomics Database	CCNT2
Comparative Toxicogenomics Database	CCR1
Comparative Toxicogenomics Database	CCR2
Comparative Toxicogenomics Database	CCR4
Comparative Toxicogenomics Database	CCR5
Comparative Toxicogenomics Database	CCR6
Comparative Toxicogenomics Database	CCR9
Comparative Toxicogenomics Database	CCT2
Comparative Toxicogenomics Database	CCT4
Comparative Toxicogenomics Database	CD14
Comparative Toxicogenomics Database	CD163
Comparative Toxicogenomics Database	CD200R1L
Comparative Toxicogenomics Database	CD209
Comparative Toxicogenomics Database	CD24
Comparative Toxicogenomics Database	CD24A
Comparative Toxicogenomics Database	CD28
Comparative Toxicogenomics Database	CD36
Comparative Toxicogenomics Database	CD38
Comparative Toxicogenomics Database	CD3E
Comparative Toxicogenomics Database	CD4
Comparative Toxicogenomics Database	CD40
Comparative Toxicogenomics Database	CD40LG
Comparative Toxicogenomics Database	CD44
Comparative Toxicogenomics Database	CD53
Comparative Toxicogenomics Database	CD5L
Comparative Toxicogenomics Database	CD68
Comparative Toxicogenomics Database	CD69
Comparative Toxicogenomics Database	CD72

Comparative Toxicogenomics Database	CD74
Comparative Toxicogenomics Database	CD80
Comparative Toxicogenomics Database	CD86
Comparative Toxicogenomics Database	CD8A
Comparative Toxicogenomics Database	CDC123
Comparative Toxicogenomics Database	CDC25A
Comparative Toxicogenomics Database	CDC25B
Comparative Toxicogenomics Database	CDC25C
Comparative Toxicogenomics Database	CDC27
Comparative Toxicogenomics Database	CDC34
Comparative Toxicogenomics Database	CDC42
Comparative Toxicogenomics Database	CDC42BPA
Comparative Toxicogenomics Database	CDC45L
Comparative Toxicogenomics Database	CDC5L
Comparative Toxicogenomics Database	CDC6
Comparative Toxicogenomics Database	CDC7
Comparative Toxicogenomics Database	CDC73
Comparative Toxicogenomics Database	CDCA2
Comparative Toxicogenomics Database	CDCA3
Comparative Toxicogenomics Database	CDCA4
Comparative Toxicogenomics Database	CDCA5
Comparative Toxicogenomics Database	CDCA7
Comparative Toxicogenomics Database	CDCA7L
Comparative Toxicogenomics Database	CDCA8
Comparative Toxicogenomics Database	CDH1
Comparative Toxicogenomics Database	CDH12
Comparative Toxicogenomics Database	CDH2
Comparative Toxicogenomics Database	CDH23
Comparative Toxicogenomics Database	CDH5
Comparative Toxicogenomics Database	CDK1
Comparative Toxicogenomics Database	CDK12
Comparative Toxicogenomics Database	CDK2
Comparative Toxicogenomics Database	CDK4
Comparative Toxicogenomics Database	CDK5
Comparative Toxicogenomics Database	CDK5R1
Comparative Toxicogenomics Database	CDK5RAP2
Comparative Toxicogenomics Database	CDK5RAP3
Comparative Toxicogenomics Database	CDK6
Comparative Toxicogenomics Database	CDK8
Comparative Toxicogenomics Database	CDKAL1
Comparative Toxicogenomics Database	CDKL5
Comparative Toxicogenomics Database	CDKN1A
Comparative Toxicogenomics Database	CDKN1B
Comparative Toxicogenomics Database	CDKN1C
Comparative Toxicogenomics Database	CDKN2A
Comparative Toxicogenomics Database	CDKN2B
Comparative Toxicogenomics Database	CDKN2C
Comparative Toxicogenomics Database	CDKN3
Comparative Toxicogenomics Database	CDT1
Comparative Toxicogenomics Database	CDV3
Comparative Toxicogenomics Database	CDX1
Comparative Toxicogenomics Database	CEACAM1
Comparative Toxicogenomics Database	CEBPA
Comparative Toxicogenomics Database	CEBPB
Comparative Toxicogenomics Database	CEBPD
Comparative Toxicogenomics Database	CEBPG
Comparative Toxicogenomics Database	CED-3
Comparative Toxicogenomics Database	CED-4

Comparative Toxicogenomics Database	CED-9
Comparative Toxicogenomics Database	CEL
Comparative Toxicogenomics Database	CELSR2
Comparative Toxicogenomics Database	CENPA
Comparative Toxicogenomics Database	CENPE
Comparative Toxicogenomics Database	CENPF
Comparative Toxicogenomics Database	CENPH
Comparative Toxicogenomics Database	CENPL
Comparative Toxicogenomics Database	CENPM
Comparative Toxicogenomics Database	CENPN
Comparative Toxicogenomics Database	CENPO
Comparative Toxicogenomics Database	CENPQ
Comparative Toxicogenomics Database	CENPS
Comparative Toxicogenomics Database	CENPU
Comparative Toxicogenomics Database	CENPW
Comparative Toxicogenomics Database	CEP128
Comparative Toxicogenomics Database	CEP152
Comparative Toxicogenomics Database	CEP55
Comparative Toxicogenomics Database	CEP57
Comparative Toxicogenomics Database	CEP78
Comparative Toxicogenomics Database	CERS1
Comparative Toxicogenomics Database	CERS2
Comparative Toxicogenomics Database	CERS4
Comparative Toxicogenomics Database	CERS6
Comparative Toxicogenomics Database	CES2A
Comparative Toxicogenomics Database	CES2C
Comparative Toxicogenomics Database	CES2H
Comparative Toxicogenomics Database	CES3
Comparative Toxicogenomics Database	CES3B
Comparative Toxicogenomics Database	CFAP36
Comparative Toxicogenomics Database	CFD
Comparative Toxicogenomics Database	CFH
Comparative Toxicogenomics Database	CFL1
Comparative Toxicogenomics Database	CFL2
Comparative Toxicogenomics Database	CFLAR
Comparative Toxicogenomics Database	CFP
Comparative Toxicogenomics Database	CFTR
Comparative Toxicogenomics Database	CGA
Comparative Toxicogenomics Database	CGB3
Comparative Toxicogenomics Database	CHAC2
Comparative Toxicogenomics Database	CHAF1A
Comparative Toxicogenomics Database	CHAT
Comparative Toxicogenomics Database	CHCHD2
Comparative Toxicogenomics Database	CHCHD3
Comparative Toxicogenomics Database	CHEK1
Comparative Toxicogenomics Database	CHEK2
Comparative Toxicogenomics Database	CHGA
Comparative Toxicogenomics Database	CHGB
Comparative Toxicogenomics Database	CHI3L1
Comparative Toxicogenomics Database	CHODL
Comparative Toxicogenomics Database	CHP1
Comparative Toxicogenomics Database	CHRM1
Comparative Toxicogenomics Database	CHRM2
Comparative Toxicogenomics Database	CHRM3
Comparative Toxicogenomics Database	CHRNA4
Comparative Toxicogenomics Database	CHRNA5
Comparative Toxicogenomics Database	CHRNB1
Comparative Toxicogenomics Database	CHUK

Comparative Toxicogenomics Database	CIAO2B
Comparative Toxicogenomics Database	CIDEA
Comparative Toxicogenomics Database	CIP2A
Comparative Toxicogenomics Database	CISD2
Comparative Toxicogenomics Database	CIT
Comparative Toxicogenomics Database	CKAP2
Comparative Toxicogenomics Database	CKAP2L
Comparative Toxicogenomics Database	CKAP4
Comparative Toxicogenomics Database	CKS1B
Comparative Toxicogenomics Database	CKS2
Comparative Toxicogenomics Database	CLCA1
Comparative Toxicogenomics Database	CLCF1
Comparative Toxicogenomics Database	CLCN5
Comparative Toxicogenomics Database	CLCN7
Comparative Toxicogenomics Database	CLDN1
Comparative Toxicogenomics Database	CLDN2
Comparative Toxicogenomics Database	CLEC4F
Comparative Toxicogenomics Database	CLEC7A
Comparative Toxicogenomics Database	CLGN
Comparative Toxicogenomics Database	CLIC1
Comparative Toxicogenomics Database	CLIP1
Comparative Toxicogenomics Database	CLK1
Comparative Toxicogenomics Database	CLN3
Comparative Toxicogenomics Database	CLTA
Comparative Toxicogenomics Database	CLU
Comparative Toxicogenomics Database	CLYBL
Comparative Toxicogenomics Database	CMBL
Comparative Toxicogenomics Database	CMC2
Comparative Toxicogenomics Database	CMSS1
Comparative Toxicogenomics Database	CNBP
Comparative Toxicogenomics Database	CNN3
Comparative Toxicogenomics Database	CNOT7
Comparative Toxicogenomics Database	CNST
Comparative Toxicogenomics Database	COBL
Comparative Toxicogenomics Database	COL10A1
Comparative Toxicogenomics Database	COL12A1
Comparative Toxicogenomics Database	COL14A1
Comparative Toxicogenomics Database	COL18A1
Comparative Toxicogenomics Database	COL1A1
Comparative Toxicogenomics Database	COL1A2
Comparative Toxicogenomics Database	COL21A1
Comparative Toxicogenomics Database	COL2A1
Comparative Toxicogenomics Database	COL3A1
Comparative Toxicogenomics Database	COL5A1
Comparative Toxicogenomics Database	COL6A1
Comparative Toxicogenomics Database	COL6A2
Comparative Toxicogenomics Database	COL6A3
Comparative Toxicogenomics Database	COMMD10
Comparative Toxicogenomics Database	COMMD3
Comparative Toxicogenomics Database	COMMD7
Comparative Toxicogenomics Database	COMMD8
Comparative Toxicogenomics Database	COMT
Comparative Toxicogenomics Database	COMTD1
Comparative Toxicogenomics Database	COPS3
Comparative Toxicogenomics Database	COQ2
Comparative Toxicogenomics Database	COQ7
Comparative Toxicogenomics Database	CORO1A
Comparative Toxicogenomics Database	CORO1B

Comparative Toxicogenomics Database	CORO1C
Comparative Toxicogenomics Database	CORO2A
Comparative Toxicogenomics Database	COTL1
Comparative Toxicogenomics Database	COX1
Comparative Toxicogenomics Database	COX11
Comparative Toxicogenomics Database	COX2
Comparative Toxicogenomics Database	COX3
Comparative Toxicogenomics Database	COX4I1
Comparative Toxicogenomics Database	COX4I2
Comparative Toxicogenomics Database	COX5A
Comparative Toxicogenomics Database	COX6A1
Comparative Toxicogenomics Database	COX6B1
Comparative Toxicogenomics Database	COX7A1
Comparative Toxicogenomics Database	COX7A2
Comparative Toxicogenomics Database	COX7B
Comparative Toxicogenomics Database	COX8A
Comparative Toxicogenomics Database	CP110
Comparative Toxicogenomics Database	CPB2
Comparative Toxicogenomics Database	CPE
Comparative Toxicogenomics Database	CPEB2
Comparative Toxicogenomics Database	CPO
Comparative Toxicogenomics Database	CPSF1
Comparative Toxicogenomics Database	CPSF6
Comparative Toxicogenomics Database	CPT1A
Comparative Toxicogenomics Database	CPT1B
Comparative Toxicogenomics Database	CRABP2
Comparative Toxicogenomics Database	CREB1
Comparative Toxicogenomics Database	CREB3L2
Comparative Toxicogenomics Database	CREB3L4
Comparative Toxicogenomics Database	CREBBP
Comparative Toxicogenomics Database	CREBZF
Comparative Toxicogenomics Database	CRELD2
Comparative Toxicogenomics Database	CREM
Comparative Toxicogenomics Database	CRIM1
Comparative Toxicogenomics Database	CRIP1
Comparative Toxicogenomics Database	CRIP2
Comparative Toxicogenomics Database	CRISPLD2
Comparative Toxicogenomics Database	CROT
Comparative Toxicogenomics Database	CRP
Comparative Toxicogenomics Database	CRTC2
Comparative Toxicogenomics Database	CRYAB
Comparative Toxicogenomics Database	CRYBA1
Comparative Toxicogenomics Database	CRYZ
Comparative Toxicogenomics Database	CRYZL1
Comparative Toxicogenomics Database	CS
Comparative Toxicogenomics Database	CSE1L
Comparative Toxicogenomics Database	CSF1
Comparative Toxicogenomics Database	CSF1R
Comparative Toxicogenomics Database	CSF2
Comparative Toxicogenomics Database	CSF3
Comparative Toxicogenomics Database	CSNK1G1
Comparative Toxicogenomics Database	CSNK2A2
Comparative Toxicogenomics Database	CSPRS
Comparative Toxicogenomics Database	CSRNP2
Comparative Toxicogenomics Database	CSRP1
Comparative Toxicogenomics Database	CSRP3
Comparative Toxicogenomics Database	CST3
Comparative Toxicogenomics Database	CST4

Comparative Toxicogenomics Database	CSTA
Comparative Toxicogenomics Database	CSTF1
Comparative Toxicogenomics Database	CTCFLOS
Comparative Toxicogenomics Database	CTDSPL2
Comparative Toxicogenomics Database	CTF1
Comparative Toxicogenomics Database	CTGF
Comparative Toxicogenomics Database	CTHRC1
Comparative Toxicogenomics Database	CTNNA1
Comparative Toxicogenomics Database	CTNNAL1
Comparative Toxicogenomics Database	CTNNB1
Comparative Toxicogenomics Database	CTNND1
Comparative Toxicogenomics Database	CTNND2
Comparative Toxicogenomics Database	CTPS
Comparative Toxicogenomics Database	CTR9
Comparative Toxicogenomics Database	CTSD
Comparative Toxicogenomics Database	CTSH
Comparative Toxicogenomics Database	CTSK
Comparative Toxicogenomics Database	CTSS
Comparative Toxicogenomics Database	CTSV
Comparative Toxicogenomics Database	CTTN
Comparative Toxicogenomics Database	[illegible]TTNBP2NL
Comparative Toxicogenomics Database	CUL5
Comparative Toxicogenomics Database	CUX1
Comparative Toxicogenomics Database	CX3CL1
Comparative Toxicogenomics Database	CXCL1
Comparative Toxicogenomics Database	CXCL10
Comparative Toxicogenomics Database	CXCL12
Comparative Toxicogenomics Database	CXCL14
Comparative Toxicogenomics Database	CXCL15
Comparative Toxicogenomics Database	CXCL2
Comparative Toxicogenomics Database	CXCL3
Comparative Toxicogenomics Database	CXCL5
Comparative Toxicogenomics Database	CXCL6
Comparative Toxicogenomics Database	CXCL8
Comparative Toxicogenomics Database	CXCL9
Comparative Toxicogenomics Database	CXCR3
Comparative Toxicogenomics Database	CXCR4
Comparative Toxicogenomics Database	CYB561
Comparative Toxicogenomics Database	CYB5A
Comparative Toxicogenomics Database	CYB5R1
Comparative Toxicogenomics Database	CYB5R2
Comparative Toxicogenomics Database	CYB5R3
Comparative Toxicogenomics Database	CYBA
Comparative Toxicogenomics Database	CYBB
Comparative Toxicogenomics Database	CYC1
Comparative Toxicogenomics Database	CYCS
Comparative Toxicogenomics Database	CYGB
Comparative Toxicogenomics Database	CYP11A1
Comparative Toxicogenomics Database	CYP-13A6
Comparative Toxicogenomics Database	CYP17A1
Comparative Toxicogenomics Database	CYP19A1
Comparative Toxicogenomics Database	CYP1A
Comparative Toxicogenomics Database	CYP1A1
Comparative Toxicogenomics Database	CYP1A2
Comparative Toxicogenomics Database	CYP1B1
Comparative Toxicogenomics Database	CYP1C1
Comparative Toxicogenomics Database	CYP1C2
Comparative Toxicogenomics Database	CYP21A2

Comparative Toxicogenomics Database	CYP26A1
Comparative Toxicogenomics Database	CYP26B1
Comparative Toxicogenomics Database	CYP27A1
Comparative Toxicogenomics Database	CYP2A12
Comparative Toxicogenomics Database	CYP2A4
Comparative Toxicogenomics Database	CYP2A5
Comparative Toxicogenomics Database	CYP2B1
Comparative Toxicogenomics Database	CYP2B10
Comparative Toxicogenomics Database	CYP2B9
Comparative Toxicogenomics Database	CYP2C1
Comparative Toxicogenomics Database	CYP2C19
Comparative Toxicogenomics Database	CYP2C23
Comparative Toxicogenomics Database	CYP2C29
Comparative Toxicogenomics Database	CYP2C38
Comparative Toxicogenomics Database	CYP2C39
Comparative Toxicogenomics Database	CYP2C8
Comparative Toxicogenomics Database	CYP2C9
Comparative Toxicogenomics Database	CYP2D22
Comparative Toxicogenomics Database	CYP2D6
Comparative Toxicogenomics Database	CYP2D9
Comparative Toxicogenomics Database	CYP2E1
Comparative Toxicogenomics Database	CYP3A11
Comparative Toxicogenomics Database	CYP3A4
Comparative Toxicogenomics Database	CYP4A1
Comparative Toxicogenomics Database	CYP4A10
Comparative Toxicogenomics Database	CYP4A12A
Comparative Toxicogenomics Database	CYP4A14
Comparative Toxicogenomics Database	CYP4A32
Comparative Toxicogenomics Database	CYP4F2
Comparative Toxicogenomics Database	CYP51
Comparative Toxicogenomics Database	CYP7A1
Comparative Toxicogenomics Database	CYP7B1
Comparative Toxicogenomics Database	CYP8B1
Comparative Toxicogenomics Database	CYYR1
Comparative Toxicogenomics Database	DACT1
Comparative Toxicogenomics Database	DAG1
Comparative Toxicogenomics Database	DAPK1
Comparative Toxicogenomics Database	DARS
Comparative Toxicogenomics Database	DARS1
Comparative Toxicogenomics Database	DAXX
Comparative Toxicogenomics Database	DBF4
Comparative Toxicogenomics Database	DBN1
Comparative Toxicogenomics Database	DBR1
Comparative Toxicogenomics Database	DBT
Comparative Toxicogenomics Database	DCAF4
Comparative Toxicogenomics Database	DCAKD
Comparative Toxicogenomics Database	DCK
Comparative Toxicogenomics Database	DCLK1
Comparative Toxicogenomics Database	DCLRE1A
Comparative Toxicogenomics Database	DCLRE1B
Comparative Toxicogenomics Database	DCN
Comparative Toxicogenomics Database	DCPS
Comparative Toxicogenomics Database	DCT
Comparative Toxicogenomics Database	DCTN1
Comparative Toxicogenomics Database	DCTN3
Comparative Toxicogenomics Database	DCTN5
Comparative Toxicogenomics Database	DCTN6
Comparative Toxicogenomics Database	DCUN1D5

Comparative Toxicogenomics Database	DCX
Comparative Toxicogenomics Database	DDAH2
Comparative Toxicogenomics Database	DDB2
Comparative Toxicogenomics Database	DDC
Comparative Toxicogenomics Database	DDIAS
Comparative Toxicogenomics Database	DDIT3
Comparative Toxicogenomics Database	DDIT4
Comparative Toxicogenomics Database	DDOST
Comparative Toxicogenomics Database	DDR2
Comparative Toxicogenomics Database	DDX1
Comparative Toxicogenomics Database	DDX10
Comparative Toxicogenomics Database	DDX17
Comparative Toxicogenomics Database	DDX18
Comparative Toxicogenomics Database	DDX21
Comparative Toxicogenomics Database	DDX23
Comparative Toxicogenomics Database	DDX27
Comparative Toxicogenomics Database	DDX42
Comparative Toxicogenomics Database	DDX49
Comparative Toxicogenomics Database	DDX50
Comparative Toxicogenomics Database	DDX52
Comparative Toxicogenomics Database	DDX54
Comparative Toxicogenomics Database	DDX55
Comparative Toxicogenomics Database	DEFB4A
Comparative Toxicogenomics Database	DEK
Comparative Toxicogenomics Database	DEPDC1
Comparative Toxicogenomics Database	DEPDC6
Comparative Toxicogenomics Database	DEPTOR
Comparative Toxicogenomics Database	DERA
Comparative Toxicogenomics Database	DERL1
Comparative Toxicogenomics Database	DERL3
Comparative Toxicogenomics Database	DES
Comparative Toxicogenomics Database	DFFA
Comparative Toxicogenomics Database	DGKA
Comparative Toxicogenomics Database	DGUOK
Comparative Toxicogenomics Database	DHCR7
Comparative Toxicogenomics Database	DHFR
Comparative Toxicogenomics Database	DHODH
Comparative Toxicogenomics Database	DHRS1
Comparative Toxicogenomics Database	DHRS2
Comparative Toxicogenomics Database	DHRS3
Comparative Toxicogenomics Database	DHRS4
Comparative Toxicogenomics Database	DHRS7
Comparative Toxicogenomics Database	DHX15
Comparative Toxicogenomics Database	DHX16
Comparative Toxicogenomics Database	DHX38
Comparative Toxicogenomics Database	DHX9
Comparative Toxicogenomics Database	DIABLO
Comparative Toxicogenomics Database	DIAPH3
Comparative Toxicogenomics Database	DIO1
Comparative Toxicogenomics Database	DIO2
Comparative Toxicogenomics Database	DKC1
Comparative Toxicogenomics Database	DKK1
Comparative Toxicogenomics Database	DLAT
Comparative Toxicogenomics Database	DLD
Comparative Toxicogenomics Database	DLEU2L
Comparative Toxicogenomics Database	DLG1
Comparative Toxicogenomics Database	DLG4
Comparative Toxicogenomics Database	DLGAP5

Comparative Toxicogenomics Database	DLST
Comparative Toxicogenomics Database	DMBT1
Comparative Toxicogenomics Database	DMD
Comparative Toxicogenomics Database	DMKN
Comparative Toxicogenomics Database	DMXL2
Comparative Toxicogenomics Database	DNA2
Comparative Toxicogenomics Database	DNAAF5
Comparative Toxicogenomics Database	DNAJA1
Comparative Toxicogenomics Database	DNAJB1
Comparative Toxicogenomics Database	DNAJB11
Comparative Toxicogenomics Database	DNAJB14
Comparative Toxicogenomics Database	DNAJB4
Comparative Toxicogenomics Database	DNAJB9
Comparative Toxicogenomics Database	DNAJC9
Comparative Toxicogenomics Database	DNASE2
Comparative Toxicogenomics Database	DNM1L
Comparative Toxicogenomics Database	DNM2
Comparative Toxicogenomics Database	DNMT1
Comparative Toxicogenomics Database	DNMT3A
Comparative Toxicogenomics Database	DNMT3B
Comparative Toxicogenomics Database	DOCK8
Comparative Toxicogenomics Database	DOK4
Comparative Toxicogenomics Database	DONSON
Comparative Toxicogenomics Database	DPM1
Comparative Toxicogenomics Database	DPP7
Comparative Toxicogenomics Database	DRAM1
Comparative Toxicogenomics Database	DSC2
Comparative Toxicogenomics Database	DSCC1
Comparative Toxicogenomics Database	DSG1
Comparative Toxicogenomics Database	DSG2
Comparative Toxicogenomics Database	DSN1
Comparative Toxicogenomics Database	DSTN
Comparative Toxicogenomics Database	DTNA
Comparative Toxicogenomics Database	DTR
Comparative Toxicogenomics Database	DTYMK
Comparative Toxicogenomics Database	DUSP1
Comparative Toxicogenomics Database	DUSP10
Comparative Toxicogenomics Database	DUSP3
Comparative Toxicogenomics Database	DUSP8
Comparative Toxicogenomics Database	DUT
Comparative Toxicogenomics Database	DYNC2H1
Comparative Toxicogenomics Database	DYNLL1
Comparative Toxicogenomics Database	DYNLT3
Comparative Toxicogenomics Database	DYRK1A
Comparative Toxicogenomics Database	E2F1
Comparative Toxicogenomics Database	E2F2
Comparative Toxicogenomics Database	E2F3
Comparative Toxicogenomics Database	E2F7
Comparative Toxicogenomics Database	E2F8
Comparative Toxicogenomics Database	E4F1
Comparative Toxicogenomics Database	EBP
Comparative Toxicogenomics Database	ECE1
Comparative Toxicogenomics Database	ECH1
Comparative Toxicogenomics Database	ECI1
Comparative Toxicogenomics Database	ECI2
Comparative Toxicogenomics Database	ECM1
Comparative Toxicogenomics Database	ECT2
Comparative Toxicogenomics Database	EDARADD

Comparative Toxicogenomics Database	EDC4
Comparative Toxicogenomics Database	EDN1
Comparative Toxicogenomics Database	EDN2
Comparative Toxicogenomics Database	EDRF1
Comparative Toxicogenomics Database	EED
Comparative Toxicogenomics Database	EEF1A1
Comparative Toxicogenomics Database	EEF1A2
Comparative Toxicogenomics Database	EEF1E1
Comparative Toxicogenomics Database	EEF2
Comparative Toxicogenomics Database	EEF2K
Comparative Toxicogenomics Database	EFEMP1
Comparative Toxicogenomics Database	EFHD1
Comparative Toxicogenomics Database	EFNA1
Comparative Toxicogenomics Database	EFNB2
Comparative Toxicogenomics Database	EFTUD2
Comparative Toxicogenomics Database	EGF
Comparative Toxicogenomics Database	EGFR
Comparative Toxicogenomics Database	EGL-1
Comparative Toxicogenomics Database	EGLN1
Comparative Toxicogenomics Database	EGR
Comparative Toxicogenomics Database	EGR1
Comparative Toxicogenomics Database	EGR3
Comparative Toxicogenomics Database	EHBP1L1
Comparative Toxicogenomics Database	EI24
Comparative Toxicogenomics Database	EIF1
Comparative Toxicogenomics Database	EIF2AK2
Comparative Toxicogenomics Database	EIF2AK3
Comparative Toxicogenomics Database	EIF2B4
Comparative Toxicogenomics Database	EIF2S1
Comparative Toxicogenomics Database	EIF3L
Comparative Toxicogenomics Database	EIF4A1
Comparative Toxicogenomics Database	EIF4A2
Comparative Toxicogenomics Database	EIF4B
Comparative Toxicogenomics Database	EIF4E
Comparative Toxicogenomics Database	EIF4EBP1
Comparative Toxicogenomics Database	EIF4G1
Comparative Toxicogenomics Database	EIF4G2
Comparative Toxicogenomics Database	EIF5
Comparative Toxicogenomics Database	EIF5A
Comparative Toxicogenomics Database	EIF6
Comparative Toxicogenomics Database	ELAPOR1
Comparative Toxicogenomics Database	ELAVL1
Comparative Toxicogenomics Database	ELK1
Comparative Toxicogenomics Database	ELOA
Comparative Toxicogenomics Database	ELOVL1
Comparative Toxicogenomics Database	ELOVL2
Comparative Toxicogenomics Database	ELOVL3
Comparative Toxicogenomics Database	ELOVL4
Comparative Toxicogenomics Database	ELOVL5
Comparative Toxicogenomics Database	ELOVL6
Comparative Toxicogenomics Database	ELOVL7
Comparative Toxicogenomics Database	ELP5
Comparative Toxicogenomics Database	EMC2
Comparative Toxicogenomics Database	EMC8
Comparative Toxicogenomics Database	EMC9
Comparative Toxicogenomics Database	EN1
Comparative Toxicogenomics Database	ENC1
Comparative Toxicogenomics Database	ENDOD1

Comparative Toxicogenomics Database	ENDOG
Comparative Toxicogenomics Database	ENO1
Comparative Toxicogenomics Database	ENO2
Comparative Toxicogenomics Database	ENO3
Comparative Toxicogenomics Database	ENPEP
Comparative Toxicogenomics Database	ENPP1
Comparative Toxicogenomics Database	ENPP2
Comparative Toxicogenomics Database	ENPP4
Comparative Toxicogenomics Database	ENTPD1
Comparative Toxicogenomics Database	EP300
Comparative Toxicogenomics Database	EPB41L2
Comparative Toxicogenomics Database	EPB41L3
Comparative Toxicogenomics Database	EPB41L5
Comparative Toxicogenomics Database	EPCAM
Comparative Toxicogenomics Database	EPHA1
Comparative Toxicogenomics Database	EPHA4
Comparative Toxicogenomics Database	EPHX1
Comparative Toxicogenomics Database	EPHX2
Comparative Toxicogenomics Database	EPO
Comparative Toxicogenomics Database	EPOR
Comparative Toxicogenomics Database	EPS15
Comparative Toxicogenomics Database	EPS8
Comparative Toxicogenomics Database	ERBB2
Comparative Toxicogenomics Database	ERBB3
Comparative Toxicogenomics Database	ERBB4
Comparative Toxicogenomics Database	ERC2
Comparative Toxicogenomics Database	ERCC1
Comparative Toxicogenomics Database	ERCC3
Comparative Toxicogenomics Database	ERCC6
Comparative Toxicogenomics Database	ERCC6L
Comparative Toxicogenomics Database	ERLIN2
Comparative Toxicogenomics Database	ERMP1
Comparative Toxicogenomics Database	ERN1
Comparative Toxicogenomics Database	ERO1A
Comparative Toxicogenomics Database	ERRFI1
Comparative Toxicogenomics Database	ES1
Comparative Toxicogenomics Database	ESPL1
Comparative Toxicogenomics Database	ESPN
Comparative Toxicogenomics Database	ESR1
Comparative Toxicogenomics Database	ESR2
Comparative Toxicogenomics Database	ESRRA
Comparative Toxicogenomics Database	ETFA
Comparative Toxicogenomics Database	ETFB
Comparative Toxicogenomics Database	ETHE1
Comparative Toxicogenomics Database	ETNK2
Comparative Toxicogenomics Database	ETS1
Comparative Toxicogenomics Database	ETV4
Comparative Toxicogenomics Database	ETV5
Comparative Toxicogenomics Database	EWSR1
Comparative Toxicogenomics Database	EXO1
Comparative Toxicogenomics Database	EXOSC1
Comparative Toxicogenomics Database	EXOSC10
Comparative Toxicogenomics Database	EXOSC2
Comparative Toxicogenomics Database	EXOSC6
Comparative Toxicogenomics Database	EXOSC7
Comparative Toxicogenomics Database	EYA3
Comparative Toxicogenomics Database	EZH1
Comparative Toxicogenomics Database	EZH2

Comparative Toxicogenomics Database	F13A1
Comparative Toxicogenomics Database	F2
Comparative Toxicogenomics Database	F2R
Comparative Toxicogenomics Database	F3
Comparative Toxicogenomics Database	F8
Comparative Toxicogenomics Database	FABP3
Comparative Toxicogenomics Database	FABP4
Comparative Toxicogenomics Database	FABP6
Comparative Toxicogenomics Database	FABP7
Comparative Toxicogenomics Database	FADD
Comparative Toxicogenomics Database	FADS1
Comparative Toxicogenomics Database	FAH
Comparative Toxicogenomics Database	FAIM
Comparative Toxicogenomics Database	FAM107A
Comparative Toxicogenomics Database	FAM107B
Comparative Toxicogenomics Database	FAM110B
Comparative Toxicogenomics Database	FAM110C
Comparative Toxicogenomics Database	FAM111A
Comparative Toxicogenomics Database	FAM111B
Comparative Toxicogenomics Database	FAM135A
Comparative Toxicogenomics Database	FAM13A
Comparative Toxicogenomics Database	FAM149B1
Comparative Toxicogenomics Database	FAM161B
Comparative Toxicogenomics Database	FAM171B
Comparative Toxicogenomics Database	FAM214A
Comparative Toxicogenomics Database	FAM222B
Comparative Toxicogenomics Database	FAM43A
Comparative Toxicogenomics Database	FAM72A
Comparative Toxicogenomics Database	FANCA
Comparative Toxicogenomics Database	FANCD2
Comparative Toxicogenomics Database	FANCG
Comparative Toxicogenomics Database	FANCI
Comparative Toxicogenomics Database	FANCL
Comparative Toxicogenomics Database	FAR1
Comparative Toxicogenomics Database	FAS
Comparative Toxicogenomics Database	FASL
Comparative Toxicogenomics Database	FASLG
Comparative Toxicogenomics Database	FASN
Comparative Toxicogenomics Database	FBLN5
Comparative Toxicogenomics Database	FBN1
Comparative Toxicogenomics Database	FBP1
Comparative Toxicogenomics Database	FBXO11
Comparative Toxicogenomics Database	FBXO3
Comparative Toxicogenomics Database	FBXO32
Comparative Toxicogenomics Database	FBXO5
Comparative Toxicogenomics Database	FCER1A
Comparative Toxicogenomics Database	FCER1G
Comparative Toxicogenomics Database	FCHO2
Comparative Toxicogenomics Database	FCNA
Comparative Toxicogenomics Database	FDFT1
Comparative Toxicogenomics Database	FDPS
Comparative Toxicogenomics Database	FDX1
Comparative Toxicogenomics Database	FDXR
Comparative Toxicogenomics Database	FEN1
Comparative Toxicogenomics Database	FES
Comparative Toxicogenomics Database	FGD4
Comparative Toxicogenomics Database	FGF1
Comparative Toxicogenomics Database	FGF10

Comparative Toxicogenomics Database	FGF2
Comparative Toxicogenomics Database	FGF21
Comparative Toxicogenomics Database	FGF4
Comparative Toxicogenomics Database	FGF7
Comparative Toxicogenomics Database	FGF8
Comparative Toxicogenomics Database	FGFR1
Comparative Toxicogenomics Database	FGFR3
Comparative Toxicogenomics Database	FGL1
Comparative Toxicogenomics Database	FGR
Comparative Toxicogenomics Database	FHL2
Comparative Toxicogenomics Database	FICD
Comparative Toxicogenomics Database	FIGNL1
Comparative Toxicogenomics Database	FIS1
Comparative Toxicogenomics Database	FITM2
Comparative Toxicogenomics Database	FKBP11
Comparative Toxicogenomics Database	FKBP3
Comparative Toxicogenomics Database	FKBP4
Comparative Toxicogenomics Database	FKBP5
Comparative Toxicogenomics Database	FLOT1
Comparative Toxicogenomics Database	FLT1
Comparative Toxicogenomics Database	FLT3LG
Comparative Toxicogenomics Database	FMNL2
Comparative Toxicogenomics Database	FMO1
Comparative Toxicogenomics Database	FMO4
Comparative Toxicogenomics Database	FMR1
Comparative Toxicogenomics Database	FN1
Comparative Toxicogenomics Database	FNDC3B
Comparative Toxicogenomics Database	FOS
Comparative Toxicogenomics Database	FOXA1
Comparative Toxicogenomics Database	FOXA2
Comparative Toxicogenomics Database	FOXA3
Comparative Toxicogenomics Database	FOXC2
Comparative Toxicogenomics Database	FOXD1
Comparative Toxicogenomics Database	FOXE1
Comparative Toxicogenomics Database	FOXM1
Comparative Toxicogenomics Database	FOXN3
Comparative Toxicogenomics Database	FOXO1
Comparative Toxicogenomics Database	FOXO3
Comparative Toxicogenomics Database	FOXO3A
Comparative Toxicogenomics Database	FOXO4
Comparative Toxicogenomics Database	FOXP3
Comparative Toxicogenomics Database	FOXRED1
Comparative Toxicogenomics Database	FOXRED2
Comparative Toxicogenomics Database	FRMD6
Comparative Toxicogenomics Database	FSHB
Comparative Toxicogenomics Database	FST
Comparative Toxicogenomics Database	FSTL1
Comparative Toxicogenomics Database	FTCD
Comparative Toxicogenomics Database	FTH1
Comparative Toxicogenomics Database	FUCA1
Comparative Toxicogenomics Database	FXN
Comparative Toxicogenomics Database	FXYD2
Comparative Toxicogenomics Database	FYB2
Comparative Toxicogenomics Database	FYN
Comparative Toxicogenomics Database	FYTTD1
Comparative Toxicogenomics Database	FZD2
Comparative Toxicogenomics Database	G0S2
Comparative Toxicogenomics Database	G2E3

Comparative Toxicogenomics Database	G3BP1
Comparative Toxicogenomics Database	G6PC
Comparative Toxicogenomics Database	G6PC3
Comparative Toxicogenomics Database	G6PD
Comparative Toxicogenomics Database	GAB1
Comparative Toxicogenomics Database	GABARAP
Comparative Toxicogenomics Database	GABPA
Comparative Toxicogenomics Database	GADD45A
Comparative Toxicogenomics Database	GADD45B
Comparative Toxicogenomics Database	GADD45G
Comparative Toxicogenomics Database	GALNT10
Comparative Toxicogenomics Database	GALNT4
Comparative Toxicogenomics Database	GALNT7
Comparative Toxicogenomics Database	GAP43
Comparative Toxicogenomics Database	GAPDH
Comparative Toxicogenomics Database	GAPVD1
Comparative Toxicogenomics Database	GAS1
Comparative Toxicogenomics Database	GAS-1
Comparative Toxicogenomics Database	GAS2L3
Comparative Toxicogenomics Database	GAS6
Comparative Toxicogenomics Database	GAST
Comparative Toxicogenomics Database	GATA3
Comparative Toxicogenomics Database	GATM
Comparative Toxicogenomics Database	GBA
Comparative Toxicogenomics Database	GCAT
Comparative Toxicogenomics Database	GCH1
Comparative Toxicogenomics Database	GCHFR
Comparative Toxicogenomics Database	GCK
Comparative Toxicogenomics Database	GCLC
Comparative Toxicogenomics Database	GCLM
Comparative Toxicogenomics Database	GDF1
Comparative Toxicogenomics Database	GDF15
Comparative Toxicogenomics Database	GDNF
Comparative Toxicogenomics Database	GDPD3
Comparative Toxicogenomics Database	GDPD5
Comparative Toxicogenomics Database	GEMIN7
Comparative Toxicogenomics Database	GFAP
Comparative Toxicogenomics Database	GFPT2
Comparative Toxicogenomics Database	GFRA1
Comparative Toxicogenomics Database	GFRA2
Comparative Toxicogenomics Database	GGH
Comparative Toxicogenomics Database	GGNBP2
Comparative Toxicogenomics Database	GGT1
Comparative Toxicogenomics Database	GH1
Comparative Toxicogenomics Database	GHITM
Comparative Toxicogenomics Database	GHR
Comparative Toxicogenomics Database	GHRH
Comparative Toxicogenomics Database	GINS1
Comparative Toxicogenomics Database	GINS2
Comparative Toxicogenomics Database	GINS3
Comparative Toxicogenomics Database	GIT2
Comparative Toxicogenomics Database	GJA1
Comparative Toxicogenomics Database	GJA10
Comparative Toxicogenomics Database	GJA4
Comparative Toxicogenomics Database	GJB2
Comparative Toxicogenomics Database	GLA
Comparative Toxicogenomics Database	GLB1
Comparative Toxicogenomics Database	GLI1

Comparative Toxicogenomics Database	GLIPR2
Comparative Toxicogenomics Database	GLMP
Comparative Toxicogenomics Database	GLO1
Comparative Toxicogenomics Database	GLOD5
Comparative Toxicogenomics Database	GLRX2
Comparative Toxicogenomics Database	GLRX5
Comparative Toxicogenomics Database	GLS
Comparative Toxicogenomics Database	GLS2
Comparative Toxicogenomics Database	GLUL
Comparative Toxicogenomics Database	GMNN
Comparative Toxicogenomics Database	GMPPB
Comparative Toxicogenomics Database	GNAS
Comparative Toxicogenomics Database	GNB1
Comparative Toxicogenomics Database	GNGT2
Comparative Toxicogenomics Database	GNL2
Comparative Toxicogenomics Database	GNPNAT1
Comparative Toxicogenomics Database	GOLIM4
Comparative Toxicogenomics Database	GOT1
Comparative Toxicogenomics Database	GP1BB
Comparative Toxicogenomics Database	GPAA1
Comparative Toxicogenomics Database	GPAM
Comparative Toxicogenomics Database	GPAT4
Comparative Toxicogenomics Database	GPC1
Comparative Toxicogenomics Database	GPC6
Comparative Toxicogenomics Database	GPD1
Comparative Toxicogenomics Database	GPD1L
Comparative Toxicogenomics Database	GPD2
Comparative Toxicogenomics Database	GPER1
Comparative Toxicogenomics Database	GPLD1
Comparative Toxicogenomics Database	GPM6B
Comparative Toxicogenomics Database	GPR142
Comparative Toxicogenomics Database	GPR89
Comparative Toxicogenomics Database	GPRC5A
Comparative Toxicogenomics Database	GPRIN1
Comparative Toxicogenomics Database	GPSM2
Comparative Toxicogenomics Database	GPT
Comparative Toxicogenomics Database	GPX1
Comparative Toxicogenomics Database	GPX2
Comparative Toxicogenomics Database	GPX3
Comparative Toxicogenomics Database	GPX4
Comparative Toxicogenomics Database	GPX8
Comparative Toxicogenomics Database	GRAMD1A
Comparative Toxicogenomics Database	GRB10
Comparative Toxicogenomics Database	GRB2
Comparative Toxicogenomics Database	GREB1
Comparative Toxicogenomics Database	GRHL3
Comparative Toxicogenomics Database	GRIA1
Comparative Toxicogenomics Database	GRIN1
Comparative Toxicogenomics Database	GRIN2B
Comparative Toxicogenomics Database	GRINA
Comparative Toxicogenomics Database	GRN
Comparative Toxicogenomics Database	GRPEL2
Comparative Toxicogenomics Database	GSK3A
Comparative Toxicogenomics Database	GSK3B
Comparative Toxicogenomics Database	GSN
Comparative Toxicogenomics Database	GSPT1
Comparative Toxicogenomics Database	GSR
Comparative Toxicogenomics Database	GSTA1

Comparative Toxicogenomics Database	GSTA2
Comparative Toxicogenomics Database	GSTA3
Comparative Toxicogenomics Database	GSTA4
Comparative Toxicogenomics Database	GSTA5
Comparative Toxicogenomics Database	GSTD1
Comparative Toxicogenomics Database	GSTK1
Comparative Toxicogenomics Database	GSTM1
Comparative Toxicogenomics Database	GSTM3
Comparative Toxicogenomics Database	GSTO1
Comparative Toxicogenomics Database	GSTP1
Comparative Toxicogenomics Database	GSTP3
Comparative Toxicogenomics Database	GSTZ1
Comparative Toxicogenomics Database	GTF2B
Comparative Toxicogenomics Database	GTF2E2
Comparative Toxicogenomics Database	GTF2H4
Comparative Toxicogenomics Database	GTF3A
Comparative Toxicogenomics Database	GTF3C3
Comparative Toxicogenomics Database	GTSE1
Comparative Toxicogenomics Database	GUSB
Comparative Toxicogenomics Database	GYPA
Comparative Toxicogenomics Database	GYS1
Comparative Toxicogenomics Database	GYS2
Comparative Toxicogenomics Database	GZMB
Comparative Toxicogenomics Database	GZMH
Comparative Toxicogenomics Database	H13
Comparative Toxicogenomics Database	H19
Comparative Toxicogenomics Database	H2AC6
Comparative Toxicogenomics Database	H2AX
Comparative Toxicogenomics Database	H2AZ1
Comparative Toxicogenomics Database	H2BC21
Comparative Toxicogenomics Database	H2BC5
Comparative Toxicogenomics Database	H2BC6
Comparative Toxicogenomics Database	H2BC8
Comparative Toxicogenomics Database	H2-D1
Comparative Toxicogenomics Database	H2-K1
Comparative Toxicogenomics Database	H2-M10.1
Comparative Toxicogenomics Database	H2-T23
Comparative Toxicogenomics Database	H3C10
Comparative Toxicogenomics Database	H3C12
Comparative Toxicogenomics Database	H3C13
Comparative Toxicogenomics Database	H3C4
Comparative Toxicogenomics Database	H3F3B
Comparative Toxicogenomics Database	H4C6
Comparative Toxicogenomics Database	HACD2
Comparative Toxicogenomics Database	HADH
Comparative Toxicogenomics Database	HADHB
Comparative Toxicogenomics Database	HAMP
Comparative Toxicogenomics Database	HAO2
Comparative Toxicogenomics Database	HAS1
Comparative Toxicogenomics Database	HASPIN
Comparative Toxicogenomics Database	HAUS1
Comparative Toxicogenomics Database	HAUS8
Comparative Toxicogenomics Database	HAVCR1
Comparative Toxicogenomics Database	HAVCR2
Comparative Toxicogenomics Database	HAX1
Comparative Toxicogenomics Database	HBA1
Comparative Toxicogenomics Database	HBA-A1
Comparative Toxicogenomics Database	HBB

Comparative Toxicogenomics Database	HBB-BS
Comparative Toxicogenomics Database	HBG2
Comparative Toxicogenomics Database	HC
Comparative Toxicogenomics Database	HCCS
Comparative Toxicogenomics Database	HCK
Comparative Toxicogenomics Database	HDAC1
Comparative Toxicogenomics Database	HDAC2
Comparative Toxicogenomics Database	HDAC3
Comparative Toxicogenomics Database	HDAC5
Comparative Toxicogenomics Database	HDAC9
Comparative Toxicogenomics Database	HDGF
Comparative Toxicogenomics Database	HDGFL2
Comparative Toxicogenomics Database	HELLS
Comparative Toxicogenomics Database	HERPUD1
Comparative Toxicogenomics Database	HES1
Comparative Toxicogenomics Database	HEXB
Comparative Toxicogenomics Database	HGF
Comparative Toxicogenomics Database	HHIP
Comparative Toxicogenomics Database	HIBADH
Comparative Toxicogenomics Database	HIF1A
Comparative Toxicogenomics Database	HINT2
Comparative Toxicogenomics Database	HIPK1
Comparative Toxicogenomics Database	HIPK2
Comparative Toxicogenomics Database	HIRIP3
Comparative Toxicogenomics Database	HIVEP1
Comparative Toxicogenomics Database	HJURP
Comparative Toxicogenomics Database	HK1
Comparative Toxicogenomics Database	HK2
Comparative Toxicogenomics Database	HLA-A
Comparative Toxicogenomics Database	HLA-B
Comparative Toxicogenomics Database	HLA-F
Comparative Toxicogenomics Database	HLCS
Comparative Toxicogenomics Database	HMGA2
Comparative Toxicogenomics Database	HMGB1
Comparative Toxicogenomics Database	HMGB2
Comparative Toxicogenomics Database	HMGCR
Comparative Toxicogenomics Database	HMGCS1
Comparative Toxicogenomics Database	HMGCS2
Comparative Toxicogenomics Database	HMGN2
Comparative Toxicogenomics Database	HMMR
Comparative Toxicogenomics Database	HMOX1
Comparative Toxicogenomics Database	HNF1A
Comparative Toxicogenomics Database	HNF1B
Comparative Toxicogenomics Database	HNF4A
Comparative Toxicogenomics Database	HNRNPA1
Comparative Toxicogenomics Database	NRNPA2B1
Comparative Toxicogenomics Database	HNRNPA3
Comparative Toxicogenomics Database	HNRNPAB
Comparative Toxicogenomics Database	HNRNPD
Comparative Toxicogenomics Database	HNRNPDL
Comparative Toxicogenomics Database	HNRNPH2
Comparative Toxicogenomics Database	HNRNPK
Comparative Toxicogenomics Database	HNRNPLL
Comparative Toxicogenomics Database	HNRNPR
Comparative Toxicogenomics Database	HNRNPUL2
Comparative Toxicogenomics Database	HOMER2
Comparative Toxicogenomics Database	HOPX
Comparative Toxicogenomics Database	HOXA1

Comparative Toxicogenomics Database	HOXB9
Comparative Toxicogenomics Database	HOXC6
Comparative Toxicogenomics Database	HP
Comparative Toxicogenomics Database	HPGD
Comparative Toxicogenomics Database	HPRT1
Comparative Toxicogenomics Database	HPX
Comparative Toxicogenomics Database	HR
Comparative Toxicogenomics Database	HRAS
Comparative Toxicogenomics Database	HRG
Comparative Toxicogenomics Database	HS1BP3
Comparative Toxicogenomics Database	HSD11B1
Comparative Toxicogenomics Database	HSD17B12
Comparative Toxicogenomics Database	HSD17B2
Comparative Toxicogenomics Database	HSD17B4
Comparative Toxicogenomics Database	HSD17B7
Comparative Toxicogenomics Database	HSD3B1
Comparative Toxicogenomics Database	HSD3B4
Comparative Toxicogenomics Database	HSD3B5
Comparative Toxicogenomics Database	HSF1
Comparative Toxicogenomics Database	HSP-16.1
Comparative Toxicogenomics Database	HSP27
Comparative Toxicogenomics Database	HSP68
Comparative Toxicogenomics Database	HSP90AA1
Comparative Toxicogenomics Database	HSP90AB1
Comparative Toxicogenomics Database	HSP90B1
Comparative Toxicogenomics Database	HSPA1A
Comparative Toxicogenomics Database	HSPA1B
Comparative Toxicogenomics Database	HSPA2
Comparative Toxicogenomics Database	HSPA4L
Comparative Toxicogenomics Database	HSPA5
Comparative Toxicogenomics Database	HSPA9
Comparative Toxicogenomics Database	HSPB1
Comparative Toxicogenomics Database	HSPB8
Comparative Toxicogenomics Database	HSPD1
Comparative Toxicogenomics Database	HSPE1
Comparative Toxicogenomics Database	HTRA2
Comparative Toxicogenomics Database	HYKK
Comparative Toxicogenomics Database	HYLS1
Comparative Toxicogenomics Database	HYOU1
Comparative Toxicogenomics Database	ICAM1
Comparative Toxicogenomics Database	ID1
Comparative Toxicogenomics Database	ID3
Comparative Toxicogenomics Database	ID4
Comparative Toxicogenomics Database	IDE
Comparative Toxicogenomics Database	IDH2
Comparative Toxicogenomics Database	IDH3A
Comparative Toxicogenomics Database	IDH3B
Comparative Toxicogenomics Database	IDI1
Comparative Toxicogenomics Database	IDO1
Comparative Toxicogenomics Database	IDS
Comparative Toxicogenomics Database	IER2
Comparative Toxicogenomics Database	IER3
Comparative Toxicogenomics Database	IFI30
Comparative Toxicogenomics Database	IFI6
Comparative Toxicogenomics Database	IFIT3
Comparative Toxicogenomics Database	IFITM1
Comparative Toxicogenomics Database	IFITM3
Comparative Toxicogenomics Database	IFNA

Comparative Toxicogenomics Database	IFNA1
Comparative Toxicogenomics Database	IFNB1
Comparative Toxicogenomics Database	IFNG
Comparative Toxicogenomics Database	IFRD1
Comparative Toxicogenomics Database	IFRD2
Comparative Toxicogenomics Database	IFT122
Comparative Toxicogenomics Database	IFT22
Comparative Toxicogenomics Database	IFT27
Comparative Toxicogenomics Database	IFT43
Comparative Toxicogenomics Database	IGF1
Comparative Toxicogenomics Database	IGF1R
Comparative Toxicogenomics Database	IGF2
Comparative Toxicogenomics Database	IGF2BP2
Comparative Toxicogenomics Database	IGF2BP3
Comparative Toxicogenomics Database	IGF2R
Comparative Toxicogenomics Database	IGFBP1
Comparative Toxicogenomics Database	IGFBP2
Comparative Toxicogenomics Database	IGFBP3
Comparative Toxicogenomics Database	IGFBP4
Comparative Toxicogenomics Database	IGFBP5
Comparative Toxicogenomics Database	IGSF6
Comparative Toxicogenomics Database	IHH
Comparative Toxicogenomics Database	IK
Comparative Toxicogenomics Database	IKBKB
Comparative Toxicogenomics Database	IKBKG
Comparative Toxicogenomics Database	IL10
Comparative Toxicogenomics Database	IL12A
Comparative Toxicogenomics Database	IL12B
Comparative Toxicogenomics Database	IL13
Comparative Toxicogenomics Database	IL17A
Comparative Toxicogenomics Database	IL17C
Comparative Toxicogenomics Database	IL17RB
Comparative Toxicogenomics Database	IL18
Comparative Toxicogenomics Database	IL18BP
Comparative Toxicogenomics Database	IL19
Comparative Toxicogenomics Database	IL1A
Comparative Toxicogenomics Database	IL1B
Comparative Toxicogenomics Database	IL1R1
Comparative Toxicogenomics Database	IL1RN
Comparative Toxicogenomics Database	IL2
Comparative Toxicogenomics Database	IL20
Comparative Toxicogenomics Database	IL24
Comparative Toxicogenomics Database	IL27
Comparative Toxicogenomics Database	IL2RA
Comparative Toxicogenomics Database	IL3
Comparative Toxicogenomics Database	IL4
Comparative Toxicogenomics Database	IL5
Comparative Toxicogenomics Database	IL6
Comparative Toxicogenomics Database	IL6ST
Comparative Toxicogenomics Database	IL7R
Comparative Toxicogenomics Database	IL9
Comparative Toxicogenomics Database	ILDR2
Comparative Toxicogenomics Database	ILF2
Comparative Toxicogenomics Database	ILF3
Comparative Toxicogenomics Database	ILP3
Comparative Toxicogenomics Database	ILP5
Comparative Toxicogenomics Database	ILVBL
Comparative Toxicogenomics Database	IMMP1L

Comparative Toxicogenomics Database	IMPDH2
Comparative Toxicogenomics Database	INCENP
Comparative Toxicogenomics Database	INHBA
Comparative Toxicogenomics Database	INPP4A
Comparative Toxicogenomics Database	INPP4B
Comparative Toxicogenomics Database	INS
Comparative Toxicogenomics Database	INS1
Comparative Toxicogenomics Database	INSIG1
Comparative Toxicogenomics Database	INSM1
Comparative Toxicogenomics Database	INSR
Comparative Toxicogenomics Database	INTS2
Comparative Toxicogenomics Database	INTS3
Comparative Toxicogenomics Database	INTS4
Comparative Toxicogenomics Database	INTS5
Comparative Toxicogenomics Database	INTS6
Comparative Toxicogenomics Database	INTS7
Comparative Toxicogenomics Database	INTS8
Comparative Toxicogenomics Database	IPCEF1
Comparative Toxicogenomics Database	IPO13
Comparative Toxicogenomics Database	IPO9
Comparative Toxicogenomics Database	IQCK
Comparative Toxicogenomics Database	IQGAP2
Comparative Toxicogenomics Database	IRAK1
Comparative Toxicogenomics Database	IRAK2
Comparative Toxicogenomics Database	IRF1
Comparative Toxicogenomics Database	IRF2BP2
Comparative Toxicogenomics Database	IRF2BPL
Comparative Toxicogenomics Database	IRF3
Comparative Toxicogenomics Database	IRF6
Comparative Toxicogenomics Database	IRF9
Comparative Toxicogenomics Database	IRGM1
Comparative Toxicogenomics Database	IRS1
Comparative Toxicogenomics Database	IRS2
Comparative Toxicogenomics Database	ISG20
Comparative Toxicogenomics Database	ISOC1
Comparative Toxicogenomics Database	ISYNA1
Comparative Toxicogenomics Database	ITGA1
Comparative Toxicogenomics Database	ITGA2
Comparative Toxicogenomics Database	ITGA2B
Comparative Toxicogenomics Database	ITGA3
Comparative Toxicogenomics Database	ITGA5
Comparative Toxicogenomics Database	ITGAL
Comparative Toxicogenomics Database	ITGAM
Comparative Toxicogenomics Database	ITGAV
Comparative Toxicogenomics Database	ITGB1
Comparative Toxicogenomics Database	ITGB2
Comparative Toxicogenomics Database	ITGB3
Comparative Toxicogenomics Database	ITGB3BP
Comparative Toxicogenomics Database	ITIH3
Comparative Toxicogenomics Database	ITPR1
Comparative Toxicogenomics Database	ITPR3
Comparative Toxicogenomics Database	IVD
Comparative Toxicogenomics Database	IVNS1ABP
Comparative Toxicogenomics Database	IWS1
Comparative Toxicogenomics Database	JADE1
Comparative Toxicogenomics Database	JAK1
Comparative Toxicogenomics Database	JAK2
Comparative Toxicogenomics Database	JDP2

Comparative Toxicogenomics Database	JUN
Comparative Toxicogenomics Database	JUNB
Comparative Toxicogenomics Database	JUND
Comparative Toxicogenomics Database	JUP
Comparative Toxicogenomics Database	KANSL3
Comparative Toxicogenomics Database	KAT2B
Comparative Toxicogenomics Database	KAT5
Comparative Toxicogenomics Database	KATNAL1
Comparative Toxicogenomics Database	KCNA5
Comparative Toxicogenomics Database	KCNB1
Comparative Toxicogenomics Database	KCNH1
Comparative Toxicogenomics Database	KCNH2
Comparative Toxicogenomics Database	KCNH8
Comparative Toxicogenomics Database	KCNIP3
Comparative Toxicogenomics Database	KCNJ11
Comparative Toxicogenomics Database	KCNK12
Comparative Toxicogenomics Database	KCNN4
Comparative Toxicogenomics Database	KCNS1
Comparative Toxicogenomics Database	KCTD20
Comparative Toxicogenomics Database	KDM5A
Comparative Toxicogenomics Database	KDM5B
Comparative Toxicogenomics Database	KDM6A
Comparative Toxicogenomics Database	KDR
Comparative Toxicogenomics Database	KEAP1
Comparative Toxicogenomics Database	KEG1
Comparative Toxicogenomics Database	KHDRBS1
Comparative Toxicogenomics Database	KHSRP
Comparative Toxicogenomics Database	KIF11
Comparative Toxicogenomics Database	KIF14
Comparative Toxicogenomics Database	KIF15
Comparative Toxicogenomics Database	KIF18A
Comparative Toxicogenomics Database	KIF20A
Comparative Toxicogenomics Database	KIF23
Comparative Toxicogenomics Database	KIF2C
Comparative Toxicogenomics Database	KIF7
Comparative Toxicogenomics Database	KIT
Comparative Toxicogenomics Database	KITL
Comparative Toxicogenomics Database	KL
Comparative Toxicogenomics Database	KLF11
Comparative Toxicogenomics Database	KLF13
Comparative Toxicogenomics Database	KLF15
Comparative Toxicogenomics Database	KLF2
Comparative Toxicogenomics Database	KLF4
Comparative Toxicogenomics Database	KLF5
Comparative Toxicogenomics Database	KLF9
Comparative Toxicogenomics Database	KLHL7
Comparative Toxicogenomics Database	KLK2
Comparative Toxicogenomics Database	KLK3
Comparative Toxicogenomics Database	KLRA1
Comparative Toxicogenomics Database	KLRG2
Comparative Toxicogenomics Database	KLRK1
Comparative Toxicogenomics Database	KMO
Comparative Toxicogenomics Database	KNL1
Comparative Toxicogenomics Database	KNSTRN
Comparative Toxicogenomics Database	KNTC1
Comparative Toxicogenomics Database	KPNA2
Comparative Toxicogenomics Database	KPNB1
Comparative Toxicogenomics Database	KRAS

Comparative Toxicogenomics Database	KRI1
Comparative Toxicogenomics Database	KRR1
Comparative Toxicogenomics Database	KRT15
Comparative Toxicogenomics Database	KRT19
Comparative Toxicogenomics Database	KRT20
Comparative Toxicogenomics Database	KRT5
Comparative Toxicogenomics Database	KRT76
Comparative Toxicogenomics Database	KYNU
Comparative Toxicogenomics Database	L1CAM
Comparative Toxicogenomics Database	L2HGDH
Comparative Toxicogenomics Database	LACTB2
Comparative Toxicogenomics Database	LAMB3
Comparative Toxicogenomics Database	LAMC1
Comparative Toxicogenomics Database	LAMP1
Comparative Toxicogenomics Database	LAPTM5
Comparative Toxicogenomics Database	LBP
Comparative Toxicogenomics Database	LBR
Comparative Toxicogenomics Database	LCAT
Comparative Toxicogenomics Database	LCLAT1
Comparative Toxicogenomics Database	LCN2
Comparative Toxicogenomics Database	LCOR
Comparative Toxicogenomics Database	LDAH
Comparative Toxicogenomics Database	LDHB
Comparative Toxicogenomics Database	LDLR
Comparative Toxicogenomics Database	LEF1
Comparative Toxicogenomics Database	LEFTY2
Comparative Toxicogenomics Database	LEP
Comparative Toxicogenomics Database	LEPR
Comparative Toxicogenomics Database	LGALS1
Comparative Toxicogenomics Database	LGALS3
Comparative Toxicogenomics Database	LGR5
Comparative Toxicogenomics Database	LHB
Comparative Toxicogenomics Database	LHCGR
Comparative Toxicogenomics Database	LHX2
Comparative Toxicogenomics Database	LIF
Comparative Toxicogenomics Database	LIFR
Comparative Toxicogenomics Database	LIG1
Comparative Toxicogenomics Database	LIMA1
Comparative Toxicogenomics Database	LIMK1
Comparative Toxicogenomics Database	LIN54
Comparative Toxicogenomics Database	LIN7A
Comparative Toxicogenomics Database	LINC00052
Comparative Toxicogenomics Database	LINC00460
Comparative Toxicogenomics Database	LINC01588
Comparative Toxicogenomics Database	LIPE
Comparative Toxicogenomics Database	LIPG
Comparative Toxicogenomics Database	LITAF
Comparative Toxicogenomics Database	LMAN2
Comparative Toxicogenomics Database	LMCD1
Comparative Toxicogenomics Database	LMF2
Comparative Toxicogenomics Database	LMNA
Comparative Toxicogenomics Database	LMNB1
Comparative Toxicogenomics Database	LMNB2
Comparative Toxicogenomics Database	LNPK
Comparative Toxicogenomics Database	LONP1
Comparative Toxicogenomics Database	LPCAT3
Comparative Toxicogenomics Database	LPGAT1
Comparative Toxicogenomics Database	LPIN1

Comparative Toxicogenomics Database	LPIN2
Comparative Toxicogenomics Database	LPL
Comparative Toxicogenomics Database	LPP
Comparative Toxicogenomics Database	LRIG1
Comparative Toxicogenomics Database	LRP1
Comparative Toxicogenomics Database	LRP5
Comparative Toxicogenomics Database	LRP8
Comparative Toxicogenomics Database	LRR1
Comparative Toxicogenomics Database	LRRC39
Comparative Toxicogenomics Database	LRRC49
Comparative Toxicogenomics Database	LRRFIP1
Comparative Toxicogenomics Database	LSAMP
Comparative Toxicogenomics Database	LSM3
Comparative Toxicogenomics Database	LSM4
Comparative Toxicogenomics Database	LSM8
Comparative Toxicogenomics Database	LST1
Comparative Toxicogenomics Database	LTA
Comparative Toxicogenomics Database	LTBR
Comparative Toxicogenomics Database	LTC4S
Comparative Toxicogenomics Database	LUM
Comparative Toxicogenomics Database	LXN
Comparative Toxicogenomics Database	LY6D
Comparative Toxicogenomics Database	LY6G
Comparative Toxicogenomics Database	LYAR
Comparative Toxicogenomics Database	LYPD6
Comparative Toxicogenomics Database	LYRM4
Comparative Toxicogenomics Database	LYRM9
Comparative Toxicogenomics Database	LYZ
Comparative Toxicogenomics Database	MAD2L1
Comparative Toxicogenomics Database	MAD2L1BP
Comparative Toxicogenomics Database	MADD
Comparative Toxicogenomics Database	MAF
Comparative Toxicogenomics Database	MAFF
Comparative Toxicogenomics Database	MAG
Comparative Toxicogenomics Database	MAGOHB
Comparative Toxicogenomics Database	MAK16
Comparative Toxicogenomics Database	MALAT1
Comparative Toxicogenomics Database	MAMDC2
Comparative Toxicogenomics Database	MANEA
Comparative Toxicogenomics Database	MANSC1
Comparative Toxicogenomics Database	MAOA
Comparative Toxicogenomics Database	MAOB
Comparative Toxicogenomics Database	MAP1A
Comparative Toxicogenomics Database	MAP1B
Comparative Toxicogenomics Database	MAP1LC3A
Comparative Toxicogenomics Database	MAP1LC3B
Comparative Toxicogenomics Database	MAP2
Comparative Toxicogenomics Database	MAP2K1
Comparative Toxicogenomics Database	MAP2K2
Comparative Toxicogenomics Database	MAP2K3
Comparative Toxicogenomics Database	MAP2K5
Comparative Toxicogenomics Database	MAP2K6
Comparative Toxicogenomics Database	MAP3K1
Comparative Toxicogenomics Database	MAP3K14
Comparative Toxicogenomics Database	MAP3K4
Comparative Toxicogenomics Database	MAP3K5
Comparative Toxicogenomics Database	MAP4
Comparative Toxicogenomics Database	MAPK1

Comparative Toxicogenomics Database	MAPK13
Comparative Toxicogenomics Database	MAPK14
Comparative Toxicogenomics Database	MAPK3
Comparative Toxicogenomics Database	MAPK7
Comparative Toxicogenomics Database	MAPK8
Comparative Toxicogenomics Database	MAPK9
Comparative Toxicogenomics Database	MAPT
Comparative Toxicogenomics Database	MARCHF5
Comparative Toxicogenomics Database	MARCKSL1
Comparative Toxicogenomics Database	MARK2
Comparative Toxicogenomics Database	MASTL
Comparative Toxicogenomics Database	MAT2A
Comparative Toxicogenomics Database	MAT2B
Comparative Toxicogenomics Database	MATN3
Comparative Toxicogenomics Database	MATR3
Comparative Toxicogenomics Database	MAX
Comparative Toxicogenomics Database	MBD1
Comparative Toxicogenomics Database	MBD2
Comparative Toxicogenomics Database	MBD3
Comparative Toxicogenomics Database	MBOAT7
Comparative Toxicogenomics Database	MBTPS1
Comparative Toxicogenomics Database	MC1R
Comparative Toxicogenomics Database	MC2R
Comparative Toxicogenomics Database	MCAM
Comparative Toxicogenomics Database	MCCC1
Comparative Toxicogenomics Database	MCEE
Comparative Toxicogenomics Database	MCL1
Comparative Toxicogenomics Database	MCM10
Comparative Toxicogenomics Database	MCM2
Comparative Toxicogenomics Database	MCM3
Comparative Toxicogenomics Database	MCM4
Comparative Toxicogenomics Database	MCM5
Comparative Toxicogenomics Database	MCM6
Comparative Toxicogenomics Database	MCM7
Comparative Toxicogenomics Database	MCM8
Comparative Toxicogenomics Database	MCPT1
Comparative Toxicogenomics Database	MCPT2
Comparative Toxicogenomics Database	MCPT4
Comparative Toxicogenomics Database	MDFIC
Comparative Toxicogenomics Database	MDM2
Comparative Toxicogenomics Database	ME1
Comparative Toxicogenomics Database	MEAF6
Comparative Toxicogenomics Database	MECP2
Comparative Toxicogenomics Database	MECR
Comparative Toxicogenomics Database	MED13
Comparative Toxicogenomics Database	MED24
Comparative Toxicogenomics Database	MED28
Comparative Toxicogenomics Database	MED29
Comparative Toxicogenomics Database	MEDAG
Comparative Toxicogenomics Database	MEF2C
Comparative Toxicogenomics Database	MELK
Comparative Toxicogenomics Database	MEMO1
Comparative Toxicogenomics Database	MEP1A
Comparative Toxicogenomics Database	MET
Comparative Toxicogenomics Database	METAP2
Comparative Toxicogenomics Database	METRNL
Comparative Toxicogenomics Database	METTL18
Comparative Toxicogenomics Database	METTL7A

Comparative Toxicogenomics Database	METTL7A1
Comparative Toxicogenomics Database	MFAP5
Comparative Toxicogenomics Database	MFGE8
Comparative Toxicogenomics Database	MFN1
Comparative Toxicogenomics Database	MFN2
Comparative Toxicogenomics Database	MFSD4A
Comparative Toxicogenomics Database	MGA
Comparative Toxicogenomics Database	MGAT4A
Comparative Toxicogenomics Database	MGLL
Comparative Toxicogenomics Database	MGMT
Comparative Toxicogenomics Database	MGP
Comparative Toxicogenomics Database	MGST3
Comparative Toxicogenomics Database	MIA
Comparative Toxicogenomics Database	MICA
Comparative Toxicogenomics Database	MICB
Comparative Toxicogenomics Database	MID1IP1
Comparative Toxicogenomics Database	MIR1
Comparative Toxicogenomics Database	MIR100
Comparative Toxicogenomics Database	MIR101A
Comparative Toxicogenomics Database	MIR101B
Comparative Toxicogenomics Database	MIR103A1
Comparative Toxicogenomics Database	MIR103A2
Comparative Toxicogenomics Database	MIR106A
Comparative Toxicogenomics Database	MIR106B
Comparative Toxicogenomics Database	MIR10A
Comparative Toxicogenomics Database	MIR10B
Comparative Toxicogenomics Database	MIR122
Comparative Toxicogenomics Database	MIR125A
Comparative Toxicogenomics Database	MIR126
Comparative Toxicogenomics Database	MIR129-2
Comparative Toxicogenomics Database	MIR130A
Comparative Toxicogenomics Database	MIR133A1
Comparative Toxicogenomics Database	MIR133B
Comparative Toxicogenomics Database	MIR134
Comparative Toxicogenomics Database	MIR136
Comparative Toxicogenomics Database	MIR137
Comparative Toxicogenomics Database	MIR142
Comparative Toxicogenomics Database	MIR143
Comparative Toxicogenomics Database	MIR145
Comparative Toxicogenomics Database	MIR146A
Comparative Toxicogenomics Database	MIR146B
Comparative Toxicogenomics Database	MIR148A
Comparative Toxicogenomics Database	MIR152
Comparative Toxicogenomics Database	MIR155
Comparative Toxicogenomics Database	MIR15A
Comparative Toxicogenomics Database	MIR16-1
Comparative Toxicogenomics Database	MIR17
Comparative Toxicogenomics Database	MIR17HG
Comparative Toxicogenomics Database	MIR181A2
Comparative Toxicogenomics Database	MIR181C
Comparative Toxicogenomics Database	MIR183
Comparative Toxicogenomics Database	MIR185
Comparative Toxicogenomics Database	MIR186
Comparative Toxicogenomics Database	MIR1902
Comparative Toxicogenomics Database	MIR193A
Comparative Toxicogenomics Database	MIR194
Comparative Toxicogenomics Database	MIR195
Comparative Toxicogenomics Database	MIR196A1

Comparative Toxicogenomics Database	MIR198
Comparative Toxicogenomics Database	MIR1983
Comparative Toxicogenomics Database	MIR19A
Comparative Toxicogenomics Database	MIR200B
Comparative Toxicogenomics Database	MIR200C
Comparative Toxicogenomics Database	MIR202
Comparative Toxicogenomics Database	MIR203
Comparative Toxicogenomics Database	MIR203A
Comparative Toxicogenomics Database	MIR204
Comparative Toxicogenomics Database	MIR205
Comparative Toxicogenomics Database	MIR206
Comparative Toxicogenomics Database	MIR208A
Comparative Toxicogenomics Database	MIR20A
Comparative Toxicogenomics Database	MIR20B
Comparative Toxicogenomics Database	MIR21
Comparative Toxicogenomics Database	MIR214
Comparative Toxicogenomics Database	MIR218
Comparative Toxicogenomics Database	MIR22
Comparative Toxicogenomics Database	MIR221
Comparative Toxicogenomics Database	MIR23A
Comparative Toxicogenomics Database	MIR23B
Comparative Toxicogenomics Database	MIR24-1
Comparative Toxicogenomics Database	MIR25
Comparative Toxicogenomics Database	MIR26A1
Comparative Toxicogenomics Database	MIR27A
Comparative Toxicogenomics Database	MIR27B
Comparative Toxicogenomics Database	MIR28
Comparative Toxicogenomics Database	MIR29A
Comparative Toxicogenomics Database	MIR29B
Comparative Toxicogenomics Database	MIR29C
Comparative Toxicogenomics Database	MIR301B
Comparative Toxicogenomics Database	MIR302B
Comparative Toxicogenomics Database	MIR302D
Comparative Toxicogenomics Database	MIR30A
Comparative Toxicogenomics Database	MIR30C1
Comparative Toxicogenomics Database	MIR30D
Comparative Toxicogenomics Database	MIR30E
Comparative Toxicogenomics Database	MIR31
Comparative Toxicogenomics Database	MIR323
Comparative Toxicogenomics Database	MIR323A
Comparative Toxicogenomics Database	MIR324
Comparative Toxicogenomics Database	MIR326
Comparative Toxicogenomics Database	MIR328
Comparative Toxicogenomics Database	MIR337
Comparative Toxicogenomics Database	MIR339
Comparative Toxicogenomics Database	MIR33A
Comparative Toxicogenomics Database	MIR340
Comparative Toxicogenomics Database	MIR342
Comparative Toxicogenomics Database	MIR345
Comparative Toxicogenomics Database	MIR34A
Comparative Toxicogenomics Database	MIR34C
Comparative Toxicogenomics Database	MIR350
Comparative Toxicogenomics Database	MIR351
Comparative Toxicogenomics Database	MIR352
Comparative Toxicogenomics Database	MIR362
Comparative Toxicogenomics Database	MIR363
Comparative Toxicogenomics Database	MIR369
Comparative Toxicogenomics Database	MIR374B

Comparative Toxicogenomics Database	MIR375
Comparative Toxicogenomics Database	MIR376B
Comparative Toxicogenomics Database	MIR378A
Comparative Toxicogenomics Database	MIR3911
Comparative Toxicogenomics Database	MIR3916
Comparative Toxicogenomics Database	MIR421
Comparative Toxicogenomics Database	MIR424
Comparative Toxicogenomics Database	MIR434
Comparative Toxicogenomics Database	IR4435-2HG
Comparative Toxicogenomics Database	MIR450A1
Comparative Toxicogenomics Database	MIR450B
Comparative Toxicogenomics Database	MIR455
Comparative Toxicogenomics Database	MIR466D
Comparative Toxicogenomics Database	MIR487B
Comparative Toxicogenomics Database	MIR489
Comparative Toxicogenomics Database	MIR491
Comparative Toxicogenomics Database	MIR494
Comparative Toxicogenomics Database	MIR497
Comparative Toxicogenomics Database	MIR501
Comparative Toxicogenomics Database	MIR503
Comparative Toxicogenomics Database	MIR504
Comparative Toxicogenomics Database	MIR507
Comparative Toxicogenomics Database	MIR517A
Comparative Toxicogenomics Database	MIR518B
Comparative Toxicogenomics Database	MIR518C
Comparative Toxicogenomics Database	MIR518F
Comparative Toxicogenomics Database	MIR519B
Comparative Toxicogenomics Database	MIR525
Comparative Toxicogenomics Database	MIR532
Comparative Toxicogenomics Database	MIR539
Comparative Toxicogenomics Database	MIR542
Comparative Toxicogenomics Database	MIR548V
Comparative Toxicogenomics Database	MIR569
Comparative Toxicogenomics Database	MIR570
Comparative Toxicogenomics Database	MIR571
Comparative Toxicogenomics Database	MIR572
Comparative Toxicogenomics Database	MIR574
Comparative Toxicogenomics Database	MIR576
Comparative Toxicogenomics Database	MIR582
Comparative Toxicogenomics Database	MIR584
Comparative Toxicogenomics Database	MIR590
Comparative Toxicogenomics Database	MIR601
Comparative Toxicogenomics Database	MIR615
Comparative Toxicogenomics Database	MIR622
Comparative Toxicogenomics Database	MIR625
Comparative Toxicogenomics Database	MIR629
Comparative Toxicogenomics Database	MIR631
Comparative Toxicogenomics Database	MIR632
Comparative Toxicogenomics Database	MIR638
Comparative Toxicogenomics Database	MIR639
Comparative Toxicogenomics Database	MIR645
Comparative Toxicogenomics Database	MIR652
Comparative Toxicogenomics Database	MIR657
Comparative Toxicogenomics Database	MIR659
Comparative Toxicogenomics Database	MIR663A
Comparative Toxicogenomics Database	MIR667
Comparative Toxicogenomics Database	MIR671
Comparative Toxicogenomics Database	MIR687

Comparative Toxicogenomics Database	MIR708
Comparative Toxicogenomics Database	MIR760
Comparative Toxicogenomics Database	MIR888
Comparative Toxicogenomics Database	MIR9
Comparative Toxicogenomics Database	MIR92A2
Comparative Toxicogenomics Database	MIR92B
Comparative Toxicogenomics Database	MIR93
Comparative Toxicogenomics Database	MIR96
Comparative Toxicogenomics Database	MIR98
Comparative Toxicogenomics Database	MIRLET7C
Comparative Toxicogenomics Database	MIRLET7C-1
Comparative Toxicogenomics Database	MIRLET7E
Comparative Toxicogenomics Database	MIS18BP1
Comparative Toxicogenomics Database	MITF
Comparative Toxicogenomics Database	MKI67
Comparative Toxicogenomics Database	MKNK2
Comparative Toxicogenomics Database	MLYCD
Comparative Toxicogenomics Database	MMD
Comparative Toxicogenomics Database	MMD2
Comparative Toxicogenomics Database	MME
Comparative Toxicogenomics Database	MMP1
Comparative Toxicogenomics Database	MMP13
Comparative Toxicogenomics Database	MMP16
Comparative Toxicogenomics Database	MMP2
Comparative Toxicogenomics Database	MMP3
Comparative Toxicogenomics Database	MMP7
Comparative Toxicogenomics Database	MMP9
Comparative Toxicogenomics Database	MMUT
Comparative Toxicogenomics Database	MNDA
Comparative Toxicogenomics Database	MORC3
Comparative Toxicogenomics Database	MORF4L1
Comparative Toxicogenomics Database	MOS
Comparative Toxicogenomics Database	MPHOSPH10
Comparative Toxicogenomics Database	MPHOSPH9
Comparative Toxicogenomics Database	MPO
Comparative Toxicogenomics Database	MPPED2
Comparative Toxicogenomics Database	MPRIP
Comparative Toxicogenomics Database	MPST
Comparative Toxicogenomics Database	MPZL2
Comparative Toxicogenomics Database	MR1
Comparative Toxicogenomics Database	MRC1
Comparative Toxicogenomics Database	MRE11
Comparative Toxicogenomics Database	MRE11A
Comparative Toxicogenomics Database	MRPL11
Comparative Toxicogenomics Database	MRPL12
Comparative Toxicogenomics Database	MRPL13
Comparative Toxicogenomics Database	MRPL15
Comparative Toxicogenomics Database	MRPL16
Comparative Toxicogenomics Database	MRPL19
Comparative Toxicogenomics Database	MRPL20
Comparative Toxicogenomics Database	MRPL22
Comparative Toxicogenomics Database	MRPL24
Comparative Toxicogenomics Database	MRPL27
Comparative Toxicogenomics Database	MRPL3
Comparative Toxicogenomics Database	MRPL33
Comparative Toxicogenomics Database	MRPL34
Comparative Toxicogenomics Database	MRPL36
Comparative Toxicogenomics Database	MRPL4

Comparative Toxicogenomics Database	MRPL40
Comparative Toxicogenomics Database	MRPL44
Comparative Toxicogenomics Database	MRPL46
Comparative Toxicogenomics Database	MRPL48
Comparative Toxicogenomics Database	MRPL49
Comparative Toxicogenomics Database	MRPS11
Comparative Toxicogenomics Database	MRPS12
Comparative Toxicogenomics Database	MRPS15
Comparative Toxicogenomics Database	MRPS16
Comparative Toxicogenomics Database	MRPS17
Comparative Toxicogenomics Database	MRPS18A
Comparative Toxicogenomics Database	MRPS18B
Comparative Toxicogenomics Database	MRPS18C
Comparative Toxicogenomics Database	MRPS2
Comparative Toxicogenomics Database	MRPS27
Comparative Toxicogenomics Database	MRPS28
Comparative Toxicogenomics Database	MRPS31
Comparative Toxicogenomics Database	MRPS33
Comparative Toxicogenomics Database	MRPS6
Comparative Toxicogenomics Database	MRPS7
Comparative Toxicogenomics Database	MRTFB
Comparative Toxicogenomics Database	MRTO4
Comparative Toxicogenomics Database	MS4A6A
Comparative Toxicogenomics Database	MS4A6BL
Comparative Toxicogenomics Database	MSH2
Comparative Toxicogenomics Database	MSH6
Comparative Toxicogenomics Database	MSI1
Comparative Toxicogenomics Database	MSI2
Comparative Toxicogenomics Database	MSMO1
Comparative Toxicogenomics Database	MSR1
Comparative Toxicogenomics Database	MSRA
Comparative Toxicogenomics Database	MT1
Comparative Toxicogenomics Database	MT1H
Comparative Toxicogenomics Database	MT1X
Comparative Toxicogenomics Database	MTA1
Comparative Toxicogenomics Database	MTCH2
Comparative Toxicogenomics Database	MTERF1A
Comparative Toxicogenomics Database	MTF1
Comparative Toxicogenomics Database	MTFP1
Comparative Toxicogenomics Database	MTFR2
Comparative Toxicogenomics Database	MTHFD1
Comparative Toxicogenomics Database	MTHFD1L
Comparative Toxicogenomics Database	MTHFD2
Comparative Toxicogenomics Database	MTMR4
Comparative Toxicogenomics Database	MTMR7
Comparative Toxicogenomics Database	MTNR1A
Comparative Toxicogenomics Database	MTNR1B
Comparative Toxicogenomics Database	MTOR
Comparative Toxicogenomics Database	MTPN
Comparative Toxicogenomics Database	MTREX
Comparative Toxicogenomics Database	MTUS1
Comparative Toxicogenomics Database	MTX1
Comparative Toxicogenomics Database	MUC1
Comparative Toxicogenomics Database	MUC5AC
Comparative Toxicogenomics Database	MUG2
Comparative Toxicogenomics Database	MUP1
Comparative Toxicogenomics Database	MUP14
Comparative Toxicogenomics Database	MUP21

Comparative Toxicogenomics Database	MUP3
Comparative Toxicogenomics Database	MUP5
Comparative Toxicogenomics Database	MUP9
Comparative Toxicogenomics Database	MVP
Comparative Toxicogenomics Database	MYBBP1A
Comparative Toxicogenomics Database	MYBL2
Comparative Toxicogenomics Database	MYC
Comparative Toxicogenomics Database	MYCN
Comparative Toxicogenomics Database	MYD88
Comparative Toxicogenomics Database	MYF5
Comparative Toxicogenomics Database	MYG1
Comparative Toxicogenomics Database	MYH10
Comparative Toxicogenomics Database	MYH11
Comparative Toxicogenomics Database	MYH14
Comparative Toxicogenomics Database	MYH6
Comparative Toxicogenomics Database	MYH7
Comparative Toxicogenomics Database	MYL1
Comparative Toxicogenomics Database	MYL2
Comparative Toxicogenomics Database	MYLK
Comparative Toxicogenomics Database	MYLPF
Comparative Toxicogenomics Database	MYO10
Comparative Toxicogenomics Database	MYO18A
Comparative Toxicogenomics Database	MYO1B
Comparative Toxicogenomics Database	MYO5A
Comparative Toxicogenomics Database	MYOCD
Comparative Toxicogenomics Database	MYOD1
Comparative Toxicogenomics Database	MYOG
Comparative Toxicogenomics Database	MZT1
Comparative Toxicogenomics Database	N4BP1
Comparative Toxicogenomics Database	NAB1
Comparative Toxicogenomics Database	NABP1
Comparative Toxicogenomics Database	NACA
Comparative Toxicogenomics Database	NADK2
Comparative Toxicogenomics Database	NAIP
Comparative Toxicogenomics Database	NAMPT
Comparative Toxicogenomics Database	NANOG
Comparative Toxicogenomics Database	NANOS1
Comparative Toxicogenomics Database	NASP
Comparative Toxicogenomics Database	NAT1
Comparative Toxicogenomics Database	NAT8
Comparative Toxicogenomics Database	NBEA
Comparative Toxicogenomics Database	NBN
Comparative Toxicogenomics Database	NCAPG
Comparative Toxicogenomics Database	NCAPG2
Comparative Toxicogenomics Database	NCAPH
Comparative Toxicogenomics Database	NCBP1
Comparative Toxicogenomics Database	NCF1
Comparative Toxicogenomics Database	NCF2
Comparative Toxicogenomics Database	NCL
Comparative Toxicogenomics Database	NCOA1
Comparative Toxicogenomics Database	NCOA2
Comparative Toxicogenomics Database	NCOA3
Comparative Toxicogenomics Database	NCOA4
Comparative Toxicogenomics Database	NCOA5
Comparative Toxicogenomics Database	NCOA6
Comparative Toxicogenomics Database	NCOA7
Comparative Toxicogenomics Database	NCOR1
Comparative Toxicogenomics Database	NCOR2

Comparative Toxicogenomics Database	ND1
Comparative Toxicogenomics Database	NDC1
Comparative Toxicogenomics Database	NDC80
Comparative Toxicogenomics Database	NDE1
Comparative Toxicogenomics Database	NDRG1
Comparative Toxicogenomics Database	NDUFA1
Comparative Toxicogenomics Database	NDUFA13
Comparative Toxicogenomics Database	NDUFA2
Comparative Toxicogenomics Database	NDUFA3
Comparative Toxicogenomics Database	NDUFA4
Comparative Toxicogenomics Database	NDUFA5
Comparative Toxicogenomics Database	NDUFA6
Comparative Toxicogenomics Database	NDUFA7
Comparative Toxicogenomics Database	NDUFAF3
Comparative Toxicogenomics Database	NDUFB3
Comparative Toxicogenomics Database	NDUFB4
Comparative Toxicogenomics Database	NDUFB5
Comparative Toxicogenomics Database	NDUFB7
Comparative Toxicogenomics Database	NDUFB8
Comparative Toxicogenomics Database	NDUFB9
Comparative Toxicogenomics Database	NDUFC1
Comparative Toxicogenomics Database	NDUFS1
Comparative Toxicogenomics Database	NDUFS3
Comparative Toxicogenomics Database	NDUFS4
Comparative Toxicogenomics Database	NDUFS5
Comparative Toxicogenomics Database	NDUFS6
Comparative Toxicogenomics Database	NDUFV1
Comparative Toxicogenomics Database	NDUFV2
Comparative Toxicogenomics Database	NEBL
Comparative Toxicogenomics Database	NECAP2
Comparative Toxicogenomics Database	NECTIN2
Comparative Toxicogenomics Database	NEDD4
Comparative Toxicogenomics Database	NEDD4L
Comparative Toxicogenomics Database	NEDD9
Comparative Toxicogenomics Database	NEFH
Comparative Toxicogenomics Database	NEIL2
Comparative Toxicogenomics Database	NEIL3
Comparative Toxicogenomics Database	NEK2
Comparative Toxicogenomics Database	NELL2
Comparative Toxicogenomics Database	NEMP1
Comparative Toxicogenomics Database	NEO1
Comparative Toxicogenomics Database	NES
Comparative Toxicogenomics Database	NFAT5
Comparative Toxicogenomics Database	NFATC1
Comparative Toxicogenomics Database	NFE2L1
Comparative Toxicogenomics Database	NFE2L2
Comparative Toxicogenomics Database	NFE2L3
Comparative Toxicogenomics Database	NFIX
Comparative Toxicogenomics Database	NFKB1
Comparative Toxicogenomics Database	NFKB2
Comparative Toxicogenomics Database	NFKBIA
Comparative Toxicogenomics Database	NFKBIB
Comparative Toxicogenomics Database	NFKBIZ
Comparative Toxicogenomics Database	NFS1
Comparative Toxicogenomics Database	NGF
Comparative Toxicogenomics Database	NGFR
Comparative Toxicogenomics Database	NID2
Comparative Toxicogenomics Database	NIM1K

Comparative Toxicogenomics Database	NINL
Comparative Toxicogenomics Database	NKAIN1
Comparative Toxicogenomics Database	NKX1-2
Comparative Toxicogenomics Database	NKX2-1
Comparative Toxicogenomics Database	NKX2-2
Comparative Toxicogenomics Database	NKX3-1
Comparative Toxicogenomics Database	NKX6-1
Comparative Toxicogenomics Database	NLRP1
Comparative Toxicogenomics Database	NLRP12
Comparative Toxicogenomics Database	NLRP3
Comparative Toxicogenomics Database	NME1
Comparative Toxicogenomics Database	NME4
Comparative Toxicogenomics Database	NMNAT1
Comparative Toxicogenomics Database	NMRAL1
Comparative Toxicogenomics Database	NMRK1
Comparative Toxicogenomics Database	NMU
Comparative Toxicogenomics Database	NNMT
Comparative Toxicogenomics Database	NOC2L
Comparative Toxicogenomics Database	NOC3L
Comparative Toxicogenomics Database	NOD1
Comparative Toxicogenomics Database	NOL11
Comparative Toxicogenomics Database	NOL12
Comparative Toxicogenomics Database	NOLC1
Comparative Toxicogenomics Database	NOM1
Comparative Toxicogenomics Database	NOP16
Comparative Toxicogenomics Database	NOP56
Comparative Toxicogenomics Database	NOS
Comparative Toxicogenomics Database	NOS1
Comparative Toxicogenomics Database	NOS2
Comparative Toxicogenomics Database	NOS2A
Comparative Toxicogenomics Database	NOS3
Comparative Toxicogenomics Database	NOTCH1
Comparative Toxicogenomics Database	NOTCH2
Comparative Toxicogenomics Database	NOX1
Comparative Toxicogenomics Database	NOX2
Comparative Toxicogenomics Database	NOX3
Comparative Toxicogenomics Database	NOX4
Comparative Toxicogenomics Database	NPAS2
Comparative Toxicogenomics Database	NPAS3
Comparative Toxicogenomics Database	NPC1
Comparative Toxicogenomics Database	NPC2
Comparative Toxicogenomics Database	NPHS1
Comparative Toxicogenomics Database	NPHS2
Comparative Toxicogenomics Database	NPPA
Comparative Toxicogenomics Database	NPPB
Comparative Toxicogenomics Database	NPPC
Comparative Toxicogenomics Database	NPY1R
Comparative Toxicogenomics Database	NQO1
Comparative Toxicogenomics Database	NQO2
Comparative Toxicogenomics Database	NR0B2
Comparative Toxicogenomics Database	NR1D1
Comparative Toxicogenomics Database	NR1H3
Comparative Toxicogenomics Database	NR1H4
Comparative Toxicogenomics Database	NR1I2
Comparative Toxicogenomics Database	NR1I3
Comparative Toxicogenomics Database	NR2F2
Comparative Toxicogenomics Database	NR3C1
Comparative Toxicogenomics Database	NRAS

Comparative Toxicogenomics Database	NREP
Comparative Toxicogenomics Database	NRF1
Comparative Toxicogenomics Database	NRG1
Comparative Toxicogenomics Database	NRIP1
Comparative Toxicogenomics Database	NRM
Comparative Toxicogenomics Database	NSD2
Comparative Toxicogenomics Database	NT5C3
Comparative Toxicogenomics Database	NT5DC2
Comparative Toxicogenomics Database	NT5M
Comparative Toxicogenomics Database	NTMT1
Comparative Toxicogenomics Database	NTN4
Comparative Toxicogenomics Database	NTPCR
Comparative Toxicogenomics Database	NTRK3
Comparative Toxicogenomics Database	NTS
Comparative Toxicogenomics Database	NUBP2
Comparative Toxicogenomics Database	NUCB1
Comparative Toxicogenomics Database	NUCB2
Comparative Toxicogenomics Database	NUDCD2
Comparative Toxicogenomics Database	NUDT1
Comparative Toxicogenomics Database	NUDT12
Comparative Toxicogenomics Database	NUDT16
Comparative Toxicogenomics Database	NUDT16L1
Comparative Toxicogenomics Database	NUDT7
Comparative Toxicogenomics Database	NUF2
Comparative Toxicogenomics Database	NUFIP2
Comparative Toxicogenomics Database	NUMA1
Comparative Toxicogenomics Database	NUP107
Comparative Toxicogenomics Database	NUP153
Comparative Toxicogenomics Database	NUP155
Comparative Toxicogenomics Database	NUP210
Comparative Toxicogenomics Database	NUP98
Comparative Toxicogenomics Database	NUPR1
Comparative Toxicogenomics Database	NUSAP1
Comparative Toxicogenomics Database	NVL
Comparative Toxicogenomics Database	NYNRIN
Comparative Toxicogenomics Database	OASL
Comparative Toxicogenomics Database	OCLN
Comparative Toxicogenomics Database	ODC1
Comparative Toxicogenomics Database	OGDH
Comparative Toxicogenomics Database	OGDHL
Comparative Toxicogenomics Database	OGFR
Comparative Toxicogenomics Database	OGG1
Comparative Toxicogenomics Database	OIP5
Comparative Toxicogenomics Database	OLFM1
Comparative Toxicogenomics Database	OLFML3
Comparative Toxicogenomics Database	OLFR166
Comparative Toxicogenomics Database	OLR1
Comparative Toxicogenomics Database	OPA1
Comparative Toxicogenomics Database	OPTN
Comparative Toxicogenomics Database	ORC1
Comparative Toxicogenomics Database	ORC6
Comparative Toxicogenomics Database	ORM1
Comparative Toxicogenomics Database	ORMDL2
Comparative Toxicogenomics Database	ORMDL3
Comparative Toxicogenomics Database	OSBPL3
Comparative Toxicogenomics Database	OSER1-DT
Comparative Toxicogenomics Database	OVAL
Comparative Toxicogenomics Database	OXCT1

Comparative Toxicogenomics Database	OXTR
Comparative Toxicogenomics Database	P2RX4
Comparative Toxicogenomics Database	P2RX7
Comparative Toxicogenomics Database	P2RY12
Comparative Toxicogenomics Database	P4HA1
Comparative Toxicogenomics Database	PAF1
Comparative Toxicogenomics Database	PAH
Comparative Toxicogenomics Database	PAICS
Comparative Toxicogenomics Database	PAK1
Comparative Toxicogenomics Database	PAK2
Comparative Toxicogenomics Database	PAK3
Comparative Toxicogenomics Database	ALM2AKAP2
Comparative Toxicogenomics Database	PAPPA
Comparative Toxicogenomics Database	PAPSS1
Comparative Toxicogenomics Database	PAQR5
Comparative Toxicogenomics Database	PARM1
Comparative Toxicogenomics Database	PARN
Comparative Toxicogenomics Database	PARP1
Comparative Toxicogenomics Database	PARP2
Comparative Toxicogenomics Database	PARPBP
Comparative Toxicogenomics Database	PARVB
Comparative Toxicogenomics Database	PAX3
Comparative Toxicogenomics Database	PAX8
Comparative Toxicogenomics Database	PBDC1
Comparative Toxicogenomics Database	PBK
Comparative Toxicogenomics Database	PBRM1
Comparative Toxicogenomics Database	PBX1
Comparative Toxicogenomics Database	PC
Comparative Toxicogenomics Database	PCCB
Comparative Toxicogenomics Database	PCK1
Comparative Toxicogenomics Database	PCK2
Comparative Toxicogenomics Database	PCLAF
Comparative Toxicogenomics Database	PCNA
Comparative Toxicogenomics Database	PCP4
Comparative Toxicogenomics Database	PCSK1
Comparative Toxicogenomics Database	PCSK5
Comparative Toxicogenomics Database	PCSK6
Comparative Toxicogenomics Database	PCSK9
Comparative Toxicogenomics Database	PDCD11
Comparative Toxicogenomics Database	PDCD2
Comparative Toxicogenomics Database	PDCD4
Comparative Toxicogenomics Database	PDCD7
Comparative Toxicogenomics Database	PDE2A
Comparative Toxicogenomics Database	PDE4D
Comparative Toxicogenomics Database	PDE5A
Comparative Toxicogenomics Database	PDGFB
Comparative Toxicogenomics Database	PDGFRA
Comparative Toxicogenomics Database	PDGFRB
Comparative Toxicogenomics Database	PDHA1
Comparative Toxicogenomics Database	PDHB
Comparative Toxicogenomics Database	PDHX
Comparative Toxicogenomics Database	PDIA3
Comparative Toxicogenomics Database	PDIA4
Comparative Toxicogenomics Database	PDK1
Comparative Toxicogenomics Database	PDK4
Comparative Toxicogenomics Database	PDL1
Comparative Toxicogenomics Database	PDLIM1
Comparative Toxicogenomics Database	PDPK1

Comparative Toxicogenomics Database	PDSS1
Comparative Toxicogenomics Database	PDX1
Comparative Toxicogenomics Database	PDXP
Comparative Toxicogenomics Database	PDYN
Comparative Toxicogenomics Database	PDZD2
Comparative Toxicogenomics Database	PDZK1
Comparative Toxicogenomics Database	PDZK1IP1
Comparative Toxicogenomics Database	PEBP1
Comparative Toxicogenomics Database	PECAM1
Comparative Toxicogenomics Database	PEG3
Comparative Toxicogenomics Database	PELP1
Comparative Toxicogenomics Database	PERP
Comparative Toxicogenomics Database	PF4
Comparative Toxicogenomics Database	PFKFB1
Comparative Toxicogenomics Database	PFKFB3
Comparative Toxicogenomics Database	PFKM
Comparative Toxicogenomics Database	PFKP
Comparative Toxicogenomics Database	PGAM1
Comparative Toxicogenomics Database	PGF
Comparative Toxicogenomics Database	PGK1
Comparative Toxicogenomics Database	PGM2L1
Comparative Toxicogenomics Database	PGP
Comparative Toxicogenomics Database	PGPEP1
Comparative Toxicogenomics Database	PGR
Comparative Toxicogenomics Database	PGRMC1
Comparative Toxicogenomics Database	PHF14
Comparative Toxicogenomics Database	PHF19
Comparative Toxicogenomics Database	PHF5A
Comparative Toxicogenomics Database	PHLDB2
Comparative Toxicogenomics Database	PHTF1
Comparative Toxicogenomics Database	PHTF2
Comparative Toxicogenomics Database	PHYH
Comparative Toxicogenomics Database	PI4K2B
Comparative Toxicogenomics Database	PIAS3
Comparative Toxicogenomics Database	PID1
Comparative Toxicogenomics Database	PIDD1
Comparative Toxicogenomics Database	PIGU
Comparative Toxicogenomics Database	PIGZ
Comparative Toxicogenomics Database	PIK3C3
Comparative Toxicogenomics Database	PIK3CA
Comparative Toxicogenomics Database	PIK3CB
Comparative Toxicogenomics Database	PIK3CG
Comparative Toxicogenomics Database	PIK3R1
Comparative Toxicogenomics Database	PIK3R3
Comparative Toxicogenomics Database	PIM1
Comparative Toxicogenomics Database	PIM3
Comparative Toxicogenomics Database	PIMREG
Comparative Toxicogenomics Database	PIN4
Comparative Toxicogenomics Database	PINK1
Comparative Toxicogenomics Database	PINX1
Comparative Toxicogenomics Database	PIP4K2C
Comparative Toxicogenomics Database	PIP4P2
Comparative Toxicogenomics Database	PIWIL3
Comparative Toxicogenomics Database	PKD2
Comparative Toxicogenomics Database	PKIB
Comparative Toxicogenomics Database	PKM
Comparative Toxicogenomics Database	PKN2
Comparative Toxicogenomics Database	PKP2

Comparative Toxicogenomics Database	PKP3
Comparative Toxicogenomics Database	PLA2G2A
Comparative Toxicogenomics Database	PLA2G4A
Comparative Toxicogenomics Database	PLA2G6
Comparative Toxicogenomics Database	PLAAT3
Comparative Toxicogenomics Database	PLAT
Comparative Toxicogenomics Database	PLAU
Comparative Toxicogenomics Database	PLAUR
Comparative Toxicogenomics Database	PLCB3
Comparative Toxicogenomics Database	PLCD1
Comparative Toxicogenomics Database	PLCG1
Comparative Toxicogenomics Database	PLCG2
Comparative Toxicogenomics Database	PLD1
Comparative Toxicogenomics Database	PLEC
Comparative Toxicogenomics Database	PLEK
Comparative Toxicogenomics Database	PLEKHA5
Comparative Toxicogenomics Database	PLEKHF1
Comparative Toxicogenomics Database	PLEKHF2
Comparative Toxicogenomics Database	PLIN2
Comparative Toxicogenomics Database	PLIN4
Comparative Toxicogenomics Database	PLK1
Comparative Toxicogenomics Database	PLK4
Comparative Toxicogenomics Database	PLN
Comparative Toxicogenomics Database	PLOD2
Comparative Toxicogenomics Database	PLOD3
Comparative Toxicogenomics Database	PLP2
Comparative Toxicogenomics Database	PLPP2
Comparative Toxicogenomics Database	PLPPR2
Comparative Toxicogenomics Database	PLSCR1
Comparative Toxicogenomics Database	PLXDC1
Comparative Toxicogenomics Database	PLXDC2
Comparative Toxicogenomics Database	PLXNA2
Comparative Toxicogenomics Database	PLXNB2
Comparative Toxicogenomics Database	PMAIP1
Comparative Toxicogenomics Database	PMEPA1
Comparative Toxicogenomics Database	PML
Comparative Toxicogenomics Database	PMP22
Comparative Toxicogenomics Database	PMPCB
Comparative Toxicogenomics Database	PMS1
Comparative Toxicogenomics Database	PMVK
Comparative Toxicogenomics Database	PNN
Comparative Toxicogenomics Database	PNP
Comparative Toxicogenomics Database	PNPLA2
Comparative Toxicogenomics Database	PNPLA5
Comparative Toxicogenomics Database	PNPO
Comparative Toxicogenomics Database	PNRC1
Comparative Toxicogenomics Database	POLA1
Comparative Toxicogenomics Database	POLA2
Comparative Toxicogenomics Database	POLD1
Comparative Toxicogenomics Database	POLD2
Comparative Toxicogenomics Database	POLD3
Comparative Toxicogenomics Database	POLDIP2
Comparative Toxicogenomics Database	POLDIP3
Comparative Toxicogenomics Database	POLE2
Comparative Toxicogenomics Database	POLI
Comparative Toxicogenomics Database	POLR2B
Comparative Toxicogenomics Database	POLR2D
Comparative Toxicogenomics Database	POLR2E

Comparative Toxicogenomics Database	POLR2G
Comparative Toxicogenomics Database	POLR2I
Comparative Toxicogenomics Database	POLR2J
Comparative Toxicogenomics Database	POLR2K
Comparative Toxicogenomics Database	POMC
Comparative Toxicogenomics Database	POMP
Comparative Toxicogenomics Database	PON1
Comparative Toxicogenomics Database	POR
Comparative Toxicogenomics Database	POU1F1
Comparative Toxicogenomics Database	POU5F1
Comparative Toxicogenomics Database	PPAN
Comparative Toxicogenomics Database	PPARA
Comparative Toxicogenomics Database	PPARD
Comparative Toxicogenomics Database	PPARG
Comparative Toxicogenomics Database	PPARGC1A
Comparative Toxicogenomics Database	PPAT
Comparative Toxicogenomics Database	PPBP
Comparative Toxicogenomics Database	PPID
Comparative Toxicogenomics Database	PPIF
Comparative Toxicogenomics Database	PPL
Comparative Toxicogenomics Database	PPM1A
Comparative Toxicogenomics Database	PPM1E
Comparative Toxicogenomics Database	PPP1CA
Comparative Toxicogenomics Database	PPP1CB
Comparative Toxicogenomics Database	PPP1R10
Comparative Toxicogenomics Database	PPP1R12A
Comparative Toxicogenomics Database	PPP1R1B
Comparative Toxicogenomics Database	PPP1R2
Comparative Toxicogenomics Database	PPP1R3B
Comparative Toxicogenomics Database	PPP1R3C
Comparative Toxicogenomics Database	PPP2CA
Comparative Toxicogenomics Database	PPP2CB
Comparative Toxicogenomics Database	PPP2R5A
Comparative Toxicogenomics Database	PPP2R5D
Comparative Toxicogenomics Database	PPT1
Comparative Toxicogenomics Database	PQBP1
Comparative Toxicogenomics Database	PQN-54
Comparative Toxicogenomics Database	PQN-76
Comparative Toxicogenomics Database	PQN-78
Comparative Toxicogenomics Database	PQN-91
Comparative Toxicogenomics Database	PRDM16
Comparative Toxicogenomics Database	PRDM2
Comparative Toxicogenomics Database	PRDX1
Comparative Toxicogenomics Database	PRDX2
Comparative Toxicogenomics Database	PRDX6
Comparative Toxicogenomics Database	PREB
Comparative Toxicogenomics Database	PRF1
Comparative Toxicogenomics Database	PRIM1
Comparative Toxicogenomics Database	PRIM2
Comparative Toxicogenomics Database	PRKAA1
Comparative Toxicogenomics Database	PRKAA2
Comparative Toxicogenomics Database	PRKAB2
Comparative Toxicogenomics Database	PRKACA
Comparative Toxicogenomics Database	PRKACB
Comparative Toxicogenomics Database	PRKAG1
Comparative Toxicogenomics Database	PRKAR1A
Comparative Toxicogenomics Database	PRKAR2A
Comparative Toxicogenomics Database	PRKAR2B

Comparative Toxicogenomics Database	PRKCA
Comparative Toxicogenomics Database	PRKCB
Comparative Toxicogenomics Database	PRKCD
Comparative Toxicogenomics Database	PRKCG
Comparative Toxicogenomics Database	PRKCI
Comparative Toxicogenomics Database	PRKCQ
Comparative Toxicogenomics Database	PRKD1
Comparative Toxicogenomics Database	PRKG1
Comparative Toxicogenomics Database	PRKN
Comparative Toxicogenomics Database	PRKX
Comparative Toxicogenomics Database	PRL
Comparative Toxicogenomics Database	PRMT1
Comparative Toxicogenomics Database	PRMT6
Comparative Toxicogenomics Database	PRODH2
Comparative Toxicogenomics Database	PROM2
Comparative Toxicogenomics Database	PRORSD1
Comparative Toxicogenomics Database	PROS1
Comparative Toxicogenomics Database	PRPF3
Comparative Toxicogenomics Database	PRPF4
Comparative Toxicogenomics Database	PRPF4B
Comparative Toxicogenomics Database	PRPF6
Comparative Toxicogenomics Database	PRPF8
Comparative Toxicogenomics Database	PRPS2
Comparative Toxicogenomics Database	PRPSAP1
Comparative Toxicogenomics Database	PRRC2A
Comparative Toxicogenomics Database	PRSS16
Comparative Toxicogenomics Database	PRSS23
Comparative Toxicogenomics Database	PRSS8
Comparative Toxicogenomics Database	PRX2540-1
Comparative Toxicogenomics Database	PRX2540-2
Comparative Toxicogenomics Database	PRX6005
Comparative Toxicogenomics Database	PRY
Comparative Toxicogenomics Database	PSAT1
Comparative Toxicogenomics Database	PSEN2
Comparative Toxicogenomics Database	PSIP1
Comparative Toxicogenomics Database	PSMA5
Comparative Toxicogenomics Database	PSMB10
Comparative Toxicogenomics Database	PSMB5
Comparative Toxicogenomics Database	PSMB7
Comparative Toxicogenomics Database	PSMB8
Comparative Toxicogenomics Database	PSMC3
Comparative Toxicogenomics Database	PSMC3IP
Comparative Toxicogenomics Database	PSMC6
Comparative Toxicogenomics Database	PSMD10
Comparative Toxicogenomics Database	PSMD12
Comparative Toxicogenomics Database	PSMD14
Comparative Toxicogenomics Database	PSME4
Comparative Toxicogenomics Database	PSMG3
Comparative Toxicogenomics Database	PSRC1
Comparative Toxicogenomics Database	PTCH1
Comparative Toxicogenomics Database	PTCHD4
Comparative Toxicogenomics Database	PTEN
Comparative Toxicogenomics Database	PTGDS
Comparative Toxicogenomics Database	PTGER1
Comparative Toxicogenomics Database	PTGER4
Comparative Toxicogenomics Database	PTGES
Comparative Toxicogenomics Database	PTGES2
Comparative Toxicogenomics Database	PTGES3

Comparative Toxicogenomics Database	PTGS1
Comparative Toxicogenomics Database	PTGS2
Comparative Toxicogenomics Database	PTK2
Comparative Toxicogenomics Database	PTK2B
Comparative Toxicogenomics Database	PTMA
Comparative Toxicogenomics Database	PTPMT1
Comparative Toxicogenomics Database	PTPN1
Comparative Toxicogenomics Database	PTPN11
Comparative Toxicogenomics Database	PTPN12
Comparative Toxicogenomics Database	PTPN21
Comparative Toxicogenomics Database	PTPRA
Comparative Toxicogenomics Database	PTPRE
Comparative Toxicogenomics Database	PTPRF
Comparative Toxicogenomics Database	PTPRG
Comparative Toxicogenomics Database	PTPRN2
Comparative Toxicogenomics Database	PTPRR
Comparative Toxicogenomics Database	PTRH2
Comparative Toxicogenomics Database	PTTG1
Comparative Toxicogenomics Database	PTX3
Comparative Toxicogenomics Database	PUF60
Comparative Toxicogenomics Database	PUS1
Comparative Toxicogenomics Database	PWP1
Comparative Toxicogenomics Database	PWP2
Comparative Toxicogenomics Database	PXDN
Comparative Toxicogenomics Database	PXMP4
Comparative Toxicogenomics Database	PXN
Comparative Toxicogenomics Database	PYCARD
Comparative Toxicogenomics Database	PYCR2
Comparative Toxicogenomics Database	PYGL
Comparative Toxicogenomics Database	PYGO2
Comparative Toxicogenomics Database	PYROXD2
Comparative Toxicogenomics Database	QARS
Comparative Toxicogenomics Database	QK
Comparative Toxicogenomics Database	QPRT
Comparative Toxicogenomics Database	RAB10
Comparative Toxicogenomics Database	RAB11A
Comparative Toxicogenomics Database	RAB11B
Comparative Toxicogenomics Database	RAB14
Comparative Toxicogenomics Database	RAB18
Comparative Toxicogenomics Database	RAB1A
Comparative Toxicogenomics Database	RAB20
Comparative Toxicogenomics Database	RAB21
Comparative Toxicogenomics Database	RAB23
Comparative Toxicogenomics Database	RAB27B
Comparative Toxicogenomics Database	RAB2A
Comparative Toxicogenomics Database	RAB2B
Comparative Toxicogenomics Database	RAB30
Comparative Toxicogenomics Database	RAB31
Comparative Toxicogenomics Database	RAB32
Comparative Toxicogenomics Database	RAB33B
Comparative Toxicogenomics Database	RAB38
Comparative Toxicogenomics Database	RAB3D
Comparative Toxicogenomics Database	RAB43
Comparative Toxicogenomics Database	RAB4B
Comparative Toxicogenomics Database	RAB5B
Comparative Toxicogenomics Database	RAB5C
Comparative Toxicogenomics Database	RAB6A
Comparative Toxicogenomics Database	RAB7

Comparative Toxicogenomics Database	RAB7A
Comparative Toxicogenomics Database	RAB8A
Comparative Toxicogenomics Database	RAB8B
Comparative Toxicogenomics Database	RAB9
Comparative Toxicogenomics Database	RABGAP1L
Comparative Toxicogenomics Database	RAC1
Comparative Toxicogenomics Database	RAC2
Comparative Toxicogenomics Database	RAC3
Comparative Toxicogenomics Database	RACGAP1
Comparative Toxicogenomics Database	RAD18
Comparative Toxicogenomics Database	RAD23B
Comparative Toxicogenomics Database	RAD50
Comparative Toxicogenomics Database	RAD51
Comparative Toxicogenomics Database	RAD51AP1
Comparative Toxicogenomics Database	RAD51C
Comparative Toxicogenomics Database	RAD54B
Comparative Toxicogenomics Database	RAD54L
Comparative Toxicogenomics Database	RAD9B
Comparative Toxicogenomics Database	RAET1E
Comparative Toxicogenomics Database	RAF1
Comparative Toxicogenomics Database	RAN
Comparative Toxicogenomics Database	RANBP2
Comparative Toxicogenomics Database	RANBP3
Comparative Toxicogenomics Database	RANGAP1
Comparative Toxicogenomics Database	RAP1A
Comparative Toxicogenomics Database	RAP1B
Comparative Toxicogenomics Database	RAP2A
Comparative Toxicogenomics Database	RAPH1
Comparative Toxicogenomics Database	RARA
Comparative Toxicogenomics Database	RARRES1
Comparative Toxicogenomics Database	RASA2
Comparative Toxicogenomics Database	RASD1
Comparative Toxicogenomics Database	RASGRF1
Comparative Toxicogenomics Database	RASGRP1
Comparative Toxicogenomics Database	RASGRP2
Comparative Toxicogenomics Database	RASSF4
Comparative Toxicogenomics Database	RB1
Comparative Toxicogenomics Database	RB1CC1
Comparative Toxicogenomics Database	RBBP8
Comparative Toxicogenomics Database	RBM13
Comparative Toxicogenomics Database	RBM15
Comparative Toxicogenomics Database	RBM19
Comparative Toxicogenomics Database	RBM22
Comparative Toxicogenomics Database	RBM24
Comparative Toxicogenomics Database	RBM25
Comparative Toxicogenomics Database	RBM28
Comparative Toxicogenomics Database	RBM3
Comparative Toxicogenomics Database	RBM33
Comparative Toxicogenomics Database	RBM34
Comparative Toxicogenomics Database	RBM39
Comparative Toxicogenomics Database	RBM5
Comparative Toxicogenomics Database	RBM8A
Comparative Toxicogenomics Database	RBMS1
Comparative Toxicogenomics Database	RBP4
Comparative Toxicogenomics Database	RCAN1
Comparative Toxicogenomics Database	RCBTB1
Comparative Toxicogenomics Database	RCCD1
Comparative Toxicogenomics Database	RCOR1

Comparative Toxicogenomics Database	RDH10
Comparative Toxicogenomics Database	RDH11
Comparative Toxicogenomics Database	RDH13
Comparative Toxicogenomics Database	RDH14
Comparative Toxicogenomics Database	RDH2
Comparative Toxicogenomics Database	RECK
Comparative Toxicogenomics Database	RECQL4
Comparative Toxicogenomics Database	REEP4
Comparative Toxicogenomics Database	REEP5
Comparative Toxicogenomics Database	REG3A
Comparative Toxicogenomics Database	REL
Comparative Toxicogenomics Database	RELA
Comparative Toxicogenomics Database	RELB
Comparative Toxicogenomics Database	RELN
Comparative Toxicogenomics Database	REPS1
Comparative Toxicogenomics Database	RERG
Comparative Toxicogenomics Database	REST
Comparative Toxicogenomics Database	RET
Comparative Toxicogenomics Database	RETN
Comparative Toxicogenomics Database	RFC2
Comparative Toxicogenomics Database	RFC3
Comparative Toxicogenomics Database	RFC4
Comparative Toxicogenomics Database	RFC5
Comparative Toxicogenomics Database	RFTN1
Comparative Toxicogenomics Database	RFX2
Comparative Toxicogenomics Database	RGN
Comparative Toxicogenomics Database	RGS13
Comparative Toxicogenomics Database	RGS4
Comparative Toxicogenomics Database	RGS5
Comparative Toxicogenomics Database	RHBDF1
Comparative Toxicogenomics Database	RHNO1
Comparative Toxicogenomics Database	RHOA
Comparative Toxicogenomics Database	RHOBTB1
Comparative Toxicogenomics Database	RHOC
Comparative Toxicogenomics Database	RHOJ
Comparative Toxicogenomics Database	RHOQ
Comparative Toxicogenomics Database	RHOT1
Comparative Toxicogenomics Database	RHOU
Comparative Toxicogenomics Database	RIBC2
Comparative Toxicogenomics Database	RIOK3
Comparative Toxicogenomics Database	RIPK1
Comparative Toxicogenomics Database	RIPK3
Comparative Toxicogenomics Database	RIPOR2
Comparative Toxicogenomics Database	RMI1
Comparative Toxicogenomics Database	RMI2
Comparative Toxicogenomics Database	RNASEH2A
Comparative Toxicogenomics Database	RNF113A
Comparative Toxicogenomics Database	RNF128
Comparative Toxicogenomics Database	RNF144A
Comparative Toxicogenomics Database	RNF185
Comparative Toxicogenomics Database	RNF26
Comparative Toxicogenomics Database	RNF4
Comparative Toxicogenomics Database	RNF6
Comparative Toxicogenomics Database	RNFT2
Comparative Toxicogenomics Database	RNH1
Comparative Toxicogenomics Database	RNPC3
Comparative Toxicogenomics Database	RNU11
Comparative Toxicogenomics Database	RNU6ATAC

Comparative Toxicogenomics Database	ROCK1
Comparative Toxicogenomics Database	ROCK2
Comparative Toxicogenomics Database	RPA1
Comparative Toxicogenomics Database	RPA2
Comparative Toxicogenomics Database	RPA3
Comparative Toxicogenomics Database	RPF2
Comparative Toxicogenomics Database	RPL17
Comparative Toxicogenomics Database	RPL19
Comparative Toxicogenomics Database	RPL3
Comparative Toxicogenomics Database	RPL32
Comparative Toxicogenomics Database	RPL39
Comparative Toxicogenomics Database	RPL6
Comparative Toxicogenomics Database	RPLP2
Comparative Toxicogenomics Database	RPRD1B
Comparative Toxicogenomics Database	RPRD2
Comparative Toxicogenomics Database	RPRM
Comparative Toxicogenomics Database	RPS19
Comparative Toxicogenomics Database	RPS19BP1
Comparative Toxicogenomics Database	RPS21
Comparative Toxicogenomics Database	RPS3A
Comparative Toxicogenomics Database	RPS6
Comparative Toxicogenomics Database	RPS6KA1
Comparative Toxicogenomics Database	RPS6KB1
Comparative Toxicogenomics Database	RPTOR
Comparative Toxicogenomics Database	RRAGA
Comparative Toxicogenomics Database	RRBP1
Comparative Toxicogenomics Database	RRM1
Comparative Toxicogenomics Database	RRM2
Comparative Toxicogenomics Database	RRM2B
Comparative Toxicogenomics Database	RRP1
Comparative Toxicogenomics Database	RRP1B
Comparative Toxicogenomics Database	RSAD2
Comparative Toxicogenomics Database	RSBN1
Comparative Toxicogenomics Database	RSBN1L
Comparative Toxicogenomics Database	RSPRY1
Comparative Toxicogenomics Database	RSRC1
Comparative Toxicogenomics Database	RT1-DMA
Comparative Toxicogenomics Database	RT1-DMB
Comparative Toxicogenomics Database	RTCA
Comparative Toxicogenomics Database	RTL10
Comparative Toxicogenomics Database	RTN3
Comparative Toxicogenomics Database	RTN4
Comparative Toxicogenomics Database	RUNDC3A
Comparative Toxicogenomics Database	RUNX2
Comparative Toxicogenomics Database	RXRA
Comparative Toxicogenomics Database	RYR2
Comparative Toxicogenomics Database	S100A1
Comparative Toxicogenomics Database	S100A16
Comparative Toxicogenomics Database	S100A4
Comparative Toxicogenomics Database	S100A6
Comparative Toxicogenomics Database	S100A8
Comparative Toxicogenomics Database	S100A9
Comparative Toxicogenomics Database	S100B
Comparative Toxicogenomics Database	S100P
Comparative Toxicogenomics Database	S1PR3
Comparative Toxicogenomics Database	SAA
Comparative Toxicogenomics Database	SAA1
Comparative Toxicogenomics Database	SAA2

Comparative Toxicogenomics Database	SAA3
Comparative Toxicogenomics Database	SAC3D1
Comparative Toxicogenomics Database	SACS
Comparative Toxicogenomics Database	SALL4
Comparative Toxicogenomics Database	SAMD4A
Comparative Toxicogenomics Database	SAMD5
Comparative Toxicogenomics Database	SAR1A
Comparative Toxicogenomics Database	SARM1
Comparative Toxicogenomics Database	SARNP
Comparative Toxicogenomics Database	SARS1
Comparative Toxicogenomics Database	SAT1
Comparative Toxicogenomics Database	SBF1
Comparative Toxicogenomics Database	SBF2
Comparative Toxicogenomics Database	SC5D
Comparative Toxicogenomics Database	SCAF1
Comparative Toxicogenomics Database	SCAF11
Comparative Toxicogenomics Database	SCAMP5
Comparative Toxicogenomics Database	SCAND2P
Comparative Toxicogenomics Database	SCAP
Comparative Toxicogenomics Database	SCARA5
Comparative Toxicogenomics Database	SCARB1
Comparative Toxicogenomics Database	SCARNA12
Comparative Toxicogenomics Database	SCD
Comparative Toxicogenomics Database	SCD1
Comparative Toxicogenomics Database	SCD2
Comparative Toxicogenomics Database	SCML2
Comparative Toxicogenomics Database	SCP2
Comparative Toxicogenomics Database	SCPEP1
Comparative Toxicogenomics Database	SCX
Comparative Toxicogenomics Database	SDC2
Comparative Toxicogenomics Database	SDF2L1
Comparative Toxicogenomics Database	SDHA
Comparative Toxicogenomics Database	SDHB
Comparative Toxicogenomics Database	SDHD
Comparative Toxicogenomics Database	SDR39U1
Comparative Toxicogenomics Database	SDS
Comparative Toxicogenomics Database	SEC11A
Comparative Toxicogenomics Database	SEC11C
Comparative Toxicogenomics Database	SEC14L1
Comparative Toxicogenomics Database	SEC14L3
Comparative Toxicogenomics Database	SEC22B
Comparative Toxicogenomics Database	SEC31A
Comparative Toxicogenomics Database	SEC61G
Comparative Toxicogenomics Database	SEL1L
Comparative Toxicogenomics Database	SELE
Comparative Toxicogenomics Database	SELENBP1
Comparative Toxicogenomics Database	SELENOP
Comparative Toxicogenomics Database	SELENOT
Comparative Toxicogenomics Database	SELL
Comparative Toxicogenomics Database	SELP
Comparative Toxicogenomics Database	SEMA3B
Comparative Toxicogenomics Database	SENP7
Comparative Toxicogenomics Database	SEPHS2
Comparative Toxicogenomics Database	SEPTIN11
Comparative Toxicogenomics Database	SERHL
Comparative Toxicogenomics Database	SERPINA1
Comparative Toxicogenomics Database	SERPINA12
Comparative Toxicogenomics Database	SERPINA1E

Comparative Toxicogenomics Database	SERPINA3C
Comparative Toxicogenomics Database	SERPINA3K
Comparative Toxicogenomics Database	SERPINA6
Comparative Toxicogenomics Database	SERPINA7
Comparative Toxicogenomics Database	SERPINB1A
Comparative Toxicogenomics Database	SERPINB2
Comparative Toxicogenomics Database	SERPINB5
Comparative Toxicogenomics Database	SERPINC1
Comparative Toxicogenomics Database	SERPINE1
Comparative Toxicogenomics Database	SERPINF1
Comparative Toxicogenomics Database	SERPINH1
Comparative Toxicogenomics Database	SESN1
Comparative Toxicogenomics Database	SESN2
Comparative Toxicogenomics Database	SETMAR
Comparative Toxicogenomics Database	SF1
Comparative Toxicogenomics Database	SF3A1
Comparative Toxicogenomics Database	SF3B1
Comparative Toxicogenomics Database	SF3B3
Comparative Toxicogenomics Database	SF3B5
Comparative Toxicogenomics Database	SF3B6
Comparative Toxicogenomics Database	SFPQ
Comparative Toxicogenomics Database	SFR1
Comparative Toxicogenomics Database	SFRP1
Comparative Toxicogenomics Database	SFTPB
Comparative Toxicogenomics Database	SFXN2
Comparative Toxicogenomics Database	SGCB
Comparative Toxicogenomics Database	SGCG
Comparative Toxicogenomics Database	SGK3
Comparative Toxicogenomics Database	SGO1
Comparative Toxicogenomics Database	SGO2
Comparative Toxicogenomics Database	SH3BGRL
Comparative Toxicogenomics Database	SH3BGRL2
Comparative Toxicogenomics Database	SH3BP4
Comparative Toxicogenomics Database	SH3GLB1
Comparative Toxicogenomics Database	SH3PXD2B
Comparative Toxicogenomics Database	SHB
Comparative Toxicogenomics Database	SHC1
Comparative Toxicogenomics Database	SHCBP1
Comparative Toxicogenomics Database	SHH
Comparative Toxicogenomics Database	SHLD2
Comparative Toxicogenomics Database	SHMT1
Comparative Toxicogenomics Database	SHMT2
Comparative Toxicogenomics Database	SIAH1
Comparative Toxicogenomics Database	SIAH2
Comparative Toxicogenomics Database	SIGMAR1
Comparative Toxicogenomics Database	SIL1
Comparative Toxicogenomics Database	SIR-2.1
Comparative Toxicogenomics Database	SIRT1
Comparative Toxicogenomics Database	SIRT2
Comparative Toxicogenomics Database	SIRT3
Comparative Toxicogenomics Database	SIRT4
Comparative Toxicogenomics Database	SIRT5
Comparative Toxicogenomics Database	SIRT6
Comparative Toxicogenomics Database	SKA1
Comparative Toxicogenomics Database	SKA2
Comparative Toxicogenomics Database	SKA3
Comparative Toxicogenomics Database	SKAP2
Comparative Toxicogenomics Database	SKP2

Comparative Toxicogenomics Database	SLA
Comparative Toxicogenomics Database	SLBP
Comparative Toxicogenomics Database	SLC10A2
Comparative Toxicogenomics Database	SLC12A4
Comparative Toxicogenomics Database	SLC12A7
Comparative Toxicogenomics Database	SLC15A1
Comparative Toxicogenomics Database	SLC15A3
Comparative Toxicogenomics Database	SLC16A1
Comparative Toxicogenomics Database	SLC16A12
Comparative Toxicogenomics Database	SLC16A7
Comparative Toxicogenomics Database	SLC17A5
Comparative Toxicogenomics Database	SLC1A1
Comparative Toxicogenomics Database	SLC1A2
Comparative Toxicogenomics Database	SLC1A3
Comparative Toxicogenomics Database	SLC1A4
Comparative Toxicogenomics Database	SLC1A5
Comparative Toxicogenomics Database	SLC20A1
Comparative Toxicogenomics Database	SLC22A1
Comparative Toxicogenomics Database	SLC22A6
Comparative Toxicogenomics Database	SLC22A7
Comparative Toxicogenomics Database	SLC22A8
Comparative Toxicogenomics Database	SLC24A5
Comparative Toxicogenomics Database	SLC25A10
Comparative Toxicogenomics Database	SLC25A11
Comparative Toxicogenomics Database	SLC25A19
Comparative Toxicogenomics Database	SLC25A22
Comparative Toxicogenomics Database	SLC25A37
Comparative Toxicogenomics Database	SLC25A4
Comparative Toxicogenomics Database	SLC25A40
Comparative Toxicogenomics Database	SLC25A5
Comparative Toxicogenomics Database	SLC26A11
Comparative Toxicogenomics Database	SLC26A2
Comparative Toxicogenomics Database	SLC27A2
Comparative Toxicogenomics Database	SLC27A3
Comparative Toxicogenomics Database	SLC27A4
Comparative Toxicogenomics Database	SLC2A1
Comparative Toxicogenomics Database	SLC2A2
Comparative Toxicogenomics Database	SLC2A4
Comparative Toxicogenomics Database	SLC2A9
Comparative Toxicogenomics Database	SLC30A6
Comparative Toxicogenomics Database	SLC31A1
Comparative Toxicogenomics Database	SLC39A8
Comparative Toxicogenomics Database	SLC40A1
Comparative Toxicogenomics Database	SLC41A2
Comparative Toxicogenomics Database	SLC41A3
Comparative Toxicogenomics Database	SLC45A2
Comparative Toxicogenomics Database	SLC4A1
Comparative Toxicogenomics Database	SLC4A4
Comparative Toxicogenomics Database	SLC5A1
Comparative Toxicogenomics Database	SLC5A5
Comparative Toxicogenomics Database	SLC6A14
Comparative Toxicogenomics Database	SLC6A6
Comparative Toxicogenomics Database	SLC7A2
Comparative Toxicogenomics Database	SLC7A5
Comparative Toxicogenomics Database	SLC7A6
Comparative Toxicogenomics Database	SLC7A7
Comparative Toxicogenomics Database	SLC8A1
Comparative Toxicogenomics Database	SLC9A1

Comparative Toxicogenomics Database	SLC9A3
Comparative Toxicogenomics Database	SLC9A3R2
Comparative Toxicogenomics Database	SLCO1A1
Comparative Toxicogenomics Database	SLCO1A4
Comparative Toxicogenomics Database	SLCO2A1
Comparative Toxicogenomics Database	SLF1
Comparative Toxicogenomics Database	SLPR
Comparative Toxicogenomics Database	SMAD1
Comparative Toxicogenomics Database	SMAD2
Comparative Toxicogenomics Database	SMAD3
Comparative Toxicogenomics Database	SMAD4
Comparative Toxicogenomics Database	SMAD5
Comparative Toxicogenomics Database	SMAD7
Comparative Toxicogenomics Database	SMAD9
Comparative Toxicogenomics Database	SMARCA4
Comparative Toxicogenomics Database	SMARCA5
Comparative Toxicogenomics Database	SMARCB1
Comparative Toxicogenomics Database	SMARCE1
Comparative Toxicogenomics Database	SMC1A
Comparative Toxicogenomics Database	SMC2
Comparative Toxicogenomics Database	SMC3
Comparative Toxicogenomics Database	SMC4
Comparative Toxicogenomics Database	SMC6
Comparative Toxicogenomics Database	SMN2
Comparative Toxicogenomics Database	SMNDC1
Comparative Toxicogenomics Database	SMO
Comparative Toxicogenomics Database	SMTN
Comparative Toxicogenomics Database	SMU1
Comparative Toxicogenomics Database	SNAI1
Comparative Toxicogenomics Database	SNAI2
Comparative Toxicogenomics Database	SNAP23
Comparative Toxicogenomics Database	SNCA
Comparative Toxicogenomics Database	SNHG32
Comparative Toxicogenomics Database	SNHG7
Comparative Toxicogenomics Database	SNHG8
Comparative Toxicogenomics Database	SNORA20
Comparative Toxicogenomics Database	SNORA54
Comparative Toxicogenomics Database	SNORA64
Comparative Toxicogenomics Database	SNORA72
Comparative Toxicogenomics Database	NORD103C
Comparative Toxicogenomics Database	SNORD14C
Comparative Toxicogenomics Database	SNORD15A
Comparative Toxicogenomics Database	SNORD22
Comparative Toxicogenomics Database	SNORD46
Comparative Toxicogenomics Database	SNORD56
Comparative Toxicogenomics Database	SNORD59A
Comparative Toxicogenomics Database	SNORD61
Comparative Toxicogenomics Database	SNORD87
Comparative Toxicogenomics Database	SNRNP200
Comparative Toxicogenomics Database	SNRNP25
Comparative Toxicogenomics Database	SNRNP48
Comparative Toxicogenomics Database	SNRPA
Comparative Toxicogenomics Database	SNRPA1
Comparative Toxicogenomics Database	SNRPB2
Comparative Toxicogenomics Database	SNRPD2
Comparative Toxicogenomics Database	SNRPD3
Comparative Toxicogenomics Database	SNRPE
Comparative Toxicogenomics Database	SNX10

Comparative Toxicogenomics Database	SNX18
Comparative Toxicogenomics Database	SNX24
Comparative Toxicogenomics Database	SNX3
Comparative Toxicogenomics Database	SNX4
Comparative Toxicogenomics Database	SNX5
Comparative Toxicogenomics Database	SOCS1
Comparative Toxicogenomics Database	SOCS2
Comparative Toxicogenomics Database	SOCS3
Comparative Toxicogenomics Database	SOD1
Comparative Toxicogenomics Database	SOD2
Comparative Toxicogenomics Database	SOD3
Comparative Toxicogenomics Database	SOD-3
Comparative Toxicogenomics Database	SOS1
Comparative Toxicogenomics Database	SOX13
Comparative Toxicogenomics Database	SOX17
Comparative Toxicogenomics Database	SOX2
Comparative Toxicogenomics Database	SOX3
Comparative Toxicogenomics Database	SOX4
Comparative Toxicogenomics Database	SOX9
Comparative Toxicogenomics Database	SP1
Comparative Toxicogenomics Database	SP100
Comparative Toxicogenomics Database	SP3
Comparative Toxicogenomics Database	SP4
Comparative Toxicogenomics Database	SP7
Comparative Toxicogenomics Database	SPAG1
Comparative Toxicogenomics Database	SPAG5
Comparative Toxicogenomics Database	SPANXD
Comparative Toxicogenomics Database	SPARC
Comparative Toxicogenomics Database	SPATA20
Comparative Toxicogenomics Database	SPATA7
Comparative Toxicogenomics Database	SPC24
Comparative Toxicogenomics Database	SPC25
Comparative Toxicogenomics Database	SPDEF
Comparative Toxicogenomics Database	SPDL1
Comparative Toxicogenomics Database	SPEN
Comparative Toxicogenomics Database	SPHK1
Comparative Toxicogenomics Database	SPI1
Comparative Toxicogenomics Database	SPIN4
Comparative Toxicogenomics Database	SPP1
Comparative Toxicogenomics Database	SPP2
Comparative Toxicogenomics Database	SPRY1
Comparative Toxicogenomics Database	SPRYD4
Comparative Toxicogenomics Database	SPSB3
Comparative Toxicogenomics Database	SPSB4
Comparative Toxicogenomics Database	SPTAN1
Comparative Toxicogenomics Database	SPTBN1
Comparative Toxicogenomics Database	SQLE
Comparative Toxicogenomics Database	SQOR
Comparative Toxicogenomics Database	SQSTM1
Comparative Toxicogenomics Database	SRC
Comparative Toxicogenomics Database	SRD5A2
Comparative Toxicogenomics Database	SRD5A3
Comparative Toxicogenomics Database	SREBF1
Comparative Toxicogenomics Database	SREBF2
Comparative Toxicogenomics Database	SRF
Comparative Toxicogenomics Database	SRGAP3
Comparative Toxicogenomics Database	SRI
Comparative Toxicogenomics Database	SRP19

Comparative Toxicogenomics Database	SRP54A
Comparative Toxicogenomics Database	SRP68
Comparative Toxicogenomics Database	SRP72
Comparative Toxicogenomics Database	SRPK1
Comparative Toxicogenomics Database	SRPRB
Comparative Toxicogenomics Database	SRR
Comparative Toxicogenomics Database	SRRM2
Comparative Toxicogenomics Database	SRRT
Comparative Toxicogenomics Database	SRSF1
Comparative Toxicogenomics Database	SRSF10
Comparative Toxicogenomics Database	SRSF11
Comparative Toxicogenomics Database	SRSF5
Comparative Toxicogenomics Database	SSH1
Comparative Toxicogenomics Database	SSH2
Comparative Toxicogenomics Database	SSRP1
Comparative Toxicogenomics Database	SSTR5-AS1
Comparative Toxicogenomics Database	SSX2IP
Comparative Toxicogenomics Database	ST13
Comparative Toxicogenomics Database	ST3GAL1
Comparative Toxicogenomics Database	ST3GAL3
Comparative Toxicogenomics Database	ST8SIA4
Comparative Toxicogenomics Database	STAMBPL1
Comparative Toxicogenomics Database	STAR
Comparative Toxicogenomics Database	STARD3
Comparative Toxicogenomics Database	STARD4
Comparative Toxicogenomics Database	STAT1
Comparative Toxicogenomics Database	STAT3
Comparative Toxicogenomics Database	STAT4
Comparative Toxicogenomics Database	STAT5A
Comparative Toxicogenomics Database	STAT5B
Comparative Toxicogenomics Database	STAT6
Comparative Toxicogenomics Database	STBD1
Comparative Toxicogenomics Database	STEAP4
Comparative Toxicogenomics Database	STIL
Comparative Toxicogenomics Database	STIP1
Comparative Toxicogenomics Database	STK11
Comparative Toxicogenomics Database	STK17A
Comparative Toxicogenomics Database	STK17B
Comparative Toxicogenomics Database	STK38L
Comparative Toxicogenomics Database	STK39
Comparative Toxicogenomics Database	STMN1
Comparative Toxicogenomics Database	STOM
Comparative Toxicogenomics Database	STOML2
Comparative Toxicogenomics Database	STRBP
Comparative Toxicogenomics Database	STRIP1
Comparative Toxicogenomics Database	STRN3
Comparative Toxicogenomics Database	STT3A
Comparative Toxicogenomics Database	STT3B
Comparative Toxicogenomics Database	STX4
Comparative Toxicogenomics Database	STX8
Comparative Toxicogenomics Database	SUCLA2
Comparative Toxicogenomics Database	SUCLG1
Comparative Toxicogenomics Database	SUCLG2
Comparative Toxicogenomics Database	SULF1
Comparative Toxicogenomics Database	SULF2
Comparative Toxicogenomics Database	SULT1A1
Comparative Toxicogenomics Database	SULT1A2
Comparative Toxicogenomics Database	SULT1A3

Comparative Toxicogenomics Database	SULT1C2
Comparative Toxicogenomics Database	SULT1E1
Comparative Toxicogenomics Database	SULT2A8
Comparative Toxicogenomics Database	SULT4A1
Comparative Toxicogenomics Database	SUMO1
Comparative Toxicogenomics Database	SURF1
Comparative Toxicogenomics Database	SURF4
Comparative Toxicogenomics Database	SUSD1
Comparative Toxicogenomics Database	SUV39H1
Comparative Toxicogenomics Database	SUV39H2
Comparative Toxicogenomics Database	SUZ12
Comparative Toxicogenomics Database	SYAP1
Comparative Toxicogenomics Database	SYBU
Comparative Toxicogenomics Database	SYK
Comparative Toxicogenomics Database	SYNE1
Comparative Toxicogenomics Database	SYNE3
Comparative Toxicogenomics Database	SYNE4
Comparative Toxicogenomics Database	SYNGR3
Comparative Toxicogenomics Database	NJ2BP-COX16
Comparative Toxicogenomics Database	SYP
Comparative Toxicogenomics Database	SYTL2
Comparative Toxicogenomics Database	SYTL4
Comparative Toxicogenomics Database	SYTL5
Comparative Toxicogenomics Database	SYVN1
Comparative Toxicogenomics Database	T
Comparative Toxicogenomics Database	TACC3
Comparative Toxicogenomics Database	TACO1
Comparative Toxicogenomics Database	TAF1
Comparative Toxicogenomics Database	TAF15
Comparative Toxicogenomics Database	TAF5
Comparative Toxicogenomics Database	TAGLN
Comparative Toxicogenomics Database	TAGLN2
Comparative Toxicogenomics Database	TAK1
Comparative Toxicogenomics Database	TALDO1
Comparative Toxicogenomics Database	TAP1
Comparative Toxicogenomics Database	TAP2
Comparative Toxicogenomics Database	TAPBP
Comparative Toxicogenomics Database	TAX1BP3
Comparative Toxicogenomics Database	TBATA
Comparative Toxicogenomics Database	TBC1D2
Comparative Toxicogenomics Database	TBC1D5
Comparative Toxicogenomics Database	TBC1D7
Comparative Toxicogenomics Database	TBCA
Comparative Toxicogenomics Database	TBCCD1
Comparative Toxicogenomics Database	TBK1
Comparative Toxicogenomics Database	TBL1XR1
Comparative Toxicogenomics Database	TBX1
Comparative Toxicogenomics Database	TBX21
Comparative Toxicogenomics Database	TBX6
Comparative Toxicogenomics Database	TCF12
Comparative Toxicogenomics Database	TCF19
Comparative Toxicogenomics Database	TCF4
Comparative Toxicogenomics Database	TCF7
Comparative Toxicogenomics Database	TCF7L1
Comparative Toxicogenomics Database	TCF7L2
Comparative Toxicogenomics Database	TCOF1
Comparative Toxicogenomics Database	TCP11L2
Comparative Toxicogenomics Database	TCRA

Comparative Toxicogenomics Database	TCRB
Comparative Toxicogenomics Database	TDO2
Comparative Toxicogenomics Database	TDP1
Comparative Toxicogenomics Database	TDP2
Comparative Toxicogenomics Database	TECR
Comparative Toxicogenomics Database	TENT2
Comparative Toxicogenomics Database	TENT5C
Comparative Toxicogenomics Database	TERF1
Comparative Toxicogenomics Database	TERT
Comparative Toxicogenomics Database	TES
Comparative Toxicogenomics Database	TEX264
Comparative Toxicogenomics Database	TEX30
Comparative Toxicogenomics Database	TF
Comparative Toxicogenomics Database	TFAM
Comparative Toxicogenomics Database	TFAP2A
Comparative Toxicogenomics Database	TFAP2C
Comparative Toxicogenomics Database	TFAP4
Comparative Toxicogenomics Database	TFB1M
Comparative Toxicogenomics Database	TFDP1
Comparative Toxicogenomics Database	TFF1
Comparative Toxicogenomics Database	TFF3
Comparative Toxicogenomics Database	TFPI
Comparative Toxicogenomics Database	TFPI2
Comparative Toxicogenomics Database	TFRC
Comparative Toxicogenomics Database	TG
Comparative Toxicogenomics Database	TGFA
Comparative Toxicogenomics Database	TGFB1
Comparative Toxicogenomics Database	TGFB2
Comparative Toxicogenomics Database	TGFB3
Comparative Toxicogenomics Database	TGFBI
Comparative Toxicogenomics Database	TGFBR1
Comparative Toxicogenomics Database	TGFBR2
Comparative Toxicogenomics Database	TGFBR3
Comparative Toxicogenomics Database	TGIF1
Comparative Toxicogenomics Database	TGIF2
Comparative Toxicogenomics Database	TGM1
Comparative Toxicogenomics Database	TGM3
Comparative Toxicogenomics Database	TGM5
Comparative Toxicogenomics Database	TH
Comparative Toxicogenomics Database	THBD
Comparative Toxicogenomics Database	THBS1
Comparative Toxicogenomics Database	THOC1
Comparative Toxicogenomics Database	THPO
Comparative Toxicogenomics Database	THRA
Comparative Toxicogenomics Database	THRAP3
Comparative Toxicogenomics Database	THRB
Comparative Toxicogenomics Database	THRSP
Comparative Toxicogenomics Database	THSD4
Comparative Toxicogenomics Database	THY1
Comparative Toxicogenomics Database	TICAM1
Comparative Toxicogenomics Database	TIFA
Comparative Toxicogenomics Database	TIGAR
Comparative Toxicogenomics Database	TIMELESS
Comparative Toxicogenomics Database	TIMM10
Comparative Toxicogenomics Database	TIMM13
Comparative Toxicogenomics Database	TIMM22
Comparative Toxicogenomics Database	TIMM23
Comparative Toxicogenomics Database	TIMM44

Comparative Toxicogenomics Database	TIMM8A
Comparative Toxicogenomics Database	TIMM8B
Comparative Toxicogenomics Database	TIMP1
Comparative Toxicogenomics Database	TIMP2
Comparative Toxicogenomics Database	TIMP3
Comparative Toxicogenomics Database	TIMP4
Comparative Toxicogenomics Database	TINF2
Comparative Toxicogenomics Database	TIPIN
Comparative Toxicogenomics Database	TJP1
Comparative Toxicogenomics Database	TJP3
Comparative Toxicogenomics Database	TK1
Comparative Toxicogenomics Database	TKT
Comparative Toxicogenomics Database	TLCD3A
Comparative Toxicogenomics Database	TLK1
Comparative Toxicogenomics Database	TLK2
Comparative Toxicogenomics Database	TLR1
Comparative Toxicogenomics Database	TLR2
Comparative Toxicogenomics Database	TLR3
Comparative Toxicogenomics Database	TLR4
Comparative Toxicogenomics Database	TLR5
Comparative Toxicogenomics Database	TLR8
Comparative Toxicogenomics Database	TM4SF5
Comparative Toxicogenomics Database	TM6SF1
Comparative Toxicogenomics Database	TMC4
Comparative Toxicogenomics Database	TMED10
Comparative Toxicogenomics Database	TMED3
Comparative Toxicogenomics Database	TMED4
Comparative Toxicogenomics Database	TMED5
Comparative Toxicogenomics Database	TMEM106C
Comparative Toxicogenomics Database	TMEM107
Comparative Toxicogenomics Database	TMEM109
Comparative Toxicogenomics Database	TMEM11
Comparative Toxicogenomics Database	TMEM117
Comparative Toxicogenomics Database	TMEM135
Comparative Toxicogenomics Database	TMEM164
Comparative Toxicogenomics Database	TMEM181
Comparative Toxicogenomics Database	TMEM199
Comparative Toxicogenomics Database	TMEM237
Comparative Toxicogenomics Database	TMEM26
Comparative Toxicogenomics Database	TMEM263
Comparative Toxicogenomics Database	TMEM33
Comparative Toxicogenomics Database	TMEM38B
Comparative Toxicogenomics Database	TMEM40
Comparative Toxicogenomics Database	TMEM41B
Comparative Toxicogenomics Database	TMEM47
Comparative Toxicogenomics Database	TMEM64
Comparative Toxicogenomics Database	TMEM70
Comparative Toxicogenomics Database	TMEM97
Comparative Toxicogenomics Database	TMPO
Comparative Toxicogenomics Database	TMPRSS2
Comparative Toxicogenomics Database	TMPRSS3
Comparative Toxicogenomics Database	TMSB10
Comparative Toxicogenomics Database	TMSB15A
Comparative Toxicogenomics Database	TMSB4X
Comparative Toxicogenomics Database	TMSB4Y
Comparative Toxicogenomics Database	TMTC2
Comparative Toxicogenomics Database	TMX1
Comparative Toxicogenomics Database	TMX4

Comparative Toxicogenomics Database	TNF
Comparative Toxicogenomics Database	TNFAIP3
Comparative Toxicogenomics Database	TNFAIP6
Comparative Toxicogenomics Database	TNFAIP8L2
Comparative Toxicogenomics Database	TNFRSF10A
Comparative Toxicogenomics Database	TNFRSF10B
Comparative Toxicogenomics Database	TNFRSF10C
Comparative Toxicogenomics Database	TNFRSF10D
Comparative Toxicogenomics Database	TNFRSF11A
Comparative Toxicogenomics Database	TNFRSF11B
Comparative Toxicogenomics Database	TNFRSF1A
Comparative Toxicogenomics Database	TNFRSF1B
Comparative Toxicogenomics Database	TNFRSF21
Comparative Toxicogenomics Database	TNFRSF25
Comparative Toxicogenomics Database	TNFRSF9
Comparative Toxicogenomics Database	TNFSF10
Comparative Toxicogenomics Database	TNFSF11
Comparative Toxicogenomics Database	TNFSF14
Comparative Toxicogenomics Database	TNIP1
Comparative Toxicogenomics Database	TNK2
Comparative Toxicogenomics Database	TNMD
Comparative Toxicogenomics Database	TNNC1
Comparative Toxicogenomics Database	TNNT2
Comparative Toxicogenomics Database	TNNT3
Comparative Toxicogenomics Database	TNPO1
Comparative Toxicogenomics Database	TNS1
Comparative Toxicogenomics Database	TOB2
Comparative Toxicogenomics Database	TOMM22
Comparative Toxicogenomics Database	TOMM34
Comparative Toxicogenomics Database	TOMM7
Comparative Toxicogenomics Database	TOMM70A
Comparative Toxicogenomics Database	TOP1
Comparative Toxicogenomics Database	TOP2A
Comparative Toxicogenomics Database	TOP2B
Comparative Toxicogenomics Database	TOPBP1
Comparative Toxicogenomics Database	TOR1B
Comparative Toxicogenomics Database	TP53
Comparative Toxicogenomics Database	TP53BP2
Comparative Toxicogenomics Database	TP53I3
Comparative Toxicogenomics Database	TP53INP1
Comparative Toxicogenomics Database	TP63
Comparative Toxicogenomics Database	TP73
Comparative Toxicogenomics Database	TPD52L1
Comparative Toxicogenomics Database	TPI1
Comparative Toxicogenomics Database	TPM2
Comparative Toxicogenomics Database	TPM3
Comparative Toxicogenomics Database	TPM4
Comparative Toxicogenomics Database	TPMT
Comparative Toxicogenomics Database	TPO
Comparative Toxicogenomics Database	TPR
Comparative Toxicogenomics Database	TPRG1L
Comparative Toxicogenomics Database	TPT1
Comparative Toxicogenomics Database	TPTE
Comparative Toxicogenomics Database	TPX2
Comparative Toxicogenomics Database	TRA2A
Comparative Toxicogenomics Database	TRA2B
Comparative Toxicogenomics Database	TRABD
Comparative Toxicogenomics Database	TRABD2A

Comparative Toxicogenomics Database	TRAC
Comparative Toxicogenomics Database	TRAF1
Comparative Toxicogenomics Database	TRAF2
Comparative Toxicogenomics Database	TRAF6
Comparative Toxicogenomics Database	TRAM1
Comparative Toxicogenomics Database	TRAP1
Comparative Toxicogenomics Database	TRAPPC3
Comparative Toxicogenomics Database	TRAPPC5
Comparative Toxicogenomics Database	TRAPPC6A
Comparative Toxicogenomics Database	TRERF1
Comparative Toxicogenomics Database	TRGV9
Comparative Toxicogenomics Database	TRH
Comparative Toxicogenomics Database	TRIM13
Comparative Toxicogenomics Database	TRIM28
Comparative Toxicogenomics Database	TRIM59
Comparative Toxicogenomics Database	TRIM63
Comparative Toxicogenomics Database	TRIO
Comparative Toxicogenomics Database	TRIP13
Comparative Toxicogenomics Database	TRMT6
Comparative Toxicogenomics Database	TROAP
Comparative Toxicogenomics Database	TRP53
Comparative Toxicogenomics Database	TRPC5
Comparative Toxicogenomics Database	TRPC6
Comparative Toxicogenomics Database	TSC1
Comparative Toxicogenomics Database	TSC2
Comparative Toxicogenomics Database	TSC22D1
Comparative Toxicogenomics Database	TSC22D2
Comparative Toxicogenomics Database	TSC22D3
Comparative Toxicogenomics Database	TSEN15
Comparative Toxicogenomics Database	TSFM
Comparative Toxicogenomics Database	TSG101
Comparative Toxicogenomics Database	TSHB
Comparative Toxicogenomics Database	TSHR
Comparative Toxicogenomics Database	TSHZ1
Comparative Toxicogenomics Database	TSN
Comparative Toxicogenomics Database	TSPAN1
Comparative Toxicogenomics Database	TSPAN12
Comparative Toxicogenomics Database	TSPAN31
Comparative Toxicogenomics Database	TSPAN8
Comparative Toxicogenomics Database	TSPO
Comparative Toxicogenomics Database	TSPYL1
Comparative Toxicogenomics Database	TST
Comparative Toxicogenomics Database	TSTD1
Comparative Toxicogenomics Database	TTC9
Comparative Toxicogenomics Database	TTC9C
Comparative Toxicogenomics Database	TTF2
Comparative Toxicogenomics Database	TTK
Comparative Toxicogenomics Database	TTN
Comparative Toxicogenomics Database	TUB
Comparative Toxicogenomics Database	TUBA1A
Comparative Toxicogenomics Database	TUBA1C
Comparative Toxicogenomics Database	TUBA4A
Comparative Toxicogenomics Database	TUBB4B
Comparative Toxicogenomics Database	TUBB5
Comparative Toxicogenomics Database	TUBB6
Comparative Toxicogenomics Database	TUBG1
Comparative Toxicogenomics Database	TUBGCP4
Comparative Toxicogenomics Database	TUFM

Comparative Toxicogenomics Database	TUT4
Comparative Toxicogenomics Database	TWF2
Comparative Toxicogenomics Database	TWIST1
Comparative Toxicogenomics Database	TWIST2
Comparative Toxicogenomics Database	TXN
Comparative Toxicogenomics Database	TXN2
Comparative Toxicogenomics Database	TXNIP
Comparative Toxicogenomics Database	TXNL1
Comparative Toxicogenomics Database	TXNL4B
Comparative Toxicogenomics Database	TXNRD1
Comparative Toxicogenomics Database	TYMS
Comparative Toxicogenomics Database	TYR
Comparative Toxicogenomics Database	TYROBP
Comparative Toxicogenomics Database	TYRP1
Comparative Toxicogenomics Database	UACA
Comparative Toxicogenomics Database	UAP1
Comparative Toxicogenomics Database	UBA7
Comparative Toxicogenomics Database	UBB
Comparative Toxicogenomics Database	UBC
Comparative Toxicogenomics Database	UBE2C
Comparative Toxicogenomics Database	UBE2D3
Comparative Toxicogenomics Database	UBE2G2
Comparative Toxicogenomics Database	UBE2H
Comparative Toxicogenomics Database	UBE2L3
Comparative Toxicogenomics Database	UBE2QL1
Comparative Toxicogenomics Database	UBE2T
Comparative Toxicogenomics Database	UBE2U
Comparative Toxicogenomics Database	UBE2V1
Comparative Toxicogenomics Database	UBE3A
Comparative Toxicogenomics Database	UBE4A
Comparative Toxicogenomics Database	UCK2
Comparative Toxicogenomics Database	UCP1
Comparative Toxicogenomics Database	UCP2
Comparative Toxicogenomics Database	UCP3
Comparative Toxicogenomics Database	UGCG
Comparative Toxicogenomics Database	UGP2
Comparative Toxicogenomics Database	UGT1A1
Comparative Toxicogenomics Database	UGT1A10
Comparative Toxicogenomics Database	UGT1A3
Comparative Toxicogenomics Database	UGT1A7
Comparative Toxicogenomics Database	UGT1A8
Comparative Toxicogenomics Database	UGT1A9
Comparative Toxicogenomics Database	UGT2B11
Comparative Toxicogenomics Database	UGT2B15
Comparative Toxicogenomics Database	UGT2B5
Comparative Toxicogenomics Database	UGT2B7
Comparative Toxicogenomics Database	UHRF1
Comparative Toxicogenomics Database	ULBP1
Comparative Toxicogenomics Database	ULBP2
Comparative Toxicogenomics Database	ULBP3
Comparative Toxicogenomics Database	ULE-3
Comparative Toxicogenomics Database	UNC119B
Comparative Toxicogenomics Database	UNG
Comparative Toxicogenomics Database	UPK1A
Comparative Toxicogenomics Database	UPP1
Comparative Toxicogenomics Database	UQCR
Comparative Toxicogenomics Database	UQCR10
Comparative Toxicogenomics Database	UQCRB

Comparative Toxicogenomics Database	UQCRC1
Comparative Toxicogenomics Database	UQCRC2
Comparative Toxicogenomics Database	UQCRH
Comparative Toxicogenomics Database	UROS
Comparative Toxicogenomics Database	USE1
Comparative Toxicogenomics Database	USH2A
Comparative Toxicogenomics Database	USP1
Comparative Toxicogenomics Database	USP10
Comparative Toxicogenomics Database	USP18
Comparative Toxicogenomics Database	USP2
Comparative Toxicogenomics Database	USP36
Comparative Toxicogenomics Database	USP4
Comparative Toxicogenomics Database	USP47
Comparative Toxicogenomics Database	USP7
Comparative Toxicogenomics Database	UST
Comparative Toxicogenomics Database	UTP11
Comparative Toxicogenomics Database	UTRN
Comparative Toxicogenomics Database	VAMP2
Comparative Toxicogenomics Database	VAPA
Comparative Toxicogenomics Database	VASP
Comparative Toxicogenomics Database	VAV1
Comparative Toxicogenomics Database	VAV3
Comparative Toxicogenomics Database	VCAM1
Comparative Toxicogenomics Database	VCL
Comparative Toxicogenomics Database	VCPKMT
Comparative Toxicogenomics Database	VDAC1
Comparative Toxicogenomics Database	VDAC3
Comparative Toxicogenomics Database	VDR
Comparative Toxicogenomics Database	VEGFA
Comparative Toxicogenomics Database	VEGFB
Comparative Toxicogenomics Database	VEGFC
Comparative Toxicogenomics Database	VIM
Comparative Toxicogenomics Database	VKORC1
Comparative Toxicogenomics Database	VNN1
Comparative Toxicogenomics Database	VPS13B
Comparative Toxicogenomics Database	VPS25
Comparative Toxicogenomics Database	VPS26C
Comparative Toxicogenomics Database	VPS29
Comparative Toxicogenomics Database	VRK1
Comparative Toxicogenomics Database	VSIG2
Comparative Toxicogenomics Database	VTG2
Comparative Toxicogenomics Database	VWF
Comparative Toxicogenomics Database	VXN
Comparative Toxicogenomics Database	WARS
Comparative Toxicogenomics Database	WARS1
Comparative Toxicogenomics Database	WAS
Comparative Toxicogenomics Database	WASF2
Comparative Toxicogenomics Database	WASL
Comparative Toxicogenomics Database	WBP11
Comparative Toxicogenomics Database	WDHD1
Comparative Toxicogenomics Database	WDR43
Comparative Toxicogenomics Database	WDR5
Comparative Toxicogenomics Database	WEE1
Comparative Toxicogenomics Database	WFDC2
Comparative Toxicogenomics Database	WFDC21
Comparative Toxicogenomics Database	WFS1
Comparative Toxicogenomics Database	WNK2
Comparative Toxicogenomics Database	WNT1

Comparative Toxicogenomics Database	WNT10A
Comparative Toxicogenomics Database	WNT10B
Comparative Toxicogenomics Database	WNT2
Comparative Toxicogenomics Database	WNT3A
Comparative Toxicogenomics Database	WNT5A
Comparative Toxicogenomics Database	WNT5B
Comparative Toxicogenomics Database	WRN
Comparative Toxicogenomics Database	WSB1
Comparative Toxicogenomics Database	WWC1
Comparative Toxicogenomics Database	WWP2
Comparative Toxicogenomics Database	XAB2
Comparative Toxicogenomics Database	XBP1
Comparative Toxicogenomics Database	XDH
Comparative Toxicogenomics Database	XIAP
Comparative Toxicogenomics Database	XPO1
Comparative Toxicogenomics Database	XPO5
Comparative Toxicogenomics Database	XRCC1
Comparative Toxicogenomics Database	XRCC2
Comparative Toxicogenomics Database	YARS
Comparative Toxicogenomics Database	YARS1
Comparative Toxicogenomics Database	YARS2
Comparative Toxicogenomics Database	YBX1
Comparative Toxicogenomics Database	YBX3
Comparative Toxicogenomics Database	YIF1A
Comparative Toxicogenomics Database	YIF1B
Comparative Toxicogenomics Database	YIPF5
Comparative Toxicogenomics Database	YPEL2
Comparative Toxicogenomics Database	YPEL5
Comparative Toxicogenomics Database	YWHAB
Comparative Toxicogenomics Database	YWHAE
Comparative Toxicogenomics Database	YWHAG
Comparative Toxicogenomics Database	YWHAH
Comparative Toxicogenomics Database	YWHAQ
Comparative Toxicogenomics Database	YWHAZ
Comparative Toxicogenomics Database	ZBTB10
Comparative Toxicogenomics Database	ZBTB16
Comparative Toxicogenomics Database	ZBTB2
Comparative Toxicogenomics Database	ZBTB7A
Comparative Toxicogenomics Database	ZC2HC1A
Comparative Toxicogenomics Database	ZC3H14
Comparative Toxicogenomics Database	ZC3HAV1
Comparative Toxicogenomics Database	ZCCHC7
Comparative Toxicogenomics Database	ZDHHC2
Comparative Toxicogenomics Database	ZDHHC23
Comparative Toxicogenomics Database	ZDHHC7
Comparative Toxicogenomics Database	ZEB1
Comparative Toxicogenomics Database	ZEB2
Comparative Toxicogenomics Database	ZFAND5
Comparative Toxicogenomics Database	ZFP148
Comparative Toxicogenomics Database	ZFP207
Comparative Toxicogenomics Database	ZFP354A
Comparative Toxicogenomics Database	ZFP36
Comparative Toxicogenomics Database	ZFP62
Comparative Toxicogenomics Database	ZFP740
Comparative Toxicogenomics Database	ZFP9
Comparative Toxicogenomics Database	ZFP91
Comparative Toxicogenomics Database	ZFR
Comparative Toxicogenomics Database	ZHX2

Comparative Toxicogenomics Database	ZKSCAN1
Comparative Toxicogenomics Database	ZKSCAN2
Comparative Toxicogenomics Database	ZKSCAN3
Comparative Toxicogenomics Database	ZMPSTE24
Comparative Toxicogenomics Database	ZMYM2
Comparative Toxicogenomics Database	ZMYM3
Comparative Toxicogenomics Database	ZNF135
Comparative Toxicogenomics Database	ZNF148
Comparative Toxicogenomics Database	ZNF292
Comparative Toxicogenomics Database	ZNF33B
Comparative Toxicogenomics Database	ZNF367
Comparative Toxicogenomics Database	ZNF385B
Comparative Toxicogenomics Database	ZNF462
Comparative Toxicogenomics Database	ZNF467
Comparative Toxicogenomics Database	ZNF507
Comparative Toxicogenomics Database	ZNF6
Comparative Toxicogenomics Database	ZNF703
Comparative Toxicogenomics Database	ZNF84
Comparative Toxicogenomics Database	ZNF91
Comparative Toxicogenomics Database	ZNRD1
Comparative Toxicogenomics Database	ZNRF2
Comparative Toxicogenomics Database	ZSWIM6
Comparative Toxicogenomics Database	ZSWIM8
Comparative Toxicogenomics Database	ZWILCH
Comparative Toxicogenomics Database	ZWINT
TargetNet	ESR1
TargetNet	PTGS2
TargetNet	CYP1A2
TargetNet	STS
TargetNet	AKR1B1
TargetNet	TUBB2B
TargetNet	ALOX5
TargetNet	DUSP3
TargetNet	RELA
TargetNet	CYP19A1
TargetNet	PTGS2
TargetNet	CA5A
TargetNet	ESR2
TargetNet	XDH
TargetNet	APP
TargetNet	MAOB
TargetNet	Maob
TargetNet	CA12
TargetNet	CA14
TargetNet	MAOA
TargetNet	Maoa
TargetNet	PTGS1
TargetNet	APOBEC3G
TargetNet	PTGS1
TargetNet	CA9
TargetNet	MIF
TargetNet	PPO2
TargetNet	ache
TargetNet	ALOX15
TargetNet	CTDSP1
TargetNet	AHR
TargetNet	APOBEC3A
TargetNet	HNF4A

TargetNet	HDAC4
TargetNet	CASP9
TargetNet	CES2
TargetNet	MMP9
TargetNet	Tubb2b
TargetNet	BCL2A1
TargetNet	ABCG2
TargetNet	CA7
TargetNet	GPR35
TargetNet	HSD17B1
TargetNet	MGLL
TargetNet	TUBA1A
TargetNet	Alox5
TargetNet	RIPK2
TargetNet	Akr1b1
TargetNet	Ptgs2
TargetNet	CA13
TargetNet	Dyrk1a
TargetNet	CA5B
TargetNet	DNMT1
TargetNet	CA4
TargetNet	CA6
TargetNet	TLR9
TargetNet	HSD17B2
TargetNet	HSD17B3
TargetNet	PRKCE
TargetNet	Grin2b
TargetNet	Htr6
TargetNet	ATP4A
TargetNet	GRM4
TargetNet	CDC25B
TargetNet	PIM1
TargetNet	GRIN2B
TargetNet	HCAR2
TargetNet	Ar
TargetNet	PLIN5
TargetNet	NR2F2
TargetNet	PLIN1
TargetNet	PRKCD
TargetNet	CES1
TargetNet	ALPL
TargetNet	Grin1
TargetNet	PRKCG
TargetNet	PLA2G1B
TargetNet	AKR1B1
TargetNet	CA1
TargetNet	HDAC6
TargetNet	Drd1
TargetNet	Nos2
TargetNet	lef
TargetNet	PRKACA
TargetNet	Rac1
TargetNet	CA2
TargetNet	MCL1
TargetNet	S1PR4
TargetNet	PTPN1
TargetNet	CACNA1B
TargetNet	HDAC8

TargetNet	ampC
TargetNet	DRD1
TargetNet	ADRA2C
TargetNet	Slc6a3
TargetNet	Chrna7
TargetNet	METAP2
TargetNet	PTPN7
TargetNet	RORA
TargetNet	RARA
TargetNet	TAAR1
TargetNet	HTR5A
TargetNet	Taar1
TargetNet	GALR3
TargetNet	DRD5
TargetNet	SIGMAR1
TargetNet	PLAU
TargetNet	RPS6KA3
TargetNet	HDAC2
TargetNet	RET
TargetNet	HTR1E
TargetNet	Pdgfrb
TargetNet	ACHE
TargetNet	RARG
TargetNet	CSNK2A1
TargetNet	S1PR1
TargetNet	Cnr2
TargetNet	[illegible]HRFAM7A
TargetNet	RARB
TargetNet	S1PR2
TargetNet	Chrm2
TargetNet	Adra2c
TargetNet	CLK1
TargetNet	EGFR
TargetNet	CHRM4
TargetNet	PTGER2
TargetNet	FYN
TargetNet	HTR2C
TargetNet	NR1H3
TargetNet	AKR1C3
TargetNet	PRSS2
TargetNet	DYRK1A
TargetNet	Oprk1
TargetNet	MDM2
TargetNet	CYP2C19
TargetNet	CHRNA7
TargetNet	ABCB1
TargetNet	BCHE
TargetNet	ache
TargetNet	EGFR
TargetNet	CACNA1H
TargetNet	RAC1
TargetNet	PTPN22
TargetNet	ADRA2A
TargetNet	PTPRC
TargetNet	TNF
TargetNet	PTGES
TargetNet	PDGFRA
TargetNet	NR1H4

TargetNet	HSP90AA1
TargetNet	CDK5
TargetNet	Htr2c
TargetNet	CYP2C9
TargetNet	SERPINE1
TargetNet	TERT
TargetNet	AR
TargetNet	SRC
TargetNet	PRKCB
TargetNet	THRA
TargetNet	Sigmar1
TargetNet	RAF1
TargetNet	OPRK1
TargetNet	MMP12
TargetNet	PTK2
TargetNet	CYP11B2
TargetNet	DHODH
TargetNet	CYP17A1
TargetNet	GSK3B
TargetNet	CDK1
TargetNet	FLT3
TargetNet	CHRM1
TargetNet	CHRNA4
TargetNet	OPRK1
TargetNet	OPRD1
TargetNet	PDGFRB
TargetNet	SYK
TargetNet	MAPKAPK2
TargetNet	DRD2
TargetNet	CHRM5
TargetNet	NOS3
TargetNet	GSK3A
TargetNet	HDAC3
TargetNet	SLC6A3
TargetNet	Chrna4
TargetNet	HDAC1
TargetNet	Oprm1
TargetNet	GRIA2
TargetNet	CDC42
TargetNet	MMP2
TargetNet	Adora2a
TargetNet	Drd4
TargetNet	ACE
TargetNet	PLAT
TargetNet	ICAM1
TargetNet	HDAC1
TargetNet	S1PR5
TargetNet	CYSLTR1
TargetNet	ANPEP
TargetNet	Ache
TargetNet	OPRM1
TargetNet	PDE4D
TargetNet	AKT1
TargetNet	CRHR1
TargetNet	SRD5A2
TargetNet	PTGER1
TargetNet	MME
TargetNet	Cnr1

TargetNet	SCN5A
TargetNet	ADRA1A
TargetNet	HCRTR2
TargetNet	Trpv1
TargetNet	CCR3
TargetNet	CTSB
TargetNet	PTGDR2
TargetNet	P2RY12
TargetNet	WEE1
TargetNet	Agtr1b
TargetNet	ADORA3
TargetNet	TMIGD3
TargetNet	AGTR1
TargetNet	PTAFR
TargetNet	Tacr1
TargetNet	ADRA1D
TargetNet	GRM2
TargetNet	ACACA
TargetNet	THRB
TargetNet	ADA
TargetNet	VCAM1
TargetNet	SIRT1
TargetNet	TNK2
TargetNet	Htr1a
TargetNet	SCD
TargetNet	MLNR
TargetNet	ACACB
TargetNet	PDE2A
TargetNet	ALK
TargetNet	Mc1r
TargetNet	MC1R
TargetNet	ITGB5
TargetNet	HSD11B2
TargetNet	NPY1R
TargetNet	ADRA2B
TargetNet	ADRA1B
TargetNet	HRH1
TargetNet	LTB4R
TargetNet	Ednrb
TargetNet	TACR2
TargetNet	Fnta
TargetNet	IMPDH2
TargetNet	AGTR2
TargetNet	Adra1a
TargetNet	MTNR1B
TargetNet	BMP1
TargetNet	HMGCR
TargetNet	HTR3A
TargetNet	PGR
TargetNet	Htr3a
TargetNet	ADAM10
TargetNet	PIM2
TargetNet	ACHE
TargetNet	GHSR
TargetNet	FDFT1
TargetNet	LTA4H
TargetNet	ADORA1
TargetNet	Adra1d

TargetNet	SLC5A2
TargetNet	CTSS
TargetNet	LRRK2
TargetNet	CNR2
TargetNet	AVPR1A
TargetNet	PRKCQ
TargetNet	JAK1
TargetNet	CXCR2
TargetNet	CXCR1
TargetNet	MC3R
TargetNet	HRH2
TargetNet	ACE
TargetNet	PTGIR
TargetNet	MAP2K1
TargetNet	Ptger4
TargetNet	JAK3
TargetNet	ERBB2
TargetNet	CDK4
TargetNet	NTRK1
TargetNet	EPHB4
TargetNet	ITGB3
TargetNet	PDE7A
TargetNet	PTPN2
TargetNet	CCR4
TargetNet	BACE1
TargetNet	KCNH2
TargetNet	CACNA1G
TargetNet	Adora1
TargetNet	Hmgcr
TargetNet	FGFR1
TargetNet	OPRL1
TargetNet	NPY5R
TargetNet	SIRT2
TargetNet	MC5R
TargetNet	DRD4
TargetNet	CCNA1
TargetNet	Oprm1
TargetNet	PTGFR
TargetNet	PREP
TargetNet	NPBWR1
TargetNet	Dpp4
TargetNet	TGM2
TargetNet	SOAT1
TargetNet	MTOR
TargetNet	AKT3
TargetNet	Avpr2
TargetNet	IGF1R
TargetNet	Grm1
TargetNet	Crhr1
TargetNet	ICMT
TargetNet	KCNA5
TargetNet	GRB2
TargetNet	PIK3CG
TargetNet	DHFR
TargetNet	Dhfr
TargetNet	RXRB
TargetNet	ADRB1
TargetNet	ZAP70

TargetNet	MET
TargetNet	Dhfr
TargetNet	CTSL
TargetNet	PPARD
TargetNet	MPL
TargetNet	EPHX2
TargetNet	BTK
TargetNet	HTR4
TargetNet	TGFBR1
TargetNet	TRPV1
TargetNet	AVPR2
TargetNet	TACR1
TargetNet	MCHR1
TargetNet	Ptger2
TargetNet	CTSG
TargetNet	BACE2
TargetNet	CA4
TargetNet	EDNRA
TargetNet	PTGDR
TargetNet	TACR3
TargetNet	F2R
TargetNet	Ednra
TargetNet	HTR1F
TargetNet	HCRTR1
TargetNet	PSEN2
TargetNet	ADORA2A
TargetNet	ADORA2B
TargetNet	Cnr2
TargetNet	CASR
TargetNet	Hrh1
TargetNet	SSTR4
TargetNet	CASP8
TargetNet	Fdft1
TargetNet	P2RY6
TargetNet	ROCK1
TargetNet	TEK
TargetNet	CDK5R1
TargetNet	CDK2
TargetNet	FNTB
TargetNet	Htr4
TargetNet	FNTB
TargetNet	Nos1
TargetNet	NOS1
TargetNet	PRSS1
TargetNet	Drd2
TargetNet	HTR1D
TargetNet	HTR1B
TargetNet	HTR2A
TargetNet	HCK
TargetNet	MAPK11
TargetNet	TYK2
TargetNet	SLC9A1
TargetNet	PTGER4
TargetNet	Mc5r
TargetNet	ITK
TargetNet	RXRA
TargetNet	CSF1R
TargetNet	CTSD

TargetNet	DRD2
TargetNet	F7
TargetNet	PLA2G2A
TargetNet	Adora3
TargetNet	P2RY1
TargetNet	KDR
TargetNet	EDNRB
TargetNet	GCK
TargetNet	CYP3A4
TargetNet	NOS2
TargetNet	KIF11
TargetNet	JAK2
TargetNet	OXTR
TargetNet	BDKRB2
TargetNet	S1PR3
TargetNet	Cckbr
TargetNet	Cckar
TargetNet	Ptger3
TargetNet	AGTR1
TargetNet	Oxtr
TargetNet	ECE1
TargetNet	XIAP
TargetNet	Mc4r
TargetNet	Prkca
TargetNet	ITGB1
TargetNet	PYGL
TargetNet	PLK1
TargetNet	MAPK9
TargetNet	PGGT1B
TargetNet	MAPK8
TargetNet	FOLH1
TargetNet	NR3C2
TargetNet	RPS6KB1
TargetNet	PLG
TargetNet	F10
TargetNet	Htr1b
TargetNet	MAP3K8
TargetNet	ITGAL
TargetNet	AVPR1B
TargetNet	Prcp
TargetNet	KCNA3
TargetNet	CXCR3
TargetNet	Faah
TargetNet	Hrh3
TargetNet	PLD2
TargetNet	MTNR1A
TargetNet	GPR119
TargetNet	Slc6a1
TargetNet	LIPE
TargetNet	FLT4
TargetNet	PIM3
TargetNet	CALCRL
TargetNet	CHRM2
TargetNet	MC4R
TargetNet	MAPK10
TargetNet	BRS3
TargetNet	CCR1
TargetNet	PNP

TargetNet	PTGER3
TargetNet	Ace
TargetNet	SSTR5
TargetNet	REN
TargetNet	DPP4
TargetNet	SLC5A1
TargetNet	ADRA1A
TargetNet	ADAM17
TargetNet	PDPK1
TargetNet	Scd1
TargetNet	SSTR3
TargetNet	Agtr2
TargetNet	Hsd11b1
TargetNet	MMP3
TargetNet	P2rx7
TargetNet	DRD3
TargetNet	EDNRB
TargetNet	NR2E3
TargetNet	NR3C1
TargetNet	HTR7
TargetNet	ALOX5AP
TargetNet	Chrm1
TargetNet	Chrm3
TargetNet	Avpr1a
TargetNet	GCGR
TargetNet	CCNE2
TargetNet	SLC6A2
TargetNet	CHEK2
TargetNet	P2RY2
TargetNet	PAK4
TargetNet	ATP4A
TargetNet	SRD5A1
TargetNet	DPP8
TargetNet	CASP7
TargetNet	CASP6
TargetNet	ADAM17
TargetNet	CCR5
TargetNet	HSD11B1
TargetNet	CCR8
TargetNet	Slc6a1
TargetNet	HTR1A
TargetNet	PDE4B
TargetNet	SLC6A4
TargetNet	Grm5
TargetNet	HRH1
TargetNet	Cckbr
TargetNet	SIGMAR1
TargetNet	LYN
TargetNet	SELE
TargetNet	KISS1R
TargetNet	TOP1
TargetNet	CASP1
TargetNet	CXCR4
TargetNet	MAPK1
TargetNet	PDE3A
TargetNet	HSP90AB1
TargetNet	DRD1
TargetNet	CLK4

TargetNet	ADRB3
TargetNet	MLYCD
TargetNet	Scd1
TargetNet	Soat1
TargetNet	SLC6A9
TargetNet	FAAH
TargetNet	Pde10a
TargetNet	TBXA2R
TargetNet	GPBAR1
TargetNet	ITGA2B
TargetNet	CYP2D6
TargetNet	NPY2R
TargetNet	AXL
TargetNet	BDKRB1
TargetNet	CCKAR
TargetNet	CCKBR
TargetNet	SSTR2
TargetNet	CYP11B1
TargetNet	SSTR1
TargetNet	FDPS
TargetNet	Tspo
TargetNet	PRCP
TargetNet	LCK
TargetNet	CDK9
TargetNet	AKT2
TargetNet	Htr7
TargetNet	Oprd1
TargetNet	Oprd1
TargetNet	TBXAS1
TargetNet	EGLN1
TargetNet	F3
TargetNet	DRD1
TargetNet	APH1B
TargetNet	CHRM3
TargetNet	56k.02
TargetNet	Tyms
TargetNet	Hsd11b2
TargetNet	OPRM1
TargetNet	Drd3
TargetNet	MMP1
TargetNet	BCL2
TargetNet	ADK
TargetNet	Mme
TargetNet	CDC7
TargetNet	Grm2
TargetNet	ITGA4
TargetNet	FFAR1
TargetNet	TPSAB1
TargetNet	Ache
TargetNet	IKBKB
TargetNet	Tbxas1
TargetNet	ROCK2
TargetNet	SCN9A
TargetNet	PSENEN
TargetNet	PNMT
TargetNet	Mapk14
TargetNet	DPP9
TargetNet	ELANE

TargetNet	HRH4
TargetNet	FLT1
TargetNet	MTTP
TargetNet	IRAK4
TargetNet	APH1A
TargetNet	NCSTN
TargetNet	PPARG
TargetNet	PIK3CB
TargetNet	PIK3CA
TargetNet	MMP13
TargetNet	MMP8
TargetNet	NTSR1
TargetNet	F2
TargetNet	F2
TargetNet	CMA1
TargetNet	AGTR1
TargetNet	PDE10A
TargetNet	HTR6
TargetNet	CTSK
TargetNet	SLC29A1
TargetNet	ADORA1
TargetNet	CDK7
TargetNet	DPP7
TargetNet	RXRG
TargetNet	PLA2G7
TargetNet	ITGAV
TargetNet	Slc6a4
TargetNet	TPSB2
TargetNet	AURKA
TargetNet	FKBP1A
TargetNet	FKBP1A
TargetNet	CCR2
TargetNet	P2RX7
TargetNet	UL80
TargetNet	PARP1
TargetNet	CAPN1
TargetNet	Htr2a
TargetNet	PIK3CD
TargetNet	PDE4A
TargetNet	MAPK14
TargetNet	PDE5A
TargetNet	TYRO3
TargetNet	HRH3
TargetNet	GNRHR
TargetNet	Gnrhr
TargetNet	CETP
TargetNet	BCL2L1
TargetNet	MMP7
TargetNet	PPARA
TargetNet	ABL1
TargetNet	TYMS
TargetNet	HRH3
TargetNet	DGAT1
TargetNet	EDNRA
TargetNet	SMO
TargetNet	PSEN1
TargetNet	FURIN
TargetNet	BRAF

TargetNet	UTS2R
TargetNet	CASP3
TargetNet	CNR1
