# Supplementary material for: Network Pharmacology Reveals That Resveratrol Can Alleviate COVID-19-Related Hyperinflammation
Source: Dis Markers. 2021 Sep 22;2021:4129993. doi: 10.1155/2021/4129993 (PMC8463930; doi:10.1155/2021/4129993)
Supplement: Supplementary 2 — Supplementary Table S2: SARS-CoV-2 DEGs. [file 4129993.f2.pdf]

SARS-CoV-2 DEGs

entrezID	Gene	logFC	t	P.Value	adj.P.Val	B
9580	CSF3	5.35878	6.51124	0.001065	0.11138	-0.16265
16941	CXCL5	3.79406	6.63459	0.000974	0.10812	-0.07115
1432	S100A7	3.65499	8.79362	0.000703	0.09825	0.39507
1418	SPRR2E	3.64936	16.4945	1.02E-05	0.01991	3.88821
13615	CCL20	3.17437	19.8807	3.87E-06	0.01262	4.46892
7436	PLA2G4E	3.07466	6.77971	0.005098	0.20608	-1.32957
17957	CSF2	3.02754	8.64976	0.000268	0.06303	1.2081
17087	DDIT4L	3.02443	7.77001	0.000455	0.08274	0.69581
19803	IL6	3.01195	8.67696	0.000264	0.06303	1.22293
1415	SPRR2D	2.9672	12.3332	4.51E-05	0.03337	2.79191
6953	IFI27	2.96693	8.98783	0.000222	0.05968	1.3885
19443	VNN3	2.87152	12.6512	3.96E-05	0.03232	2.89634
7503	HMGN2P4(	2.76209	4.99186	0.00645	0.22902	-1.80313
13014	IL36G	2.74713	20.1254	3.63E-06	0.01262	4.5034
9090	XAF1	2.74619	5.74572	0.001913	0.13573	-0.77119
17770	BHMT2	2.6845	12.6322	0.028952	0.34779	-2.82805
8579	CES1P1	2.65444	4.61811	0.008592	0.25187	-2.10981
3092	MIR936	2.61403	7.07112	0.001686	0.12957	-0.42646
14626	MX1	2.56067	8.58728	0.000278	0.06303	1.17381
443	IFI6	2.54648	4.84994	0.004117	0.18992	-1.58231
938	IFI44L	2.54412	4.58307	0.005273	0.20972	-1.8469
14354	SLCO4A1	2.5433	17.5711	0.000251	0.062	0.76653
18065	SLC4A9	2.53492	10.1214	0.006457	0.22902	-1.56491
7819	BCL2A1	2.51164	7.00557	0.000751	0.09825	0.19393
18665	HIST1H2AI	2.49766	3.28354	0.041357	0.38301	-3.44578
10348	L3MBTL4	2.4743	4.0693	0.008733	0.25267	-2.38818
751	PDZK1IP1	2.45498	9.39842	0.000178	0.05381	1.59645
11576	CEACAM7	2.44408	2.59518	0.04639	0.39779	-4.17224
3612	SAA2	2.43196	31.7637	3.37E-07	0.00551	5.48808
1430	S100A7A	2.4234	5.33356	0.010533	0.26981	-2.00846
14625	MX2	2.41422	4.92378	0.00385	0.18107	-1.51103
5634	SYCP3	2.3822	4.9238	0.006788	0.2331	-1.85743
21083	HTRA4	2.34225	16.8499	0.002083	0.14102	-1.11389
5251	KRT78	2.31326	4.65553	0.004926	0.20401	-1.77396
16942	CXCL3	2.30899	5.91717	0.001669	0.12957	-0.62854
16935	IL8	2.29924	20.8568	3.02E-06	0.01262	4.60149
14215	MMP9	2.26505	8.24438	0.00034	0.07097	0.98008
6798	ADAM20	2.22081	8.86727	0.00864	0.25187	-1.73815
3613	SAA1	2.20601	11.0132	8.01E-05	0.03608	2.3105
8899	IL17C	2.19126	3.71299	0.018547	0.31681	-2.94445
3611	SAA4	2.18721	4.01394	0.01418	0.29627	-2.65178
19444	VNN2	2.18428	3.06168	0.026371	0.34437	-3.57392
3334	SNORA52	2.12634	4.15352	0.008019	0.24676	-2.29652
1426	PGLYRP4	2.07863	25.8828	9.81E-07	0.00801	5.11622
10862	TNFSF14	2.07553	8.85711	0.000239	0.06181	1.31976
5562	LUM	2.05063	9.64943	0.040369	0.38082	-2.93621
10799	FSD1	2.05063	9.64943	0.040369	0.38082	-2.93621
16425	C3orf65	2.05063	9.64943	0.040369	0.38082	-2.93621
986	GBP5	2.04639	4.04879	0.008918	0.25371	-2.41068
11743	MIR320E	2.03953	9.59721	0.04064	0.38113	-2.9389
10923	ADAMTS1(	2.03559	3.47616	0.03555	0.36766	-3.28039
3607	MRGPRX3	2.03166	7.87998	0.000424	0.07961	0.76346
15157	NFAM1	2.01275	3.97479	0.014673	0.3011	-2.68898
13868	PCNA-AS1	1.98577	3.14057	0.046445	0.39783	-3.57313
22208	RNF183	1.96896	3.95419	0.00983	0.26209	-2.51535
19442	VNN1	1.95711	7.9684	0.000402	0.07783	0.81708

21657	RLN1	1.94249	5.47152	0.002395	0.14819	-1.00744
875	PDE4B	1.92141	3.33604	0.019213	0.31967	-3.23528
18790	TNF	1.91253	8.42399	0.000306	0.06565	1.08273
10215	C1QTNF1	1.90039	11.6074	6.14E-05	0.03386	2.53727
9323	SLC47A1	1.89308	13.8175	0.00324	0.16471	-1.25223
17121	RPL34-AS1	1.89308	13.8175	0.00324	0.16471	-1.25223
1108	SYPL2	1.89308	8.90811	0.044543	0.39147	-2.97801
2621	GLUD1P7	1.89308	8.90811	0.044543	0.39147	-2.97801
18607	HIST1H1T	1.89308	8.90811	0.044543	0.39147	-2.97801
8098	SYNGR3	1.8629	4.31938	0.006799	0.2331	-2.11937
14177	PI3	1.85823	6.84321	0.00084	0.10209	0.07975
18951	ETV7	1.8572	3.26158	0.020913	0.33203	-3.32609
9598	KRT24	1.85414	8.46199	0.000299	0.06507	1.10412
1429	S100A8	1.85057	11.8571	5.51E-05	0.03337	2.62754
22552	GRIN1	1.85025	6.66976	0.002123	0.142	-0.65451
13198	LY75	1.84986	2.59916	0.046161	0.3975	-4.16704
10969	ICAM1	1.84524	9.83086	0.000142	0.05033	1.80312
18830	CFB	1.84363	12.3442	4.49E-05	0.03337	2.79558
3741	C11orf94	1.84196	4.04697	0.013779	0.29315	-2.62061
14223	MKRN7P	1.82876	2.80712	0.035749	0.36766	-3.89736
18112	PCDHB11	1.82134	5.24518	0.005359	0.20979	-1.60681
19942	INHBA	1.81783	14.593	1.91E-05	0.02594	3.45323
16656	CPZ	1.7998	8.21846	0.010206	0.26813	-1.849
18617	HIST1H4E	1.7902	3.26267	0.020887	0.33203	-3.32476
16936	CXCL6	1.78204	5.19745	0.003023	0.1615	-1.25399
14959	SEC14L4	1.77906	4.72668	0.00461	0.19884	-1.70314
4449	BIRC3	1.72034	11.3099	7.00E-05	0.03386	2.42593
21841	CCIN	1.71908	3.25924	0.042171	0.38558	-3.46715
22774	XK	1.71737	3.14338	0.023968	0.34271	-3.47194
5746	OAS1	1.70105	11.0382	7.92E-05	0.03608	2.32042
18791	LTB	1.69426	10.4869	0.000103	0.03803	2.09445
23147	DRP2	1.68876	7.21997	0.001552	0.12727	-0.34579
2833	BEND3P3	1.67775	4.23051	0.020665	0.33034	-2.69773
1416	SPRR2A	1.67647	9.49321	0.000169	0.05381	1.6428
14695	ITGB2	1.67297	2.75475	0.038103	0.37458	-3.96487
14413	LINC0017(	1.66579	3.87407	0.010686	0.26998	-2.60515
579	ZC3H12A	1.64774	10.6952	9.29E-05	0.03608	2.18178
1144	CHI3L2	1.64017	4.56074	0.016665	0.30865	-2.52803
3304	IFITM1	1.63795	9.04345	0.000215	0.05968	1.41737
20132	NCF1	1.61766	3.64963	0.013574	0.29288	-2.86233
20078	GTF2IRD1F	1.60842	7.75905	0.001164	0.11657	-0.07003
15324	IRAK2	1.60827	13.137	3.27E-05	0.03194	3.04819
3320	IRF7	1.59423	6.23591	0.001306	0.11938	-0.37326
11087	MIR27A	1.5866	4.17517	0.043198	0.38771	-3.14785
14840	ZNF280A	1.5866	4.17517	0.043198	0.38771	-3.14785
15664	SLC38A3	1.5866	4.17517	0.043198	0.38771	-3.14785
9974	LOC64563	1.58493	3.10451	0.025079	0.34437	-3.52035
16897	SULT1B1	1.57431	3.57253	0.021121	0.33332	-3.08639
19984	SNORA9	1.57425	2.95197	0.038931	0.37765	-3.75428
19478	TNFAIP3	1.57167	13.3721	2.98E-05	0.03194	3.11848
1669	HSPA7	1.56368	3.11862	0.02467	0.34437	-3.50275
19620	CAHM	1.5538	3.83567	0.016598	0.30865	-2.82327
1808	ASTN1	1.55347	2.64998	0.043341	0.38771	-4.10075
18524	MAK	1.54977	6.44487	0.005953	0.2216	-1.45997
10207	DNAH17	1.53986	3.14115	0.046423	0.39783	-3.57261
1233	HIST2H2B/	1.53897	2.67229	0.042163	0.38558	-4.07173
17221	MGARP	1.53784	3.26138	0.020917	0.33203	-3.32633
9236	MGC12916	1.53268	6.91809	0.000797	0.10073	0.13276

3280	.OC61920	1.52905	4.2527	0.007262	0.23571	-2.19005
5156	RND1	1.52776	7.95411	0.000405	0.07783	0.80846
9919	C17orf67	1.52705	3.15509	0.023644	0.34271	-3.45739
7122	TNFAIP2	1.5269	8.93105	0.000229	0.06027	1.3588
5569	PLEKHG7	1.52402	3.73332	0.012404	0.28394	-2.76547
6527	MDP1	1.52289	18.6779	3.10E-05	0.03194	2.55796
17751	F2RL2	1.51457	14.0989	0.000508	0.08727	0.21022
4189	CARNS1	1.51457	11.0548	0.005314	0.20979	-1.46318
5297	.OC40004	1.51457	11.0548	0.005314	0.20979	-1.46318
2719	DNAJC12	1.51299	3.80443	0.01707	0.31081	-2.85388
13687	UGT1A1	1.51041	3.51404	0.022316	0.34001	-3.14651
22350	.OC38979	1.50441	3.94203	0.015101	0.30253	-2.7203
5236	KRT6B	1.49815	12.869	3.63E-05	0.03232	2.96555
19594	SOD2	1.479	10.7511	9.05E-05	0.03608	2.20479
17960	SLC22A4	1.46899	2.93979	0.030469	0.35413	-3.72778
7248	NORD116-	1.46609	5.74899	0.022112	0.33976	-2.47494
12671	.OC64483	1.46609	5.74899	0.022112	0.33976	-2.47494
10863	C3	1.46424	11.978	5.23E-05	0.03337	2.67022
21406	TMEM74	1.45528	4.0159	0.023931	0.34271	-2.85338
21094	C8orf4	1.44287	3.89394	0.010466	0.26962	-2.58278
11091	NANOS3	1.43733	3.76029	0.012052	0.28015	-2.7345
6964	SERPINA3	1.43618	6.78393	0.000876	0.10209	0.03736
16781	RHOH	1.43596	3.03858	0.035612	0.36766	-3.65716
22465	RNU6ATA	1.42413	2.70246	0.040625	0.38113	-4.03255
8700	PARD6A	1.41727	4.10497	0.013108	0.28675	-2.5663
19936	.OC64699	1.41416	3.49874	0.016013	0.30597	-3.03983
3995	SNORA57	1.40541	2.9815	0.028992	0.34779	-3.67491
21105	PLAT	1.38861	11.2926	7.06E-05	0.03386	2.41932
16944	CXCL2	1.38498	6.56058	0.001027	0.11053	-0.12584
3961	RPLP0P2	1.38033	3.78885	0.011691	0.27656	-2.70183
17426	TRIML2	1.37796	6.17618	0.001366	0.12019	-0.42014
16938	CXCL1	1.37763	13.0905	3.33E-05	0.03194	3.03405
10590	SERPINB4	1.37487	3.46306	0.01666	0.30865	-3.08234
19923	GPR141	1.37063	5.24467	0.005361	0.20979	-1.6072
14167	KCNK15	1.36743	3.55797	0.021411	0.33499	-3.1013
11187	BST2	1.35932	5.37057	0.002607	0.15449	-1.09701
21911	CBWD3	1.35361	8.62441	0.002416	0.14819	-0.63847
17551	TTC23L	1.35361	5.85634	0.021256	0.33415	-2.43872
7407	C15orf62	1.35027	8.82756	0.000243	0.06186	1.30404
4414	HEPHL1	1.33741	11.9328	5.33E-05	0.03337	2.65434
6995	HHIPL1	1.33373	3.42303	0.017421	0.31081	-3.13027
16360	CNMB2-IT	1.33329	5.32355	0.026037	0.34437	-2.62919
18186	SPINK6	1.32911	7.17384	0.004284	0.19312	-1.17884
22155	TAL2	1.32911	7.17384	0.004284	0.19312	-1.17884
8557	NOD2	1.32701	2.91081	0.031544	0.35525	-3.76463
15708	.INC0069	1.32236	2.81727	0.035311	0.36687	-3.88431
7909	RHCG	1.31899	10.7105	9.22E-05	0.03608	2.18806
14947	LIF	1.28596	12.7367	3.83E-05	0.03232	2.92374
4226	MYEOV	1.28551	7.30361	0.000614	0.09199	0.39641
5422	CYP27B1	1.28414	5.65892	0.002052	0.1407	-0.84488
5747	OAS3	1.28112	8.06905	0.000378	0.0752	0.87728
16759	FLJ13197	1.28011	9.34344	0.007702	0.24213	-1.66665
9303	CCDC144	1.28011	11.9163	0.000869	0.10209	-0.05701
18064	HBEGF	1.27899	11.9491	5.30E-05	0.03337	2.66007
19541	C1006527	1.27752	8.20871	0.000347	0.07097	0.95937
19061	C6orf223	1.27579	3.43061	0.017274	0.31081	-3.12117
16310	SPTSSB	1.27361	11.6343	0.000223	0.05968	1.34389
5235	KRT75	1.25747	5.02515	0.003517	0.1713	-1.41452

20324	SPDYE3	1.25601	8.53236	0.009401	0.25871	-1.79329
965	COL24A1	1.25087	2.67568	0.041987	0.38461	-4.06731
5748	OAS2	1.24603	9.01475	0.000219	0.05968	1.4025
19965	AEBP1	1.24407	5.66125	0.002048	0.1407	-0.84288
9810	ITGB3	1.24233	7.01867	0.000744	0.09825	0.20302
8691	TPPP3	1.23638	12.4673	0.000753	0.09825	0.20904
17133	RRH	1.23638	8.46586	0.009564	0.26011	-1.80475
21748	IFNK	1.23638	8.46586	0.009564	0.26011	-1.80475
6520	IRF9	1.22725	17.486	7.51E-06	0.01991	4.08067
15471	SLC22A14	1.22404	2.8744	0.032955	0.35978	-3.81109
1428	S100A12	1.22345	5.25297	0.002882	0.15996	-1.20317
7500	C15orf48	1.21887	15.918	1.22E-05	0.01991	3.76615
18764	MUC21	1.21514	6.67238	0.000948	0.10668	-0.04346
2722	MYPN	1.21422	3.73409	0.012394	0.28394	-2.76459
8161	IL32	1.20763	9.59918	0.00016	0.05327	1.69391
18228	TNIP1	1.19814	16.1682	1.13E-05	0.01991	3.8201
14370	HAR1B	1.19265	8.70506	0.008997	0.25371	-1.76432
16084	GATA2	1.19265	8.70506	0.008997	0.25371	-1.76432
3046	C1002895	1.19265	8.70506	0.008997	0.25371	-1.76432
21022	NUGGC	1.19265	8.70506	0.008997	0.25371	-1.76432
23264	SLC6A14	1.19015	11.8523	5.52E-05	0.03337	2.62581
4275	P2RY6	1.18984	5.30586	0.002754	0.15778	-1.15519
15263	C1001446	1.18926	3.90291	0.025914	0.34437	-3.01398
23033	IL2RG	1.18544	4.84028	0.007231	0.23571	-1.925
17288	TLR2	1.18196	4.0332	0.009061	0.25393	-2.42783
9683	STAT5A	1.18103	4.66397	0.004887	0.20292	-1.76552
8631	TEPP	1.18001	3.02376	0.027576	0.34456	-3.62158
3461	TRIM34	1.178	2.7232	0.049553	0.40479	-4.01649
16820	CWH43	1.16469	3.23607	0.021533	0.33593	-3.35739
8137	PRSS27	1.15966	3.51753	0.015684	0.3044	-3.01752
3201	LOC39981	1.15694	3.08778	0.025575	0.34437	-3.54124
15066	MAFF	1.15202	10.7912	8.88E-05	0.03608	2.22121
20941	GALNAC	1.14061	4.27843	0.007079	0.23571	-2.16269
22833	C1001339	1.12432	2.9758	0.029189	0.34779	-3.68212
20887	FAM167A	1.12388	5.86322	0.001742	0.12957	-0.67301
7492	DUOXA2	1.12228	4.35681	0.006555	0.22952	-2.08002
6104	EPSTI1	1.11908	4.26525	0.007172	0.23571	-2.17669
15097	SNORD43	1.11184	5.03603	0.003483	0.17049	-1.40426
7182	LOC64621	1.10785	4.13094	0.008204	0.24836	-2.32097
18535	EDN1	1.09935	7.66003	0.000487	0.08551	0.62705
7788	LOC64575	1.09882	4.38738	0.039015	0.37765	-3.03906
2546	MAP3K8	1.09582	13.5458	2.79E-05	0.03194	3.16915
18873	PSMB9	1.0936	2.97148	0.02934	0.34779	-3.68758
4461	MMP13	1.09308	4.1421	0.008112	0.24695	-2.30888
11798	PLA2G4C	1.09109	4.7271	0.004608	0.19884	-1.70272
21129	EFCAB1	1.09002	3.10947	0.024934	0.34437	-3.51415
2019	SLC26A9	1.08939	4.5594	0.009002	0.25371	-2.15991
16857	ARL9	1.08893	2.64089	0.043832	0.38922	-4.1126
18920	PACSIN1	1.08345	5.89135	0.003442	0.1692	-1.1449
9311	SLC5A10	1.07964	2.79099	0.036456	0.36981	-3.91812
10205	SOCS3	1.07648	7.20645	0.000655	0.09462	0.33139
5370	IL23A	1.06066	3.4291	0.017303	0.31081	-3.12298
20431	NRCAM	1.05937	5.63683	0.002089	0.14102	-0.86379
13011	IL1A	1.0488	6.35509	0.001194	0.11813	-0.281
14388	HELZ2	1.04458	3.31605	0.019654	0.32199	-3.25957
7601	C2CD4A	1.0412	3.53665	0.015357	0.30253	-2.99489
15256	TYMP	1.03291	4.34518	0.00663	0.22968	-2.09222
1427	S100A9	1.03014	11.3052	7.02E-05	0.03386	2.42412

15092	PDGFB	1.0248	8.49578	0.000293	0.06466	1.12303
18715	ZFP57	1.02128	3.82762	0.011221	0.27445	-2.6577
19175	LINC00472	1.02034	4.41911	0.006169	0.22622	-2.01501
7455	TGM5	1.01903	5.87172	0.00173	0.12957	-0.66598
3269	ADAM8	1.01675	5.55158	0.002241	0.14404	-0.9374
13012	IL1B	1.01325	10.796	8.86E-05	0.03608	2.22315
7240	NORD116-	1.01136	4.03816	0.046241	0.39755	-3.22164
17679	ADAMTS6	1.0079	2.62825	0.044523	0.39147	-4.12906
11730	IGFL1	1.00538	3.1136	0.024815	0.34437	-3.50901
9718	IFI35	1.00407	4.99449	0.003614	0.17373	-1.44355
17388	LOC38924	-1.00113	-6.18393	0.002854	0.15996	-0.95266
5726	MYL2	-1.00321	-5.68142	0.022677	0.34077	-2.49828
12970	CCDC138	-1.00433	-4.07394	0.008692	0.25207	-2.3831
9652	KRT15	-1.00436	-7.06134	0.000723	0.09825	0.23251
20658	FAM131B	-1.00478	-2.54372	0.049466	0.40479	-4.23959
19459	ALDH8A1	-1.00611	-2.74944	0.038351	0.3766	-3.97172
10699	EFNA2	-1.00757	-7.42013	0.001392	0.12019	-0.24047
5204	METTL7A	-1.00808	-6.90908	0.000802	0.10073	0.12641
11141	CYP4F3	-1.00897	-3.90055	0.010394	0.26908	-2.57535
4438	CNTN5	-1.02623	-5.70263	0.008624	0.25187	-1.83133
21135	ST18	-1.02623	-5.70263	0.008624	0.25187	-1.83133
15354	FBLN2	-1.02771	-2.55712	0.048645	0.40294	-4.22203
1212	VTCN1	-1.02839	-5.55818	0.002229	0.14404	-0.93167
16114	FAM86HP	-1.02902	-2.90363	0.031817	0.35525	-3.77379
7962	LOC91948	-1.02945	-3.15039	0.0318	0.35525	-3.5336
7506	SEMA6D	-1.03256	-5.0339	0.00349	0.17049	-1.40627
16730	PPARGC1A	-1.03601	-10.7351	9.11E-05	0.03608	2.1982
9551	LINC00672	-1.04151	-3.76795	0.011954	0.27827	-2.72572
23236	RGAG1	-1.04166	-2.81466	0.035423	0.36703	-3.88767
14292	BMP7	-1.04387	-3.64286	0.013674	0.29288	-2.87021
2826	ZMIZ1-AS1	-1.0448	-5.62062	0.002117	0.142	-0.87772
21136	FAM150A	-1.04977	-8.37445	0.002649	0.15475	-0.71623
11468	CATSPER(	-1.06101	-3.47817	0.016382	0.30865	-3.0643
21736	ELAVL2	-1.06595	-3.3326	0.019288	0.32019	-3.23946
14311	X16-NPEF	-1.06675	-2.6758	0.041981	0.38461	-4.06716
11663	IGSF23	-1.06816	-4.90025	0.013525	0.29288	-2.25863
597	POU3F1	-1.06864	-4.14059	0.012714	0.28579	-2.53322
8324	CRYM-AS1	-1.07864	-3.32237	0.019513	0.32066	-3.25189
18316	LOC25735	-1.08199	-3.91533	0.04923	0.40479	-3.29029
20738	ATG9B	-1.08201	-7.00425	0.000751	0.09825	0.19302
22171	PALM2	-1.09001	-3.0646	0.026281	0.34437	-3.57027
2640	ZNF488	-1.09522	-9.02529	0.000217	0.05968	1.40797
615	HPCAL4	-1.0959	-2.672	0.042178	0.38558	-4.0721
12459	CCDC121	-1.10466	-3.03245	0.027295	0.34437	-3.61064
13056	GLI2	-1.10598	-2.71938	0.03979	0.37893	-4.01062
16726	OC1005059	-1.11064	-3.11536	0.024764	0.34437	-3.50681
13095	OC1002164	-1.11441	-4.03265	0.046369	0.39779	-3.22467
4617	CCDC153	-1.11823	-2.73179	0.039189	0.37765	-3.99455
2714	MJD1C-AS	-1.12595	-4.55545	0.005413	0.21081	-1.87492
2695	FAM13C	-1.13385	-3.77722	0.011837	0.27794	-2.71511
11962	IGLON5	-1.1396	-3.98265	0.009545	0.26011	-2.48371
18626	HIST1H3G	-1.14475	-5.35786	0.010391	0.26908	-2.02423
10039	RGS9	-1.14609	-7.78566	0.011486	0.27625	-1.93293
8835	PKD1L2	-1.15057	-3.57109	0.014787	0.30218	-2.95426
8955	DBIL5P	-1.16092	-3.34335	0.019055	0.31916	-3.2264
3190	BTBD16	-1.16596	-3.33874	0.03958	0.37823	-3.48907
2256	NID1	-1.16997	-5.27567	0.002826	0.15925	-1.18253
11628	PINLYP	-1.17264	-3.78515	0.011737	0.27718	-2.70605

7863	SH3GL3	-1.17351	-2.73655	0.038961	0.37765	-3.98839
1794	SNORD74	-1.18326	-4.62762	0.005056	0.20576	-1.80195
17191	CETN4P	-1.19616	-4.51351	0.036795	0.36981	-2.97742
20374	)C1002895	-1.19624	-3.85521	0.0109	0.27069	-2.62644
17114	DKK2	-1.19832	-3.17395	0.023133	0.3422	-3.43402
19182	C6orf147	-1.20182	-3.44296	0.023876	0.34271	-3.22037
4535	DRD2	-1.2078	-4.11861	0.022293	0.33997	-2.77793
18110	PCDHB9	-1.2078	-4.11861	0.022293	0.33997	-2.77793
23194	RAB9B	-1.20864	-8.66542	0.00238	0.14819	-0.70885
3981	ASRGL1	-1.23211	-6.80398	0.000864	0.10209	0.05174
14754	PRODH	-1.23238	-3.1004	0.0252	0.34437	-3.52548
10920	PRAM1	-1.23244	-3.8015	0.027872	0.34501	-3.09522
20912	.OC72973:	-1.23697	-7.33677	0.013072	0.28675	-2.02974
23259	LRCH2	-1.23697	-7.33677	0.013072	0.28675	-2.02974
581	MIR5581	-1.23697	-4.17054	0.021518	0.33593	-2.80792
17943	SLC27A6	-1.26331	-3.98222	0.009549	0.26011	-2.48418
9873	.OC28408(	-1.27387	-4.05452	0.008866	0.25371	-2.40438
6730	SYT16	-1.28085	-4.84906	0.00412	0.18992	-1.58316
2046	CR1	-1.28951	-9.41204	0.007579	0.24008	-1.65697
15721	SEMA3G	-1.30062	-3.20386	0.030146	0.35291	-3.47526
4504	POU2AF1	-1.30574	-8.6359	0.00027	0.06303	1.20052
2482	CACNB2	-1.30735	-7.75427	0.011588	0.27642	-1.93936
18067	HD1-EIF4E	-1.30735	-7.75427	0.011588	0.27642	-1.93936
106	)C1001336	-1.3235	-2.78139	0.046566	0.3981	-3.94905
18848	PT2-EGFL	-1.32861	-8.32174	0.002701	0.15631	-0.80476
16450	MASP1	-1.3295	-6.76708	0.005127	0.20608	-1.33461
14479	LINC0011:	-1.33093	-3.98769	0.009495	0.26011	-2.47812
8361	PRKCB	-1.33329	-5.32355	0.026037	0.34437	-2.62919
1911	C1orf53	-1.34091	-11.414	0.000997	0.10988	0.02895
10539	)C1002872	-1.35102	-3.00884	0.028067	0.34566	-3.64037
23316	GRIA3	-1.35361	-5.85634	0.021256	0.33415	-2.43872
22621	ARSE	-1.35596	-3.03822	0.035625	0.36766	-3.65755
11821	NTN5	-1.35792	-5.68285	0.003953	0.18537	-1.28813
8541	ABCC11	-1.35932	-3.07544	0.025948	0.34437	-3.55669
18209	CSF1R	-1.35957	-3.08382	0.025694	0.34437	-3.5462
12531	ARHGEF3:	-1.36614	-4.05068	0.008901	0.25371	-2.4086
2150	CNIH3	-1.37581	-3.95756	0.048172	0.40178	-3.26642
20735	ABP1	-1.37623	-3.62702	0.031698	0.35525	-3.15572
16040	MYLK	-1.37841	-5.87363	0.001728	0.12957	-0.6644
18008	CXCL14	-1.3796	-5.11062	0.003261	0.16501	-1.33434
22711	MAP7D2	-1.38358	-5.15346	0.003141	0.16325	-1.29456
13466	CPO	-1.3888	-5.24043	0.026918	0.34437	-2.66146
12964	SULT1C2	-1.38993	-4.05369	0.013699	0.29288	-2.61428
10072	MAP2K6	-1.39489	-3.7276	0.012481	0.28483	-2.77205
8023	MIR3176	-1.39606	-6.69869	0.00093	0.10543	-0.02427
22653	FRMPD4	-1.40875	-3.23857	0.029126	0.34779	-3.43765
18775	HCG27	-1.41195	-3.39066	0.025106	0.34437	-3.27528
20252	PDK4	-1.41498	-3.18551	0.022825	0.34077	-3.41972
3362	TNNI2	-1.41609	-3.53154	0.015443	0.30253	-3.00094
4470	CASP5	-1.41825	-5.06871	0.012247	0.28306	-2.19717
4827	ACRBP	-1.4255	-3.25009	0.02119	0.33376	-3.34017
13034	.OC44090(	-1.42899	-3.965	0.0248	0.34437	-2.96511
9117	SLC2A4	-1.43153	-5.80835	0.003636	0.17373	-1.20128
1280	FMO5	-1.43261	-4.99592	0.003609	0.17373	-1.44219
22703	PHKA2-AS	-1.44035	-3.8167	0.027567	0.34456	-3.08293
13667	C2orf82	-1.44064	-5.67065	0.002033	0.1407	-0.83486
5984	TPTE2P1	-1.44174	-9.14928	0.000203	0.05968	1.47168
9107	ASGR1	-1.44961	-3.00241	0.036956	0.36981	-3.69757

4963	.OH12CR2	-1.45308	-3.3829	0.018223	0.31367	-3.17856
16151	KY	-1.46331	-3.90845	0.015556	0.30392	-2.7526
22182	ZNF483	-1.48678	-3.11342	0.02482	0.34437	-3.50924
12591	STON1	-1.49519	-5.86887	0.001734	0.12957	-0.66833
15768	DNAH12	-1.5	-3.1684	0.045396	0.39529	-3.64392
8577	SLC6A2	-1.5133	-2.75959	0.037879	0.37416	-3.95861
3359	IFITM10	-1.52076	-6.04644	0.001509	0.12501	-0.52348
10458	ASXL3	-1.52372	-4.0176	0.009207	0.25595	-2.44503
13531	SLC11A1	-1.52394	-3.81316	0.011394	0.27594	-2.67413
3166	NANOS1	-1.52444	-4.30548	0.006893	0.23374	-2.13405
9369	FOXN1	-1.52514	-3.4452	0.016994	0.31081	-3.10369
9222	DNAH9	-1.54297	-3.70493	0.012788	0.28579	-2.7982
21417	.INC00536	-1.54977	-4.12459	0.022202	0.33981	-2.84245
20959	LGI3	-1.55409	-4.0435	0.008966	0.25371	-2.41649
23418	MCF2	-1.56082	-3.03372	0.027254	0.34437	-3.60904
18886	MIR219-1	-1.56464	-6.73748	0.005197	0.20791	-1.32829
11275	ZNF826P	-1.5655	-4.28969	0.040867	0.38216	-3.08833
18787	ATP6V1G2	-1.56686	-3.41004	0.017676	0.31081	-3.14586
6632	KLHDC1	-1.56768	-3.42626	0.017358	0.31081	-3.12638
13750	SNED1	-1.58335	-3.09382	0.033666	0.36001	-3.59585
16329	LRRC34	-1.60099	-4.32329	0.019429	0.32059	-2.63278
2916	)C1001889	-1.60194	-3.10632	0.033244	0.35988	-3.58206
3111	RBM20	-1.60325	-4.98285	0.003652	0.17373	-1.45461
17074	ADH6	-1.60581	-3.75187	0.028898	0.34779	-3.13563
12139	NLRP7	-1.60635	-5.47766	0.009728	0.26173	-1.93057
13361	ANKAR	-1.61342	-4.94639	0.003773	0.17794	-1.48937
22645	)C1002888	-1.61441	-4.42371	0.038357	0.3766	-3.02108
8776	TAT	-1.62966	-3.41698	0.024478	0.34437	-3.2476
13267	DLX2	-1.63806	-3.44385	0.023856	0.34271	-3.21945
2821	DLG5-AS1	-1.64497	-2.58457	0.047007	0.39931	-4.1861
23230	KCNE1L	-1.64957	-2.6754	0.042002	0.38461	-4.06768
17291	DCHS2	-1.65143	-8.40567	0.002618	0.15449	-0.78072
17086	.OC256880	-1.65871	-6.1867	0.018898	0.31791	-2.33348
11802	ZNF114	-1.67808	-2.84442	0.034169	0.36309	-3.84946
16056	ALG1L	-1.67961	-3.7475	0.017973	0.313	-2.91009
12395	PFN4	-1.68122	-3.43193	0.017248	0.31081	-3.11959
16508	.OC401109	-1.69921	-2.58504	0.04698	0.39931	-4.1855
721	RPS15AP1	-1.71526	-7.5878	0.001273	0.11938	-0.15494
5725	CCDC63	-1.73697	-8.17347	0.049511	0.40479	-3.02874
10434	AQP4	-1.73697	-8.17347	0.049511	0.40479	-3.02874
6035	FRY	-1.75115	-2.60974	0.045558	0.39583	-4.15322
2710	EGR2	-1.75279	-8.54413	0.00079	0.10068	0.28995
935	GIPC2	-1.76858	-2.66533	0.042527	0.38609	-4.08078
13822	SNORD57	-1.7826	-5.25999	0.026707	0.34437	-2.6538
15034	IL2RB	-1.78469	-2.9232	0.03108	0.35487	-3.74886
10861	CD70	-1.79361	-12.8593	0.000682	0.09675	0.07054
4807	ANO2	-1.84861	-2.95039	0.038994	0.37765	-3.75606
14441	SAMSN1	-1.85798	-8.74292	0.04558	0.39583	-2.98853
21086	ADAM32	-1.86283	-16.2908	1.08E-05	0.01991	3.84599
9313	GRAP	-1.91446	-9.7445	0.001645	0.12957	-0.44687
8769	)C1001308	-1.91825	-7.65919	0.011904	0.27821	-1.95914
22158	MIR548Q	-1.91825	-7.65919	0.011904	0.27821	-1.95914
16166	NME9	-1.97315	-5.37858	0.002589	0.15449	-1.08986
21274	SLC10A5	-1.97321	-11.9598	0.000859	0.10209	0.12612
4815	CD27	-1.98039	-4.84171	0.007223	0.23571	-1.92383
7613	)C1001308	-1.98906	-8.85784	0.002222	0.14404	-0.56903
4739	OPCML	-2	-9.41121	0.041631	0.38404	-2.94876
15633	CCDC36	-2.03953	-9.59721	0.04064	0.38113	-2.9389

19623	PDE10A	-2.05664	-14.7452	0.00044	0.08161	0.27268
1384	HRNR	-2.05817	-9.26203	0.007851	0.24404	-1.67834
22095	)C1004994	-2.05889	-9.68834	0.04017	0.38082	-2.93424
16962	ART3	-2.07641	-21.3634	1.76E-05	0.02594	2.80491
7775	C15orf27	-2.09519	-3.45108	0.023691	0.34271	-3.2119
19764	THSD7A	-2.19935	-2.87091	0.033094	0.35978	-3.81556
21309	LRRC69	-2.21905	-4.55021	0.009068	0.25393	-2.1678
13381	DNAH7	-2.23201	-2.81874	0.035248	0.36667	-3.88243
1844	ZNF648	-2.32193	-10.9261	0.034635	0.36557	-2.88052
5998	SNORD10:	-2.36257	-11.1173	0.033901	0.3611	-2.87357
4417	GPR83	-2.4021	-11.3033	0.033214	0.35988	-2.86711
5905	RIMBP2	-2.6845	-12.6322	0.028952	0.34779	-2.82805
16279	LEKR1	-2.71621	-12.7814	0.028534	0.34722	-2.82433
2489	NEBL-AS1	-2.80735	-26.1331	6.97E-05	0.03386	0.79884
9713	ES3L-AAF	-2.80735	-26.1331	6.97E-05	0.03386	0.79884
14895	)C1001285	-2.80735	-26.1331	6.97E-05	0.03386	0.79884
755	FOXE3	-2.80735	-20.4907	0.001346	0.11938	-1.01428
1773	TNFSF18	-2.80735	-20.4907	0.001346	0.11938	-1.01428
2305	.OC14913	-2.80735	-20.4907	0.001346	0.11938	-1.01428
3651	SLC5A12	-2.80735	-20.4907	0.001346	0.11938	-1.01428
4998	PTPRO	-2.80735	-20.4907	0.001346	0.11938	-1.01428
11294	ZNF98	-2.80735	-20.4907	0.001346	0.11938	-1.01428
13136	LCT	-2.80735	-20.4907	0.001346	0.11938	-1.01428
13672	SCARNA5	-2.80735	-20.4907	0.001346	0.11938	-1.01428
15959	CD200R1	-2.80735	-20.4907	0.001346	0.11938	-1.01428
23175	RAB40AL	-2.80735	-20.4907	0.001346	0.11938	-1.01428
183	DRAXIN	-2.80735	-13.2103	0.027393	0.34437	-2.81426
968	CLCA1	-2.80735	-13.2103	0.027393	0.34437	-2.81426
1075	AMY2A	-2.80735	-13.2103	0.027393	0.34437	-2.81426
1311	HIST2H2AI	-2.80735	-13.2103	0.027393	0.34437	-2.81426
3391	SNORA54	-2.80735	-13.2103	0.027393	0.34437	-2.81426
3737	FZp779M0	-2.80735	-13.2103	0.027393	0.34437	-2.81426
4108	MIR612	-2.80735	-13.2103	0.027393	0.34437	-2.81426
5005	SKP1P2	-2.80735	-13.2103	0.027393	0.34437	-2.81426
5271	AMHR2	-2.80735	-13.2103	0.027393	0.34437	-2.81426
6114	LINC0033(	-2.80735	-13.2103	0.027393	0.34437	-2.81426
6778	ACTN1-AS	-2.80735	-13.2103	0.027393	0.34437	-2.81426
6835	SYNDIG1L	-2.80735	-13.2103	0.027393	0.34437	-2.81426
7746	CPLX3	-2.80735	-13.2103	0.027393	0.34437	-2.81426
8337	VWA3A	-2.80735	-13.2103	0.027393	0.34437	-2.81426
8947	DOC2B	-2.80735	-13.2103	0.027393	0.34437	-2.81426
9365	VTN	-2.80735	-13.2103	0.027393	0.34437	-2.81426
9700	RAMP2-AS	-2.80735	-13.2103	0.027393	0.34437	-2.81426
10233	MIR4730	-2.80735	-13.2103	0.027393	0.34437	-2.81426
10455	WBP11P1	-2.80735	-13.2103	0.027393	0.34437	-2.81426
10486	.OC28426	-2.80735	-13.2103	0.027393	0.34437	-2.81426
10844	)C1001285	-2.80735	-13.2103	0.027393	0.34437	-2.81426
12161	TMEM190	-2.80735	-13.2103	0.027393	0.34437	-2.81426
13710	ESPNL	-2.80735	-13.2103	0.027393	0.34437	-2.81426
14355	)C1001278	-2.80735	-13.2103	0.027393	0.34437	-2.81426
15033	TMPRSS6	-2.80735	-13.2103	0.027393	0.34437	-2.81426
15663	GNAT1	-2.80735	-13.2103	0.027393	0.34437	-2.81426
16779	.OC34496	-2.80735	-13.2103	0.027393	0.34437	-2.81426
16881	SYT14L	-2.80735	-13.2103	0.027393	0.34437	-2.81426
16883	IPRSS11B	-2.80735	-13.2103	0.027393	0.34437	-2.81426
17973	SOWAHA	-2.80735	-13.2103	0.027393	0.34437	-2.81426
18344	C5orf47	-2.80735	-13.2103	0.027393	0.34437	-2.81426
19173	MIR30C2	-2.80735	-13.2103	0.027393	0.34437	-2.81426

19511	ZC2HC1B	-2.80735	-13.2103	0.027393	0.34437	-2.81426
20387	POLR2J2	-2.80735	-13.2103	0.027393	0.34437	-2.81426
20885	TDH	-2.80735	-13.2103	0.027393	0.34437	-2.81426
5525	PTPRQ	-2.81401	-7.82233	0.003276	0.16501	-0.95663
3317	MIR210	-2.82424	-15.9943	0.00234	0.14743	-1.14623
6863	ESRRB	-2.84539	-2.94947	0.039032	0.37765	-3.7571
20736	KCNH2	-2.92821	-13.4255	0.000594	0.09199	0.13788
4937	TAS2R10	-2.98864	-11.933	0.004487	0.1973	-1.38365
9567	PNMT	-2.98864	-11.933	0.004487	0.1973	-1.38365
19599	SNORA20	-2.98864	-11.933	0.004487	0.1973	-1.38365
22445	SNORD36	-2.98864	-11.933	0.004487	0.1973	-1.38365
8828	DYNLRB2	-3.16993	-14.9164	0.02357	0.34271	-2.78177
10493	SIGLEC15	-3.16993	-14.9164	0.02357	0.34271	-2.78177
18601	HIST1H2A	-3.16993	-14.9164	0.02357	0.34271	-2.78177
19103	CRISP3	-3.17387	-5.41999	0.01004	0.26597	-1.96144
11487	LOC64366	-3.2049	-5.0585	0.01232	0.28394	-2.16438
20910	FAM86B2	-3.33568	-3.98665	0.024426	0.34437	-2.8752
14701	SSR4P1	-3.35073	-3.19123	0.030528	0.35431	-3.489
10318	CLUL1	-3.35712	-5.16167	0.027793	0.34501	-2.69271
14393	TNFRSF6	-3.35712	-5.16167	0.027793	0.34501	-2.69271
7237	NORD116-	-3.40368	-4.84083	0.031804	0.35525	-2.82725
12605	C2orf73	-3.40368	-4.84083	0.031804	0.35525	-2.82725
14019	TSPY26P	-3.40368	-4.84083	0.031804	0.35525	-2.82725
16326	TERC	-3.40368	-4.84083	0.031804	0.35525	-2.82725
16471	UTS2D	-3.40368	-4.84083	0.031804	0.35525	-2.82725
21709	IFNB1	-3.40368	-4.84083	0.031804	0.35525	-2.82725
15557	CCR3	-3.5072	-3.1756	0.04513	0.39318	-3.54156
17030	HSD17B1	-3.57142	-5.84499	0.008007	0.24676	-1.74311
9028	ATP2A3	-3.58496	-7.18203	0.013692	0.29288	-2.06566
19818	STK31	-3.58496	-7.18203	0.013692	0.29288	-2.06566
16261	HGEF26-	-3.73322	-4.93682	0.013234	0.28831	-2.23655
20141	STAG3L1	-3.90689	-18.3843	0.018191	0.31367	-2.73989
21837	LOC15837	-3.90689	-18.3843	0.018191	0.31367	-2.73989
21929	OC1005072	-3.90689	-18.3843	0.018191	0.31367	-2.73989
17370	SPATA4	-3.95345	-26.8567	0.000735	0.09825	-0.9216
1114	MIR197	-4	-18.8224	0.017668	0.31081	-2.73609
10907	CCL25	-4	-18.8224	0.017668	0.31081	-2.73609
12660	LOC33980	-4	-18.8224	0.017668	0.31081	-2.73609
15165	ATP5L2	-4	-18.8224	0.017668	0.31081	-2.73609
20268	BHLHA15	-4	-18.8224	0.017668	0.31081	-2.73609
14313	MIR296	-4.16096	-18.0023	0.001797	0.12981	-1.07654
951	C1orf180	-4.27537	-9.55116	0.007338	0.23571	-1.63777
18041	SNORD63	-4.32193	-20.3373	0.01605	0.30627	-2.72472
10501	ST8SIA5	-4.35727	-3.51718	0.034447	0.365	-3.24607
1456	NUP210L	-4.45943	-20.9843	0.015439	0.30253	-2.72056
6436	RNASE6	-4.45943	-20.9843	0.015439	0.30253	-2.72056
13366	C2orf88	-4.49728	-5.70437	0.008616	0.25187	-1.81306
18210	PDGFRB	-5.52356	-25.9917	0.011838	0.27794	-2.69788
