# Supplementary material for: Network Pharmacology Reveals That Resveratrol Can Alleviate COVID-19-Related Hyperinflammation
Source: Dis Markers. 2021 Sep 22;2021:4129993. doi: 10.1155/2021/4129993 (PMC8463930; doi:10.1155/2021/4129993)
Supplement: Supplementary 3 — Supplementary Table S3: resveratrol-related targets GO analysis-enriched terms. [file 4129993.f3.pdf]

Resveratrol-related targets GO analysis enriched terms

Category	Term	Description	P	InTerm_InList
GO Molecular Functions	GO:0004672	protein kinase activity	9.196E-48	64/606
GO Molecular Functions	GO:0016773	phosphotransferase activity, alcohol group as acceptor	1.054E-45	66/711
GO Biological Processes	GO:0018105	peptidyl-serine phosphorylation	4.604E-44	48/303
GO Biological Processes	GO:0018209	peptidyl-serine modification	7.759E-44	49/326
GO Molecular Functions	GO:0016301	kinase activity	2.791E-43	66/776
GO Molecular Functions	GO:0004674	protein serine/threonine kinase activity	4.906E-29	42/446
GO Biological Processes	GO:0018107	peptidyl-threonine phosphorylation	9.105E-24	23/116
GO Biological Processes	GO:0018210	peptidyl-threonine modification	4.635E-23	23/124
GO Biological Processes	GO:1901699	cellular response to nitrogen compound	1.971E-45	64/660
GO Biological Processes	GO:0032870	cellular response to hormone stimulus	4.916E-40	61/712
GO Biological Processes	GO:0071417	cellular response to organonitrogen compound	6.679E-40	57/597
GO Biological Processes	GO:1901652	response to peptide	4.977E-29	45/534
GO Biological Processes	GO:1901653	cellular response to peptide	2.274E-24	36/397
GO Biological Processes	GO:0043434	response to peptide hormone	9.631E-23	36/443
GO Biological Processes	GO:0071375	cellular response to peptide hormone stimulus	1.187E-21	31/327
GO Biological Processes	GO:0032868	response to insulin	4.675E-17	25/279
GO Biological Processes	GO:0032869	cellular response to insulin stimulus	5.078E-16	22/223
GO Biological Processes	GO:0008286	insulin receptor signaling pathway	6.818E-14	17/145
GO Biological Processes	GO:0007169	transmembrane receptor protein tyrosine kinase signaling pathway	3.002E-40	62/737
GO Biological Processes	GO:0070848	response to growth factor	1.068E-19	41/736
GO Biological Processes	GO:0071363	cellular response to growth factor stimulus	1.241E-18	39/706
GO Biological Processes	GO:0071559	response to transforming growth factor beta	3.046E-08	15/253
GO Biological Processes	GO:0071560	cellular response to transforming growth factor beta stimulus	1E-06	13/247
GO Biological Processes	GO:0090287	regulation of cellular response to growth factor stimulus	7.3E-06	13/296
GO Biological Processes	GO:0007178	transmembrane receptor protein serine/threonine kinase signaling pathway	1.14E-05	14/356
GO Biological Processes	GO:0007179	transforming growth factor beta receptor signaling pathway	0.0001408	9/197
GO Biological Processes	GO:0071407	cellular response to organic cyclic compound	1.768E-37	53/544
GO Biological Processes	GO:0071396	cellular response to lipid	1.962E-29	48/616
GO Biological Processes	GO:0048545	response to steroid hormone	6.865E-26	37/386

GO Molecular Functions	GO:0098531	transcription factor activity, direct ligand regulated sequence-specific DNA binding	4.422E-21	16/47
GO Molecular Functions	GO:0004879	nuclear receptor activity	4.422E-21	16/47
GO Molecular Functions	GO:0003707	steroid hormone receptor activity	1.134E-19	16/56
GO Biological Processes	GO:0030522	intracellular receptor signaling pathway	4.391E-19	27/282
GO Biological Processes	GO:0071383	cellular response to steroid hormone stimulus	3.054E-18	25/249
GO Biological Processes	GO:0043401	steroid hormone mediated signaling pathway	9.762E-17	21/182
GO Biological Processes	GO:0009755	hormone-mediated signaling pathway	1.483E-14	21/233
GO Molecular Functions	GO:0031406	carboxylic acid binding	5.46E-14	20/220
GO Molecular Functions	GO:0043177	organic acid binding	1.495E-13	20/232
GO Molecular Functions	GO:0008134	transcription factor binding	2.42E-12	30/659
GO Biological Processes	GO:0006367	transcription initiation from RNA polymerase II promoter	6.335E-12	17/191
GO Molecular Functions	GO:0033293	monocarboxylic acid binding	1.141E-11	12/77
GO Biological Processes	GO:0006352	DNA-templated transcription, initiation	5.321E-10	17/253
GO Molecular Functions	GO:0008289	lipid binding	6.404E-09	27/755
GO Molecular Functions	GO:0030374	nuclear receptor transcription coactivator activity	2.004E-08	9/68
GO Molecular Functions	GO:0005496	steroid binding	3.806E-07	9/95
GO Cellular Components	GO:0005667	transcription factor complex	5.433E-07	16/360
GO Molecular Functions	GO:0003713	transcription coactivator activity	4.499E-06	14/328
GO Molecular Functions	GO:0000978	RNA polymerase II proximal promoter sequence-specific DNA binding	8.012E-06	19/604
GO Molecular Functions	GO:0000987	proximal promoter sequence-specific DNA binding	1.413E-05	19/629
GO Cellular Components	GO:0090575	RNA polymerase II transcription factor complex	3.116E-05	9/162
GO Molecular Functions	GO:0003712	transcription coregulator activity	7.216E-05	17/590
GO Cellular Components	GO:0044798	nuclear transcription factor complex	0.0001638	9/201
GO Biological Processes	GO:0009611	response to wounding	2.379E-35	56/691
GO Biological Processes	GO:0042060	wound healing	1.057E-29	47/576
GO Biological Processes	GO:0050878	regulation of body fluid levels	8.413E-27	42/507
GO Biological Processes	GO:0007596	blood coagulation	1.724E-23	33/336
GO Biological Processes	GO:0007599	hemostasis	2.76E-23	33/341
GO Biological Processes	GO:0050817	coagulation	3.03E-23	33/342
GO Biological Processes	GO:0030168	platelet activation	5.139E-17	20/154
GO Biological Processes	GO:0003013	circulatory system process	1.036E-33	50/554

GO Biological Processes	GO:0008015	blood circulation	5.495E-33	49/544
GO Biological Processes	GO:0044057	regulation of system process	2.018E-24	43/617
GO Biological Processes	GO:1903522	regulation of blood circulation	1.665E-20	29/301
GO Biological Processes	GO:0003015	heart process	1.616E-16	25/294
GO Biological Processes	GO:0060047	heart contraction	7.967E-15	23/284
GO Biological Processes	GO:0003012	muscle system process	6.914E-13	26/462
GO Biological Processes	GO:0090257	regulation of muscle system process	1.017E-12	20/257
GO Biological Processes	GO:0008016	regulation of heart contraction	6.796E-11	18/254
GO Biological Processes	GO:0006936	muscle contraction	4.231E-10	20/359
GO Biological Processes	GO:0060048	cardiac muscle contraction	1.122E-08	12/138
GO Biological Processes	GO:0006937	regulation of muscle contraction	1.303E-07	12/172
GO Biological Processes	GO:0006941	striated muscle contraction	1.675E-07	12/176
GO Biological Processes	GO:1903523	negative regulation of blood circulation	1.692E-06	6/38
GO Biological Processes	GO:0045822	negative regulation of heart contraction	8.196E-06	5/29
GO Biological Processes	GO:0055117	regulation of cardiac muscle contraction	1.543E-05	7/82
GO Biological Processes	GO:0006942	regulation of striated muscle contraction	4.608E-05	7/97
GO Biological Processes	GO:0045932	negative regulation of muscle contraction	0.0001488	4/28
GO Biological Processes	GO:0030335	positive regulation of cell migration	1.738E-33	50/560
GO Biological Processes	GO:0040017	positive regulation of locomotion	1.046E-32	51/612
GO Biological Processes	GO:2000147	positive regulation of cell motility	1.296E-32	50/584
GO Biological Processes	GO:0051272	positive regulation of cellular component movement	5.081E-32	50/601
GO Biological Processes	GO:0001568	blood vessel development	2.854E-22	45/777
GO Biological Processes	GO:0048514	blood vessel morphogenesis	7.689E-20	40/690
GO Biological Processes	GO:0001525	angiogenesis	2.012E-19	37/594
GO Biological Processes	GO:0050673	epithelial cell proliferation	6.847E-14	27/455
GO Biological Processes	GO:0010631	epithelial cell migration	1.402E-13	24/359
GO Biological Processes	GO:0001667	ameboidal-type cell migration	1.644E-13	27/472
GO Biological Processes	GO:0090132	epithelium migration	1.679E-13	24/362
GO Biological Processes	GO:0090130	tissue migration	2.399E-13	24/368
GO Biological Processes	GO:0050678	regulation of epithelial cell proliferation	1.161E-12	24/396
GO Biological Processes	GO:1901342	regulation of vasculature development	1.179E-12	25/434

GO Biological Processes	GO:0010634	positive regulation of epithelial cell migration	1.532E-12	17/175
GO Biological Processes	GO:0001936	regulation of endothelial cell proliferation	3.465E-12	17/184
GO Biological Processes	GO:0043542	endothelial cell migration	4.942E-12	20/280
GO Biological Processes	GO:0001935	endothelial cell proliferation	1.327E-11	17/200
GO Biological Processes	GO:0010632	regulation of epithelial cell migration	1.446E-11	20/297
GO Biological Processes	GO:0050679	positive regulation of epithelial cell proliferation	2.889E-11	17/210
GO Biological Processes	GO:0010595	positive regulation of endothelial cell migration	4.126E-11	14/131
GO Biological Processes	GO:0045765	regulation of angiogenesis	4.715E-11	22/392
GO Biological Processes	GO:0010594	regulation of endothelial cell migration	1.587E-10	17/234
GO Biological Processes	GO:0045766	positive regulation of angiogenesis	2.078E-10	16/206
GO Biological Processes	GO:0043536	positive regulation of blood vessel endothelial cell migration	3.395E-10	11/80
GO Biological Processes	GO:0001938	positive regulation of endothelial cell proliferation	1.017E-09	12/112
GO Biological Processes	GO:1904018	positive regulation of vasculature development	1.269E-09	16/233
GO Biological Processes	GO:0043534	blood vessel endothelial cell migration	3.124E-08	13/183
GO Biological Processes	GO:0043535	regulation of blood vessel endothelial cell migration	5.487E-08	12/159
GO Biological Processes	GO:0002040	sprouting angiogenesis	2.491E-06	11/188
GO Biological Processes	GO:1903672	positive regulation of sprouting angiogenesis	3.25E-06	7/65
GO Biological Processes	GO:0051347	positive regulation of transferase activity	3.255E-33	54/692
GO Biological Processes	GO:0033674	positive regulation of kinase activity	1.132E-32	51/613
GO Biological Processes	GO:0045860	positive regulation of protein kinase activity	8.951E-28	44/540
GO Biological Processes	GO:0071900	regulation of protein serine/threonine kinase activity	4.202E-26	42/528
GO Biological Processes	GO:0043410	positive regulation of MAPK cascade	2.406E-24	41/552
GO Biological Processes	GO:0043408	regulation of MAPK cascade	1.07E-23	46/754
GO Biological Processes	GO:0071902	positive regulation of protein serine/threonine kinase activity	4.904E-21	31/343
GO Biological Processes	GO:0043405	regulation of MAP kinase activity	6.683E-19	29/344
GO Biological Processes	GO:0032147	activation of protein kinase activity	3.495E-18	28/335
GO Biological Processes	GO:0043406	positive regulation of MAP kinase activity	1.141E-17	25/263
GO Biological Processes	GO:0070371	ERK1 and ERK2 cascade	2.09E-15	25/328
GO Biological Processes	GO:0070372	regulation of ERK1 and ERK2 cascade	3.456E-12	21/308
GO Biological Processes	GO:0070374	positive regulation of ERK1 and ERK2 cascade	5.613E-11	17/219
GO Biological Processes	GO:0000187	activation of MAPK activity	3.465E-07	11/154
GO Biological Processes	GO:0048511	rhythmic process	2.039E-32	39/299

GO Biological Processes	GO:0007623	circadian rhythm	6.348E-25	29/211
GO Biological Processes	GO:0042752	regulation of circadian rhythm	2.459E-14	16/115
GO Biological Processes	GO:0010035	response to inorganic substance	4.136E-30	47/564
GO Biological Processes	GO:0010038	response to metal ion	2.867E-17	28/363
GO Biological Processes	GO:0071241	cellular response to inorganic substance	5.613E-11	17/219
GO Biological Processes	GO:0046686	response to cadmium ion	5.997E-10	10/64
GO Biological Processes	GO:0071248	cellular response to metal ion	6.642E-10	15/191
GO Biological Processes	GO:0071276	cellular response to cadmium ion	2.708E-09	8/38
GO Biological Processes	GO:0002237	response to molecule of bacterial origin	2.221E-29	39/358
GO Biological Processes	GO:0032496	response to lipopolysaccharide	3.793E-29	38/338
GO Biological Processes	GO:0009617	response to bacterium	2.172E-23	45/729
GO Biological Processes	GO:1903530	regulation of secretion by cell	7.522E-19	41/777
GO Biological Processes	GO:0071216	cellular response to biotic stimulus	3.054E-18	25/249
GO Biological Processes	GO:0071219	cellular response to molecule of bacterial origin	6.135E-16	22/225
GO Biological Processes	GO:0071222	cellular response to lipopolysaccharide	2.219E-15	21/212
GO Biological Processes	GO:0001817	regulation of cytokine production	5.334E-13	33/755
GO Biological Processes	GO:0002790	peptide secretion	9.086E-12	29/649
GO Biological Processes	GO:0002791	regulation of peptide secretion	9.927E-12	26/520
GO Biological Processes	GO:0009306	protein secretion	1.326E-11	28/614
GO Biological Processes	GO:0050708	regulation of protein secretion	1.804E-11	25/492
GO Biological Processes	GO:0051047	positive regulation of secretion	5.778E-11	23/436
GO Biological Processes	GO:1903532	positive regulation of secretion by cell	9.246E-11	22/406
GO Biological Processes	GO:0002793	positive regulation of peptide secretion	1.023E-10	19/295
GO Biological Processes	GO:0001819	positive regulation of cytokine production	2.24E-10	23/467
GO Biological Processes	GO:0050714	positive regulation of protein secretion	2.345E-10	18/274
GO Biological Processes	GO:0050663	cytokine secretion	2.282E-07	14/255
GO Biological Processes	GO:0050715	positive regulation of cytokine secretion	1.358E-06	10/142
GO Biological Processes	GO:0050707	regulation of cytokine secretion	1.38E-05	11/225
GO Molecular Functions	GO:0019901	protein kinase binding	2.856E-29	49/654
GO Molecular Functions	GO:0019900	kinase binding	9.789E-29	51/741
GO Biological Processes	GO:0010942	positive regulation of cell death	8.619E-29	51/739

GO Biological Processes	GO:0043065	positive regulation of apoptotic process	1.189E-24	45/679
GO Biological Processes	GO:0043068	positive regulation of programmed cell death	2.043E-24	45/688
GO Biological Processes	GO:0051345	positive regulation of hydrolase activity	3.224E-17	39/777
GO Biological Processes	GO:0045862	positive regulation of proteolysis	4.712E-17	28/370
GO Biological Processes	GO:2000116	regulation of cysteine-type endopeptidase activity	2.191E-15	22/239
GO Biological Processes	GO:0052547	regulation of peptidase activity	1.21E-14	28/460
GO Biological Processes	GO:0052548	regulation of endopeptidase activity	1.659E-14	27/429
GO Biological Processes	GO:0043281	regulation of cysteine-type endopeptidase activity involved in apoptotic process	3.522E-14	20/215
GO Biological Processes	GO:0010952	positive regulation of peptidase activity	1.327E-11	17/200
GO Biological Processes	GO:2001056	positive regulation of cysteine-type endopeptidase activity	2.803E-10	14/151
GO Biological Processes	GO:0010950	positive regulation of endopeptidase activity	2.894E-10	15/180
GO Biological Processes	GO:0043280	positive regulation of cysteine-type endopeptidase activity involved in apoptotic process	8.036E-09	12/134
GO Biological Processes	GO:0006919	activation of cysteine-type endopeptidase activity involved in apoptotic process	2.166E-07	9/89
GO Biological Processes	GO:0070997	neuron death	2.376E-28	38/355
GO Biological Processes	GO:1901214	regulation of neuron death	2.954E-24	33/318
GO Biological Processes	GO:0051402	neuron apoptotic process	8.839E-21	27/243
GO Biological Processes	GO:0043523	regulation of neuron apoptotic process	1.42E-17	23/213
GO Biological Processes	GO:1901215	negative regulation of neuron death	2.827E-12	18/210
GO Biological Processes	GO:0043524	negative regulation of neuron apoptotic process	2.4E-09	13/148
GO Biological Processes	GO:0035690	cellular response to drug	2.005E-27	38/376
GO Biological Processes	GO:0000302	response to reactive oxygen species	5.675E-26	31/236
GO Biological Processes	GO:0006979	response to oxidative stress	2.391E-25	39/458
GO Biological Processes	GO:0009636	response to toxic substance	6.321E-24	40/533
GO Biological Processes	GO:0034614	cellular response to reactive oxygen species	1.141E-23	26/170
GO Biological Processes	GO:0034599	cellular response to oxidative stress	1.629E-23	32/309
GO Biological Processes	GO:0046677	response to antibiotic	1.705E-21	31/331
GO Biological Processes	GO:0097237	cellular response to toxic substance	6.549E-13	20/251
GO Biological Processes	GO:0042542	response to hydrogen peroxide	1.451E-11	15/146
GO Biological Processes	GO:0071236	cellular response to antibiotic	2.607E-09	13/149
GO Biological Processes	GO:0070301	cellular response to hydrogen peroxide	3.87E-09	11/100

GO Biological Processes	GO:0046777	protein autophosphorylation	2.719E-27	32/235
GO Molecular Functions	GO:0004713	protein tyrosine kinase activity	4.881E-26	26/139
GO Biological Processes	GO:0018108	peptidyl-tyrosine phosphorylation	2.18E-25	36/371
GO Biological Processes	GO:0018212	peptidyl-tyrosine modification	2.884E-25	36/374
GO Molecular Functions	GO:0004715	non-membrane spanning protein tyrosine kinase activity	8.385E-18	14/46
GO Biological Processes	GO:0038083	peptidyl-tyrosine autophosphorylation	2.833E-14	11/36
GO Biological Processes	GO:0050730	regulation of peptidyl-tyrosine phosphorylation	1.174E-12	20/259
GO Biological Processes	GO:0050731	positive regulation of peptidyl-tyrosine phosphorylation	8.488E-11	16/194
GO Molecular Functions	GO:0070851	growth factor receptor binding	7.975E-07	10/134
GO Biological Processes	GO:0061098	positive regulation of protein tyrosine kinase activity	1.166E-06	7/56
GO Biological Processes	GO:0061097	regulation of protein tyrosine kinase activity	2.554E-06	8/89
GO Biological Processes	GO:0097190	apoptotic signaling pathway	1.13E-26	45/607
GO Biological Processes	GO:2001233	regulation of apoptotic signaling pathway	7.301E-22	34/408
GO Biological Processes	GO:0097191	extrinsic apoptotic signaling pathway	1.008E-21	27/224
GO Biological Processes	GO:2001234	negative regulation of apoptotic signaling pathway	6.08E-19	25/233
GO Biological Processes	GO:2001237	negative regulation of extrinsic apoptotic signaling pathway	1.429E-17	18/106
GO Biological Processes	GO:2001236	regulation of extrinsic apoptotic signaling pathway	7.559E-17	20/157
GO Biological Processes	GO:0038034	signal transduction in absence of ligand	3.723E-16	15/73
GO Biological Processes	GO:0097192	extrinsic apoptotic signaling pathway in absence of ligand	3.723E-16	15/73
GO Biological Processes	GO:2001239	regulation of extrinsic apoptotic signaling pathway in absence of ligand	6.616E-10	9/47
GO Biological Processes	GO:2001240	negative regulation of extrinsic apoptotic signaling pathway in absence of ligand	4.951E-08	7/36
GO Biological Processes	GO:1901099	negative regulation of signal transduction in absence of ligand	4.951E-08	7/36
GO Biological Processes	GO:0030155	regulation of cell adhesion	1.608E-26	48/716
GO Biological Processes	GO:0022407	regulation of cell-cell adhesion	2.165E-19	32/422
GO Biological Processes	GO:0007159	leukocyte cell-cell adhesion	1.026E-17	28/349
GO Biological Processes	GO:0006909	phagocytosis	5.419E-17	28/372
GO Biological Processes	GO:0045785	positive regulation of cell adhesion	1.122E-16	29/416
GO Biological Processes	GO:0046649	lymphocyte activation	3.659E-16	37/748
GO Biological Processes	GO:0042110	T cell activation	3.973E-16	30/472
GO Biological Processes	GO:0002764	immune response-regulating signaling pathway	7.92E-16	35/681
GO Biological Processes	GO:1903037	regulation of leukocyte cell-cell adhesion	8.159E-16	25/315

GO Biological Processes	GO:0038093	Fc receptor signaling pathway	2.61E-15	22/241
GO Biological Processes	GO:0002757	immune response-activating signal transduction	7.135E-15	33/647
GO Biological Processes	GO:0002253	activation of immune response	3.885E-14	34/731
GO Biological Processes	GO:0032990	cell part morphogenesis	5.6E-14	33/696
GO Biological Processes	GO:0048812	neuron projection morphogenesis	7.016E-14	32/658
GO Biological Processes	GO:0000904	cell morphogenesis involved in differentiation	1.016E-13	34/756
GO Biological Processes	GO:0120039	plasma membrane bounded cell projection morphogenesis	1.244E-13	32/672
GO Biological Processes	GO:0050865	regulation of cell activation	1.425E-13	31/632
GO Biological Processes	GO:0048858	cell projection morphogenesis	1.462E-13	32/676
GO Biological Processes	GO:0002768	immune response-regulating cell surface receptor signaling pathway	1.479E-13	28/509
GO Biological Processes	GO:0061564	axon development	3.297E-13	28/526
GO Biological Processes	GO:0002429	immune response-activating cell surface receptor signaling pathway	1.363E-12	26/476
GO Biological Processes	GO:0022409	positive regulation of cell-cell adhesion	2.366E-12	20/269
GO Biological Processes	GO:0038096	Fc-gamma receptor signaling pathway involved in phagocytosis	7.093E-12	15/139
GO Biological Processes	GO:0002433	immune response-regulating cell surface receptor signaling pathway involved in phagocytosis	7.093E-12	15/139
GO Biological Processes	GO:0050863	regulation of T cell activation	7.583E-12	21/321
GO Biological Processes	GO:0038094	Fc-gamma receptor signaling pathway	9.687E-12	15/142
GO Biological Processes	GO:0007409	axonogenesis	9.692E-12	25/478
GO Biological Processes	GO:1903039	positive regulation of leukocyte cell-cell adhesion	9.759E-12	18/226
GO Biological Processes	GO:0002431	Fc receptor mediated stimulatory signaling pathway	1.187E-11	15/144
GO Biological Processes	GO:0048667	cell morphogenesis involved in neuron differentiation	3.958E-11	27/598
GO Biological Processes	GO:0051249	regulation of lymphocyte activation	1.562E-10	24/501
GO Biological Processes	GO:0002694	regulation of leukocyte activation	7.607E-10	25/588
GO Biological Processes	GO:0007411	axon guidance	2.497E-09	17/280
GO Biological Processes	GO:0097485	neuron projection guidance	2.78E-09	17/282
GO Biological Processes	GO:0038095	Fc-epsilon receptor signaling pathway	1.206E-08	13/169
GO Biological Processes	GO:0050870	positive regulation of T cell activation	1.603E-08	14/206
GO Biological Processes	GO:0031295	T cell costimulation	6.766E-08	8/56
GO Biological Processes	GO:0031294	lymphocyte costimulation	7.805E-08	8/57
GO Biological Processes	GO:0050867	positive regulation of cell activation	1.081E-07	18/406
GO Biological Processes	GO:0051251	positive regulation of lymphocyte activation	3.203E-07	16/346

GO Biological Processes	GO:0050851	antigen receptor-mediated signaling pathway	6.363E-07	15/320
GO Biological Processes	GO:0002696	positive regulation of leukocyte activation	1.604E-06	16/391
GO Biological Processes	GO:0050852	T cell receptor signaling pathway	4.977E-06	11/202
GO Biological Processes	GO:0006935	chemotaxis	1.961E-26	46/649
GO Biological Processes	GO:0042330	taxis	2.235E-26	46/651
GO Biological Processes	GO:0050900	leukocyte migration	7.311E-26	41/504
GO Biological Processes	GO:0032103	positive regulation of response to external stimulus	1.724E-16	26/325
GO Biological Processes	GO:0060326	cell chemotaxis	3.551E-16	25/304
GO Biological Processes	GO:0002685	regulation of leukocyte migration	1.877E-12	18/205
GO Biological Processes	GO:0050921	positive regulation of chemotaxis	5.737E-12	15/137
GO Biological Processes	GO:0050920	regulation of chemotaxis	6.208E-12	18/220
GO Biological Processes	GO:0030595	leukocyte chemotaxis	6.692E-10	16/223
GO Biological Processes	GO:0002687	positive regulation of leukocyte migration	7.692E-10	13/135
GO Biological Processes	GO:0097529	myeloid leukocyte migration	1.228E-06	12/212
GO Biological Processes	GO:0002688	regulation of leukocyte chemotaxis	1.953E-05	8/117
GO Biological Processes	GO:0002690	positive regulation of leukocyte chemotaxis	2.64E-05	7/89
GO Biological Processes	GO:0071677	positive regulation of mononuclear cell migration	6.685E-05	4/23
GO Biological Processes	GO:0071675	regulation of mononuclear cell migration	7.429E-05	5/45
GO Biological Processes	GO:0071674	mononuclear cell migration	0.0002823	6/92
GO Biological Processes	GO:0019221	cytokine-mediated signaling pathway	2.4E-26	50/796
