# Supplementary material for: Network Pharmacology Reveals That Resveratrol Can Alleviate COVID-19-Related Hyperinflammation
Source: Dis Markers. 2021 Sep 22;2021:4129993. doi: 10.1155/2021/4129993 (PMC8463930; doi:10.1155/2021/4129993)
Supplement: Supplementary 4 — Supplementary Table S4: SARS-CoV-2 DEGs GO analysis-enriched terms. [file 4129993.f4.pdf]

SARS-CoV-2 DEGs GO analysis enriched terms

Category	Term	Description	P	InTerm_InList
GO Biological Processes	GO:0019221	cytokine-mediated signaling pathway	2.051E-21	62/796
GO Molecular Functions	GO:0005125	cytokine activity	1.87E-16	29/219
GO Molecular Functions	GO:0048018	receptor ligand activity	1.086E-13	38/480
GO Molecular Functions	GO:0030545	receptor regulator activity	5.159E-13	39/530
GO Biological Processes	GO:0030593	neutrophil chemotaxis	5.769E-12	17/100
GO Biological Processes	GO:0060326	cell chemotaxis	5.88E-12	28/304
GO Biological Processes	GO:0030595	leukocyte chemotaxis	7.407E-12	24/223
GO Biological Processes	GO:1990266	neutrophil migration	7.932E-12	18/117
GO Biological Processes	GO:0071621	granulocyte chemotaxis	1.065E-11	18/119
GO Biological Processes	GO:0006935	chemotaxis	1.686E-11	41/649
GO Biological Processes	GO:0097529	myeloid leukocyte migration	1.727E-11	23/212
GO Biological Processes	GO:0042330	taxis	1.855E-11	41/651
GO Biological Processes	GO:0097530	granulocyte migration	2.02E-11	19/140
GO Molecular Functions	GO:0005126	cytokine receptor binding	3.81E-11	25/262
GO Biological Processes	GO:0006959	humoral immune response	2.875E-08	25/361
GO Biological Processes	GO:0019730	antimicrobial humoral response	8.869E-08	14/123
GO Biological Processes	GO:0061844	antimicrobial humoral immune response mediated by antimicrobial peptide	1.195E-07	11/73
GO Biological Processes	GO:0050900	leukocyte migration	1.283E-07	29/504
GO Molecular Functions	GO:0008009	chemokine activity	2.96E-07	9/49
GO Molecular Functions	GO:0045236	CXCR chemokine receptor binding	6.706E-07	6/18
GO Biological Processes	GO:1990868	response to chemokine	2.225E-06	11/97
GO Biological Processes	GO:1990869	cellular response to chemokine	2.225E-06	11/97
GO Molecular Functions	GO:0042379	chemokine receptor binding	5.849E-06	9/69
GO Biological Processes	GO:0070098	chemokine-mediated signaling pathway	6.316E-06	10/88
GO Molecular Functions	GO:0001664	G protein-coupled receptor binding	0.0012012	14/287
GO Biological Processes	GO:0032496	response to lipopolysaccharide	1.404E-11	29/338
GO Biological Processes	GO:0071222	cellular response to lipopolysaccharide	1.727E-11	23/212
GO Biological Processes	GO:0002237	response to molecule of bacterial origin	5.644E-11	29/358
GO Biological Processes	GO:0071219	cellular response to molecule of bacterial origin	5.833E-11	23/225
GO Biological Processes	GO:0071216	cellular response to biotic stimulus	4.442E-10	23/249
GO Biological Processes	GO:0009617	response to bacterium	6.205E-09	39/729
GO Biological Processes	GO:0071396	cellular response to lipid	2.885E-07	32/616
GO Biological Processes	GO:0071347	cellular response to interleukin-1	1.812E-06	15/180
GO Biological Processes	GO:0070555	response to interleukin-1	1.065E-05	15/208
GO Biological Processes	GO:0070498	interleukin-1-mediated signaling pathway	0.00062	8/100

GO Biological Processes	GO:0035690	cellular response to drug	0.0055559	15/376
GO Biological Processes	GO:0071407	cellular response to organic cyclic compound	0.0079701	19/544
GO Biological Processes	GO:0051607	defense response to virus	2.282E-09	22/248
GO Biological Processes	GO:0060337	type I interferon signaling pathway	3.037E-09	14/95
GO Biological Processes	GO:0071357	cellular response to type I interferon	3.037E-09	14/95
GO Biological Processes	GO:0034340	response to type I interferon	5.272E-09	14/99
GO Biological Processes	GO:0009615	response to virus	2.711E-08	24/334
GO Biological Processes	GO:0098542	defense response to other organism	4.263E-08	33/596
GO Biological Processes	GO:0034341	response to interferon-gamma	6.158E-08	18/202
GO Biological Processes	GO:0045071	negative regulation of viral genome replication	2.329E-07	10/62
GO Biological Processes	GO:0045069	regulation of viral genome replication	3.716E-07	12/99
GO Biological Processes	GO:1903901	negative regulation of viral life cycle	7.418E-07	11/87
GO Biological Processes	GO:0048525	negative regulation of viral process	3.662E-06	11/102
GO Biological Processes	GO:1903900	regulation of viral life cycle	5.32E-06	13/149
GO Biological Processes	GO:0019079	viral genome replication	5.372E-06	12/127
GO Biological Processes	GO:0071346	cellular response to interferon-gamma	1.006E-05	14/182
GO Molecular Functions	GO:0001730	2'-5'-oligoadenylate synthetase activity	2.636E-05	3/4
GO Biological Processes	GO:0043903	regulation of symbiosis, encompassing mutualism through parasitism	2.693E-05	15/225
GO Biological Processes	GO:0050792	regulation of viral process	4.712E-05	14/209
GO Biological Processes	GO:0043900	regulation of multi-organism process	0.0001255	20/412
GO Biological Processes	GO:0043901	negative regulation of multi-organism process	0.000137	12/176
GO Biological Processes	GO:0060333	interferon-gamma-mediated signaling pathway	0.000328	8/91
GO Biological Processes	GO:0060700	regulation of ribonuclease activity	0.0005158	3/9
GO Biological Processes	GO:0019058	viral life cycle	0.0005896	16/330
GO Biological Processes	GO:0032069	regulation of nuclease activity	0.0078818	3/22
GO Biological Processes	GO:2000147	positive regulation of cell motility	2.633E-08	33/584
GO Biological Processes	GO:0030335	positive regulation of cell migration	3.323E-08	32/560
GO Biological Processes	GO:0051272	positive regulation of cellular component movement	5.189E-08	33/601
GO Biological Processes	GO:0040017	positive regulation of locomotion	7.928E-08	33/612
GO Biological Processes	GO:0001568	blood vessel development	8.355E-05	31/777
GO Biological Processes	GO:1904018	positive regulation of vasculature development	0.0001503	14/233
GO Biological Processes	GO:0001525	angiogenesis	0.0004367	24/594
GO Biological Processes	GO:0010634	positive regulation of epithelial cell migration	0.0005163	11/175
GO Biological Processes	GO:0045766	positive regulation of angiogenesis	0.0005785	12/206
GO Biological Processes	GO:0048514	blood vessel morphogenesis	0.0007245	26/690
GO Biological Processes	GO:0010595	positive regulation of endothelial cell migration	0.0008739	9/131

GO Biological Processes	GO:1901342	regulation of vasculature development	0.0016541	18/434
GO Biological Processes	GO:0043536	positive regulation of blood vessel endothelial cell migration	0.0040573	6/80
GO Biological Processes	GO:0045765	regulation of angiogenesis	0.0080195	15/392
GO Biological Processes	GO:0007159	leukocyte cell-cell adhesion	6.226E-08	24/349
GO Biological Processes	GO:0045785	positive regulation of cell adhesion	1.504E-06	24/416
GO Biological Processes	GO:0030155	regulation of cell adhesion	2.633E-06	33/716
GO Biological Processes	GO:1903037	regulation of leukocyte cell-cell adhesion	2.694E-06	20/315
GO Biological Processes	GO:0022407	regulation of cell-cell adhesion	6.367E-06	23/422
GO Biological Processes	GO:1903039	positive regulation of leukocyte cell-cell adhesion	6.88E-06	16/226
GO Biological Processes	GO:1903706	regulation of hemopoiesis	7.189E-06	25/487
GO Biological Processes	GO:1902105	regulation of leukocyte differentiation	7.499E-06	18/281
GO Biological Processes	GO:0002521	leukocyte differentiation	8.06E-06	26/522
GO Biological Processes	GO:0050865	regulation of cell activation	1.165E-05	29/632
GO Biological Processes	GO:0050863	regulation of T cell activation	1.296E-05	19/321
GO Biological Processes	GO:0022409	positive regulation of cell-cell adhesion	1.594E-05	17/269
GO Biological Processes	GO:0045619	regulation of lymphocyte differentiation	2.503E-05	13/172
GO Biological Processes	GO:0033089	positive regulation of T cell differentiation in thymus	5.501E-05	4/12
GO Biological Processes	GO:0002694	regulation of leukocyte activation	6.092E-05	26/588
GO Biological Processes	GO:0045580	regulation of T cell differentiation	8.896E-05	11/143
GO Biological Processes	GO:0051249	regulation of lymphocyte activation	9.255E-05	23/501
GO Biological Processes	GO:0042110	T cell activation	0.0001054	22/472
GO Biological Processes	GO:0033081	regulation of T cell differentiation in thymus	0.0001112	5/26
GO Biological Processes	GO:0050870	positive regulation of T cell activation	0.0001586	13/206
GO Biological Processes	GO:0046649	lymphocyte activation	0.0002282	29/748
GO Biological Processes	GO:1903708	positive regulation of hemopoiesis	0.0003704	12/196
GO Biological Processes	GO:0045582	positive regulation of T cell differentiation	0.0012156	7/86
GO Biological Processes	GO:0030217	T cell differentiation	0.0023748	12/243
GO Biological Processes	GO:0045621	positive regulation of lymphocyte differentiation	0.0024363	7/97
GO Biological Processes	GO:1902107	positive regulation of leukocyte differentiation	0.0024546	9/152
GO Biological Processes	GO:0030098	lymphocyte differentiation	0.0032892	15/355
GO Biological Processes	GO:0050867	positive regulation of cell activation	0.0047865	16/406
GO Biological Processes	GO:0002696	positive regulation of leukocyte activation	0.0078437	15/391
GO Biological Processes	GO:0032103	positive regulation of response to external stimulus	2.902E-07	22/325

GO Biological Processes	GO:0050727	regulation of inflammatory response	5.044E-07	28/508
GO Biological Processes	GO:0051091	positive regulation of DNA-binding transcription factor activity	8.397E-07	19/266
GO Biological Processes	GO:0050729	positive regulation of inflammatory response	1.123E-06	14/151
GO Biological Processes	GO:0051092	positive regulation of NF-kappaB transcription factor activity	1.316E-06	14/153
GO Biological Processes	GO:0038061	NIK/NF-kappaB signaling	2.914E-06	15/187
GO Biological Processes	GO:1901222	regulation of NIK/NF-kappaB signaling	2.966E-06	12/120
GO Biological Processes	GO:0051090	regulation of DNA-binding transcription factor activity	1.249E-05	23/440
GO Biological Processes	GO:1901224	positive regulation of NIK/NF-kappaB signaling	1.62E-05	9/78
GO Biological Processes	GO:0007249	I-kappaB kinase/NF-kappaB signaling	0.0002965	15/279
GO Biological Processes	GO:0043122	regulation of I-kappaB kinase/NF-kappaB signaling	0.0008318	13/245
GO Biological Processes	GO:0043123	positive regulation of I-kappaB kinase/NF-kappaB signaling	0.0087695	9/185
GO Biological Processes	GO:0050730	regulation of peptidyl-tyrosine phosphorylation	5.606E-07	19/259
GO Biological Processes	GO:0050731	positive regulation of peptidyl-tyrosine phosphorylation	9.452E-07	16/194
GO Biological Processes	GO:0018108	peptidyl-tyrosine phosphorylation	2.969E-05	20/371
GO Biological Processes	GO:0018212	peptidyl-tyrosine modification	3.326E-05	20/374
GO Biological Processes	GO:0042531	positive regulation of tyrosine phosphorylation of STAT protein	6.968E-05	8/73
GO Biological Processes	GO:0002573	myeloid leukocyte differentiation	0.0001745	13/208
GO Biological Processes	GO:0042509	regulation of tyrosine phosphorylation of STAT protein	0.0002049	8/85
GO Biological Processes	GO:0007259	JAK-STAT cascade	0.0002402	11/160
GO Biological Processes	GO:0007260	tyrosine phosphorylation of STAT protein	0.0002605	8/88
GO Biological Processes	GO:0046427	positive regulation of JAK-STAT cascade	0.0003534	8/92
GO Biological Processes	GO:0046425	regulation of JAK-STAT cascade	0.0003564	10/141
GO Biological Processes	GO:0097696	STAT cascade	0.0004041	11/170
GO Biological Processes	GO:1904894	positive regulation of STAT cascade	0.0004394	8/95
GO Biological Processes	GO:1904892	regulation of STAT cascade	0.0005812	10/150
GO Molecular Functions	GO:0008083	growth factor activity	0.0010522	10/162
GO Biological Processes	GO:0001892	embryonic placenta development	0.00169	7/91
GO Biological Processes	GO:0001890	placenta development	0.0030502	9/157
GO Biological Processes	GO:0045637	regulation of myeloid cell differentiation	0.0038548	12/258
GO Biological Processes	GO:0030099	myeloid cell differentiation	0.0070043	16/423
GO Biological Processes	GO:0002761	regulation of myeloid leukocyte differentiation	0.0078169	7/120
GO Biological Processes	GO:0071356	cellular response to tumor necrosis factor	8.835E-07	20/293
GO Biological Processes	GO:0034612	response to tumor necrosis factor	2.694E-06	20/315

GO Biological Processes	GO:0033209	tumor necrosis factor-mediated signaling pathway	0.0003844	11/169
GO Molecular Functions	GO:0032813	tumor necrosis factor receptor superfamily binding	0.0015388	5/45
GO Molecular Functions	GO:0005164	tumor necrosis factor receptor binding	0.0026334	4/31
GO Biological Processes	GO:0002526	acute inflammatory response	1.223E-06	17/222
GO Biological Processes	GO:0006953	acute-phase response	2.501E-06	8/47
GO Biological Processes	GO:0002675	positive regulation of acute inflammatory response	2.16E-05	6/31
GO Biological Processes	GO:2000379	positive regulation of reactive oxygen species metabolic process	2.568E-05	10/103
GO Biological Processes	GO:1903428	positive regulation of reactive oxygen species biosynthetic process	9.55E-05	7/57
GO Biological Processes	GO:0072593	reactive oxygen species metabolic process	0.0001195	16/286
GO Biological Processes	GO:0034113	heterotypic cell-cell adhesion	0.0001814	7/63
GO Biological Processes	GO:0060558	regulation of calcidiol 1-monooxygenase activity	0.0002211	3/7
GO Biological Processes	GO:0032602	chemokine production	0.0003534	8/92
GO Biological Processes	GO:0060556	regulation of vitamin D biosynthetic process	0.0005158	3/9
GO Biological Processes	GO:0150076	neuroinflammatory response	0.000581	7/76
GO Biological Processes	GO:0001660	fever generation	0.0007265	3/10
GO Biological Processes	GO:1903426	regulation of reactive oxygen species biosynthetic process	0.000804	8/104
GO Biological Processes	GO:0046209	nitric oxide metabolic process	0.0009172	7/82
GO Biological Processes	GO:0046886	positive regulation of hormone biosynthetic process	0.000985	3/11
GO Biological Processes	GO:0032350	regulation of hormone metabolic process	0.0010039	5/41
GO Biological Processes	GO:0072604	interleukin-6 secretion	0.0010112	6/61
GO Biological Processes	GO:0150077	regulation of neuroinflammatory response	0.0011221	5/42
GO Biological Processes	GO:2001057	reactive nitrogen species metabolic process	0.0011348	7/85
GO Biological Processes	GO:0045429	positive regulation of nitric oxide biosynthetic process	0.0012503	5/43
GO Biological Processes	GO:0001505	regulation of neurotransmitter levels	0.0012719	16/355
GO Biological Processes	GO:0042368	vitamin D biosynthetic process	0.001295	3/12
GO Biological Processes	GO:2000377	regulation of reactive oxygen species metabolic process	0.0013641	11/197
GO Biological Processes	GO:1904407	positive regulation of nitric oxide metabolic process	0.0013891	5/44
GO Biological Processes	GO:0045428	regulation of nitric oxide biosynthetic process	0.0015291	6/66
GO Biological Processes	GO:0030656	regulation of vitamin metabolic process	0.00166	3/13
GO Biological Processes	GO:0046885	regulation of hormone biosynthetic process	0.0017916	4/28
GO Biological Processes	GO:0010883	regulation of lipid storage	0.0022583	5/49
GO Biological Processes	GO:0019915	lipid storage	0.0025657	6/73
GO Biological Processes	GO:0042362	fat-soluble vitamin biosynthetic process	0.0025679	3/15

GO Biological Processes	GO:0045073	regulation of chemokine biosynthetic process	0.0025679	3/15
GO Biological Processes	GO:0051044	positive regulation of membrane protein ectodomain proteolysis	0.0025679	3/15
GO Biological Processes	GO:1903409	reactive oxygen species biosynthetic process	0.0026092	8/125
GO Biological Processes	GO:0006801	superoxide metabolic process	0.0027485	6/74
GO Biological Processes	GO:0032352	positive regulation of hormone metabolic process	0.0031165	3/16
GO Biological Processes	GO:0034116	positive regulation of heterotypic cell-cell adhesion	0.0031165	3/16
GO Biological Processes	GO:0042033	chemokine biosynthetic process	0.0031165	3/16
GO Biological Processes	GO:0050755	chemokine metabolic process	0.0031165	3/16
GO Biological Processes	GO:0014015	positive regulation of gliogenesis	0.0031431	6/76
GO Biological Processes	GO:1904994	regulation of leukocyte adhesion to vascular endothelial cell	0.0033246	4/33
GO Biological Processes	GO:0006809	nitric oxide biosynthetic process	0.0033556	6/77
GO Biological Processes	GO:0032675	regulation of interleukin-6 production	0.0034588	9/160
GO Biological Processes	GO:0002673	regulation of acute inflammatory response	0.0036042	9/161
GO Biological Processes	GO:0031649	heat generation	0.0037317	3/17
GO Biological Processes	GO:0010001	glial cell differentiation	0.0038284	11/225
GO Biological Processes	GO:0051353	positive regulation of oxidoreductase activity	0.0043859	5/57
GO Biological Processes	GO:0150078	positive regulation of neuroinflammatory response	0.0044159	3/18
GO Biological Processes	GO:2000778	positive regulation of interleukin-6 secretion	0.004578	4/36
GO Biological Processes	GO:0032722	positive regulation of chemokine production	0.005086	5/59
GO Biological Processes	GO:0061900	glial cell activation	0.005086	5/59
GO Biological Processes	GO:0032635	interleukin-6 production	0.0051391	9/170
GO Biological Processes	GO:0002922	positive regulation of humoral immune response	0.0051712	3/19
GO Biological Processes	GO:0060252	positive regulation of glial cell proliferation	0.0051712	3/19
GO Biological Processes	GO:0032642	regulation of chemokine production	0.00546	6/85
GO Biological Processes	GO:0010829	negative regulation of glucose transmembrane transport	0.0069025	3/21
GO Biological Processes	GO:0010888	negative regulation of lipid storage	0.0069025	3/21
GO Biological Processes	GO:0010893	positive regulation of steroid biosynthetic process	0.0069025	3/21
GO Biological Processes	GO:1904996	positive regulation of leukocyte adhesion to vascular endothelial cell	0.0069025	3/21
GO Biological Processes	GO:1905953	negative regulation of lipid localization	0.0071762	5/64
GO Biological Processes	GO:0021782	glial cell development	0.0074789	7/119
GO Biological Processes	GO:0042359	vitamin D metabolic process	0.0078818	3/22
GO Biological Processes	GO:0032755	positive regulation of interleukin-6 production	0.0079792	6/92
GO Biological Processes	GO:0042133	neurotransmitter metabolic process	0.0084036	8/152

GO Biological Processes	GO:0051043	regulation of membrane protein ectodomain proteolysis	0.0089389	3/23
GO Biological Processes	GO:0045123	cellular extravasation	0.0092309	5/68
GO Biological Processes	GO:0014002	astrocyte development	0.0093768	4/44
GO Biological Processes	GO:0042116	macrophage activation	0.0097461	6/96
GO Biological Processes	GO:0042108	positive regulation of cytokine biosynthetic process	0.0098017	5/69
GO Molecular Functions	GO:0017159	pantetheine hydrolase activity	6.684E-06	3/3
GO Biological Processes	GO:0015939	pantothenate metabolic process	0.0002211	3/7
GO Cellular Components	GO:0031225	anchored component of membrane	0.0003473	11/167
GO Biological Processes	GO:0006575	cellular modified amino acid metabolic process	0.0063885	10/208
GO Biological Processes	GO:0043410	positive regulation of MAPK cascade	7.507E-06	27/552
GO Biological Processes	GO:0043408	regulation of MAPK cascade	0.0002604	29/754
GO Biological Processes	GO:0070372	regulation of ERK1 and ERK2 cascade	0.0002771	16/308
GO Biological Processes	GO:0070374	positive regulation of ERK1 and ERK2 cascade	0.0002887	13/219
GO Biological Processes	GO:0070371	ERK1 and ERK2 cascade	0.0005522	16/328
GO Biological Processes	GO:0045860	positive regulation of protein kinase activity	0.0034865	20/540
GO Biological Processes	GO:0043406	positive regulation of MAP kinase activity	0.0044872	12/263
GO Biological Processes	GO:0071902	positive regulation of protein serine/threonine kinase activity	0.0059758	14/343
GO Biological Processes	GO:0052548	regulation of endopeptidase activity	8.317E-06	23/429
GO Biological Processes	GO:0052547	regulation of peptidase activity	2.521E-05	23/460
GO Biological Processes	GO:2000116	regulation of cysteine-type endopeptidase activity	0.0001958	14/239
GO Biological Processes	GO:0043281	regulation of cysteine-type endopeptidase activity involved in apoptotic process	0.0008425	12/215
GO Biological Processes	GO:0006919	activation of cysteine-type endopeptidase activity involved in apoptotic process	0.0068146	6/89
GO Biological Processes	GO:0010950	positive regulation of endopeptidase activity	0.0073925	9/180
GO Biological Processes	GO:0010942	positive regulation of cell death	0.0074426	24/739
GO Biological Processes	GO:2001056	positive regulation of cysteine-type endopeptidase activity	0.0080905	8/151
GO Biological Processes	GO:0002274	myeloid leukocyte activation	8.78E-06	30/656
GO Biological Processes	GO:0002263	cell activation involved in immune response	1.766E-05	31/715
GO Biological Processes	GO:0042119	neutrophil activation	3.356E-05	24/501
GO Biological Processes	GO:0002446	neutrophil mediated immunity	3.464E-05	24/502
GO Biological Processes	GO:0002366	leukocyte activation involved in immune response	3.985E-05	30/711
GO Biological Processes	GO:0036230	granulocyte activation	4.053E-05	24/507
GO Biological Processes	GO:0002444	myeloid leukocyte mediated immunity	6.274E-05	25/555
GO Biological Processes	GO:0045055	regulated exocytosis	0.0001289	31/796

GO Biological Processes	GO:0043312	neutrophil degranulation	0.0001692	22/488
GO Biological Processes	GO:0002283	neutrophil activation involved in immune response	0.0001844	22/491
GO Biological Processes	GO:0043299	leukocyte degranulation	0.0002537	23/537
GO Biological Processes	GO:0002275	myeloid cell activation involved in immune response	0.0003292	23/547
GO Cellular Components	GO:0030667	secretory granule membrane	0.00529	13/303
GO Biological Processes	GO:0070268	cornification	9.926E-06	11/113
GO Biological Processes	GO:0043588	skin development	0.0001522	20/418
GO Biological Processes	GO:0030216	keratinocyte differentiation	0.0002223	16/302
GO Biological Processes	GO:0030855	epithelial cell differentiation	0.0005103	29/786
GO Biological Processes	GO:0009913	epidermal cell differentiation	0.0005417	17/360
GO Biological Processes	GO:0008544	epidermis development	0.0006657	20/469
GO Biological Processes	GO:0031424	keratinization	0.0012466	12/225
GO Cellular Components	GO:0001533	cornified envelope	0.0015388	5/45
GO Biological Processes	GO:0002687	positive regulation of leukocyte migration	1.009E-05	12/135
GO Biological Processes	GO:0002685	regulation of leukocyte migration	3.815E-05	14/205
GO Biological Processes	GO:0048247	lymphocyte chemotaxis	0.0002209	7/65
GO Biological Processes	GO:0072676	lymphocyte migration	0.0003609	9/116
GO Biological Processes	GO:2000403	positive regulation of lymphocyte migration	0.0007043	5/38
GO Biological Processes	GO:0050920	regulation of chemotaxis	0.0010281	12/220
GO Biological Processes	GO:0050921	positive regulation of chemotaxis	0.0011997	9/137
GO Biological Processes	GO:2000401	regulation of lymphocyte migration	0.0013022	6/64
GO Biological Processes	GO:2000404	regulation of T cell migration	0.0013891	5/44
GO Biological Processes	GO:0002690	positive regulation of leukocyte chemotaxis	0.0014855	7/89
GO Biological Processes	GO:0072678	T cell migration	0.0016532	6/67
GO Biological Processes	GO:0071624	positive regulation of granulocyte chemotaxis	0.0017916	4/28
GO Biological Processes	GO:1902624	positive regulation of neutrophil migration	0.0026334	4/31
GO Biological Processes	GO:2000406	positive regulation of T cell migration	0.0026334	4/31
GO Biological Processes	GO:0002688	regulation of leukocyte chemotaxis	0.0068353	7/117
GO Biological Processes	GO:0140131	positive regulation of lymphocyte chemotaxis	0.0069025	3/21
GO Biological Processes	GO:1902622	regulation of neutrophil migration	0.0079622	4/42
GO Biological Processes	GO:0045124	regulation of bone resorption	1.243E-05	7/42
GO Biological Processes	GO:0046850	regulation of bone remodeling	3.533E-05	7/49
GO Biological Processes	GO:0048871	multicellular organismal homeostasis	6.053E-05	23/487
GO Biological Processes	GO:0001894	tissue homeostasis	0.0001143	14/227
GO Biological Processes	GO:0045453	bone resorption	0.0001814	7/63
GO Biological Processes	GO:0034103	regulation of tissue remodeling	0.000328	8/91

GO Biological Processes	GO:0046849	bone remodeling	0.000328	8/91
GO Biological Processes	GO:0048771	tissue remodeling	0.000684	11/181
GO Biological Processes	GO:0060249	anatomical structure homeostasis	0.0017833	18/437
GO Biological Processes	GO:0030225	macrophage differentiation	1.709E-05	7/44
GO Biological Processes	GO:0097154	GABAergic neuron differentiation	0.000108	4/14
GO Biological Processes	GO:0021879	forebrain neuron differentiation	0.004063	5/56
GO Biological Processes	GO:0045649	regulation of macrophage differentiation	0.0078818	3/22
GO Biological Processes	GO:0045746	negative regulation of Notch signaling pathway	0.0093768	4/44
GO Biological Processes	GO:0021872	forebrain generation of neurons	0.0098017	5/69
GO Biological Processes	GO:0048661	positive regulation of smooth muscle cell proliferation	2.162E-05	10/101
GO Biological Processes	GO:0048660	regulation of smooth muscle cell proliferation	9.324E-05	12/169
GO Biological Processes	GO:0048659	smooth muscle cell proliferation	0.0001043	12/171
GO Biological Processes	GO:1904707	positive regulation of vascular smooth muscle cell proliferation	0.000308	6/49
GO Biological Processes	GO:1904705	regulation of vascular smooth muscle cell proliferation	0.0011348	7/85
GO Biological Processes	GO:1990874	vascular smooth muscle cell proliferation	0.0011348	7/85
GO Biological Processes	GO:0033002	muscle cell proliferation	0.0021437	12/240
GO Biological Processes	GO:0071398	cellular response to fatty acid	0.0043859	5/57
GO Biological Processes	GO:0000302	response to reactive oxygen species	0.0054567	11/236
GO Biological Processes	GO:0070542	response to fatty acid	0.00611	6/87
GO Biological Processes	GO:0097237	cellular response to toxic substance	0.0085069	11/251
GO Biological Processes	GO:0030949	positive regulation of vascular endothelial growth factor receptor signaling pathway	2.191E-05	5/19
GO Biological Processes	GO:0030947	regulation of vascular endothelial growth factor receptor signaling pathway	0.0005456	5/36
GO Biological Processes	GO:0048010	vascular endothelial growth factor receptor signaling pathway	0.0097461	6/96
GO Biological Processes	GO:0001819	positive regulation of cytokine production	3.188E-05	23/467
GO Biological Processes	GO:0051047	positive regulation of secretion	3.288E-05	22/436
GO Biological Processes	GO:1903532	positive regulation of secretion by cell	0.0001031	20/406
GO Biological Processes	GO:0050663	cytokine secretion	0.0001116	15/255
GO Biological Processes	GO:0001817	regulation of cytokine production	0.0001166	30/755
GO Biological Processes	GO:0050707	regulation of cytokine secretion	0.0003743	13/225
GO Biological Processes	GO:0050715	positive regulation of cytokine secretion	0.000377	10/142
GO Biological Processes	GO:1903530	regulation of secretion by cell	0.0004245	29/777
GO Biological Processes	GO:0002793	positive regulation of peptide secretion	0.0005323	15/295
GO Biological Processes	GO:0051051	negative regulation of transport	0.0007211	22/543
GO Biological Processes	GO:0050714	positive regulation of protein secretion	0.0007702	14/274

GO Biological Processes	GO:0002790	peptide secretion	0.0031185	23/649
GO Biological Processes	GO:1903531	negative regulation of secretion by cell	0.0033433	11/221
GO Biological Processes	GO:0002791	regulation of peptide secretion	0.0050166	19/520
GO Biological Processes	GO:0050708	regulation of protein secretion	0.0061385	18/492
GO Biological Processes	GO:0009306	protein secretion	0.0068481	21/614
GO Biological Processes	GO:0046888	negative regulation of hormone secretion	0.0071762	5/64
GO Biological Processes	GO:0051222	positive regulation of protein transport	0.0074702	16/426
GO Biological Processes	GO:0051048	negative regulation of secretion	0.0075884	11/247
GO Biological Processes	GO:0050709	negative regulation of protein secretion	0.0084036	8/152
