# Supplementary material for: Network Pharmacology Reveals That Resveratrol Can Alleviate COVID-19-Related Hyperinflammation
Source: Dis Markers. 2021 Sep 22;2021:4129993. doi: 10.1155/2021/4129993 (PMC8463930; doi:10.1155/2021/4129993)
Supplement: Supplementary 5 — Supplementary Table S5: resveratrol-related targets KEGG-enriched terms. [file 4129993.f5.pdf]

targets KEGG enriched terms

Category	Term	Description	P	InTerm_InList
KEGG Pathway	hsa05200	Pathways in cancer	1.3124E-71	72/395
KEGG Pathway	hsa04151	PI3K-Akt signaling pathway	8.6568E-43	49/342
KEGG Pathway	hsa05205	Proteoglycans in cancer	3.5979E-42	41/203
KEGG Pathway	hsa05215	Prostate cancer	7.8907E-39	30/87
KEGG Pathway	hsa04510	Focal adhesion	1.1767E-36	37/199
KEGG Pathway	hsa01522	Endocrine resistance	1.3317E-35	29/96
KEGG Pathway	hsa01521	EGFR tyrosine kinase inhibitor resistance	6.7344E-35	27/79
KEGG Pathway	hsa04210	Apoptosis	1.6551E-30	29/138
KEGG Pathway	hsa04919	Thyroid hormone signaling pathway	9.1582E-30	27/116
KEGG Pathway	hsa04062	Chemokine signaling pathway	1.4712E-29	31/182
KEGG Pathway	hsa05212	Pancreatic cancer	1.1241E-28	22/64
KEGG Pathway	hsa05161	Hepatitis B	1.7959E-28	28/144
KEGG Pathway	hsa04015	Rap1 signaling pathway	1.4426E-27	31/210
KEGG Pathway	hsa04014	Ras signaling pathway	1.6811E-26	31/227
KEGG Pathway	hsa05145	Toxoplasmosis	1.5921E-25	24/113
KEGG Pathway	hsa04012	ErbB signaling pathway	1.9717E-25	22/86
KEGG Pathway	hsa04664	Fc epsilon RI signaling pathway	1.3796E-24	20/68
KEGG Pathway	hsa04917	Prolactin signaling pathway	2.6737E-24	20/70
KEGG Pathway	hsa04370	VEGF signaling pathway	2.8064E-24	19/59
KEGG Pathway	hsa04914	Progesterone-mediated oocyte maturation	2.8439E-24	22/96
KEGG Pathway	hsa05222	Small cell lung cancer	4.3139E-24	21/84
KEGG Pathway	hsa04380	Osteoclast differentiation	5.7563E-24	24/130
KEGG Pathway	hsa04810	Regulation of actin cytoskeleton	1.3214E-23	28/212
KEGG Pathway	hsa05214	Glioma	1.6781E-23	19/64
KEGG Pathway	hsa04722	Neurotrophin signaling pathway	1.7012E-23	23/119
KEGG Pathway	hsa04910	Insulin signaling pathway	2.5936E-23	24/138
KEGG Pathway	hsa05221	Acute myeloid leukemia	3.3007E-23	18/55
KEGG Pathway	hsa04668	TNF signaling pathway	4.684E-23	22/108
KEGG Pathway	hsa05223	Non-small cell lung cancer	1.0047E-22	18/58
KEGG Pathway	hsa05220	Chronic myeloid leukemia	1.5713E-22	19/71
KEGG Pathway	hsa04066	HIF-1 signaling pathway	2.9408E-22	21/101
KEGG Pathway	hsa05160	Hepatitis C	4.1342E-21	22/131
KEGG Pathway	hsa04915	Estrogen signaling pathway	4.4505E-21	20/98
KEGG Pathway	hsa04068	FoxO signaling pathway	4.919E-21	22/132
KEGG Pathway	hsa05231	Choline metabolism in cancer	5.5298E-21	20/99
KEGG Pathway	hsa05210	Colorectal cancer	9.3259E-21	17/60
KEGG Pathway	hsa04660	T cell receptor signaling pathway	1.2858E-20	20/103
KEGG Pathway	hsa04010	MAPK signaling pathway	3.1597E-20	27/255
KEGG Pathway	hsa05230	Central carbon metabolism in cancer	4.31E-20	17/65
KEGG Pathway	hsa05218	Melanoma	1.3299E-19	17/69
KEGG Pathway	hsa05213	Endometrial cancer	7.019E-19	15/50
KEGG Pathway	hsa05224	Breast cancer	7.0741E-19	21/144
KEGG Pathway	hsa04140	Autophagy - animal	1.2048E-18	20/128
KEGG Pathway	hsa05164	Influenza A	2.1103E-18	22/173
KEGG Pathway	hsa05169	Epstein-Barr virus infection	3.8377E-18	23/201
KEGG Pathway	hsa05142	Chagas disease (American trypanosomiasis)	6.9415E-18	18/102
KEGG Pathway	hsa04662	B cell receptor signaling pathway	7.6652E-18	16/71
KEGG Pathway	hsa04932	Non-alcoholic fatty liver disease (NAFLD)	2.6486E-17	20/149
KEGG Pathway	hsa05152	Tuberculosis	6.9179E-17	21/179
KEGG Pathway	hsa05166	HTLV-I infection	7.2444E-17	24/256
KEGG Pathway	hsa01524	Platinum drug resistance	3.7227E-16	15/73
KEGG Pathway	hsa04666	Fc gamma R-mediated phagocytosis	5.2527E-16	16/91
KEGG Pathway	hsa04150	mTOR signaling pathway	5.8006E-16	19/151
KEGG Pathway	hsa05211	Renal cell carcinoma	1.8112E-15	14/65
KEGG Pathway	hsa04620	Toll-like receptor signaling pathway	4.787E-15	16/104
KEGG Pathway	hsa04072	Phospholipase D signaling pathway	5.0015E-15	18/146

KEGG Pathway	hsa04550	Signaling pathways regulating pluripotency of stem cells	7.0926E-12	15/139
KEGG Pathway	hsa05162	Measles	5.6143E-11	14/134
KEGG Pathway	hsa04630	Jak-STAT signaling pathway	4.3252E-10	14/156
KEGG Pathway	hsa04933	AGE-RAGE signaling pathway in diabetic complications	3.7218E-35	29/99
KEGG Pathway	hsa05418	Fluid shear stress and atherosclerosis	1.183E-28	28/142
KEGG Pathway	hsa04931	Insulin resistance	1.2398E-24	23/107
KEGG Pathway	hsa04920	Adipocytokine signaling pathway	4.4362E-15	14/69
KEGG Pathway	hsa04930	Type II diabetes mellitus	1.8805E-11	10/46
KEGG Pathway	hsa04071	Sphingolipid signaling pathway	1.5182E-29	27/118
KEGG Pathway	hsa04024	cAMP signaling pathway	9.7317E-26	29/198
KEGG Pathway	hsa04611	Platelet activation	9.7184E-22	22/123
KEGG Pathway	hsa04725	Cholinergic synapse	1.7916E-18	19/112
KEGG Pathway	hsa04371	Apelin signaling pathway	1.0428E-16	19/138
KEGG Pathway	hsa04022	cGMP-PKG signaling pathway	4.7757E-13	17/163
KEGG Pathway	hsa04261	Adrenergic signaling in cardiomyocytes	1.7561E-07	11/144
KEGG Pathway	hsa05206	MicroRNAs in cancer	8.0957E-23	31/299
KEGG Pathway	hsa05203	Viral carcinogenesis	2.5324E-19	24/201
KEGG Pathway	hsa04110	Cell cycle	1.3181E-12	15/124
KEGG Pathway	hsa04115	p53 signaling pathway	1.2903E-09	10/69
KEGG Pathway	hsa04080	Neuroactive ligand-receptor interaction	2.7581E-19	27/277
KEGG Pathway	hsa04020	Calcium signaling pathway	3.9835E-19	23/182
KEGG Pathway	hsa04152	AMPK signaling pathway	6.9564E-18	19/120
KEGG Pathway	hsa04923	Regulation of lipolysis in adipocytes	3.9005E-15	13/54
KEGG Pathway	hsa04213	Longevity regulating pathway - multiple species	7.6262E-13	12/62
KEGG Pathway	hsa04211	Longevity regulating pathway	6.6521E-11	12/89
KEGG Pathway	hsa04922	Glucagon signaling pathway	6.709E-08	10/103
KEGG Pathway	hsa04973	Carbohydrate digestion and absorption	2.1275E-07	7/44
KEGG Pathway	hsa04657	IL-17 signaling pathway	3.1307E-17	17/93
KEGG Pathway	hsa04659	Th17 cell differentiation	7.6224E-15	16/107
KEGG Pathway	hsa05131	Shigellosis	5.2597E-14	13/65
KEGG Pathway	hsa05120	Epithelial cell signaling in Helicobacter pylori infection	9.7925E-14	13/68
KEGG Pathway	hsa04621	NOD-like receptor signaling pathway	9.5363E-13	17/170
KEGG Pathway	hsa05133	Pertussis	1.92E-10	11/76
KEGG Pathway	hsa05132	Salmonella infection	7.5312E-10	11/86
KEGG Pathway	hsa05140	Leishmaniasis	2.279E-09	10/73
KEGG Pathway	hsa04530	Tight junction	1.1466E-07	12/170
KEGG Pathway	hsa04658	Th1 and Th2 cell differentiation	2.8861E-07	9/92
KEGG Pathway	hsa05168	Herpes simplex infection	2.1305E-06	11/185
KEGG Pathway	hsa04622	RIG-I-like receptor signaling pathway	5.3701E-06	7/70
KEGG Pathway	hsa04137	Mitophagy - animal	0.00042893	5/65
KEGG Pathway	hsa04726	Serotonergic synapse	4.7037E-17	18/113
KEGG Pathway	hsa04540	Gap junction	1.568E-13	14/88
KEGG Pathway	hsa04912	GnRH signaling pathway	2.9678E-13	14/92
KEGG Pathway	hsa04270	Vascular smooth muscle contraction	1.3904E-11	14/121
KEGG Pathway	hsa04921	Oxytocin signaling pathway	3.0612E-10	14/152
KEGG Pathway	hsa05034	Alcoholism	2.566E-08	13/180
KEGG Pathway	hsa04730	Long-term depression	1.1787E-07	8/60
KEGG Pathway	hsa04720	Long-term potentiation	2.8339E-07	8/67
KEGG Pathway	hsa04713	Circadian entrainment	4.523E-06	8/96
KEGG Pathway	hsa04916	Melanogenesis	6.612E-06	8/101
KEGG Pathway	hsa04724	Glutamatergic synapse	0.00519522	5/114
KEGG Pathway	hsa04064	NF-kappa B signaling pathway	1.0754E-15	16/95
KEGG Pathway	hsa04670	Leukocyte transendothelial migration	1.127E-15	17/114
KEGG Pathway	hsa04360	Axon guidance	1.2273E-13	18/175
KEGG Pathway	hsa05100	Bacterial invasion of epithelial cells	7.5995E-07	8/76
KEGG Pathway	hsa05130	Pathogenic Escherichia coli infection	1.0288E-06	7/55

KEGG Pathway	hsa05219	Bladder cancer	3.3375E-15	12/41
KEGG Pathway	hsa05216	Thyroid cancer	3.1326E-07	6/29
KEGG Pathway	hsa04320	Dorso-ventral axis formation	6.8366E-06	5/28
KEGG Pathway	hsa05020	Prion diseases	0.00036073	4/35
KEGG Pathway	hsa05202	Transcriptional misregulation in cancer	1.5525E-14	19/180
KEGG Pathway	hsa04650	Natural killer cell mediated cytotoxicity	2.5182E-13	16/133
KEGG Pathway	hsa05146	Amoebiasis	3.404E-08	10/96
KEGG Pathway	hsa04960	Aldosterone-regulated sodium reabsorption	1.4366E-06	6/37
KEGG Pathway	hsa05010	Alzheimer's disease	1.0499E-12	17/171
KEGG Pathway	hsa04520	Adherens junction	4.9791E-12	12/72
KEGG Pathway	hsa04750	Inflammatory mediator regulation of TRP channels	1.1348E-11	13/97
KEGG Pathway	hsa04723	Retrograde endocannabinoid signaling	6.4314E-07	9/101
KEGG Pathway	hsa04310	Wnt signaling pathway	1.4473E-06	10/143
KEGG Pathway	hsa04972	Pancreatic secretion	4.523E-06	8/96
KEGG Pathway	hsa04971	Gastric acid secretion	8.5324E-06	7/75
KEGG Pathway	hsa04970	Salivary secretion	2.8388E-05	7/90
KEGG Pathway	hsa04961	Endocrine and other factor-regulated calcium reabsorption	9.1804E-05	5/47
KEGG Pathway	hsa04925	Aldosterone synthesis and secretion	0.00015029	6/82
KEGG Pathway	hsa04911	Insulin secretion	0.00018322	6/85
KEGG Pathway	hsa05032	Morphine addiction	0.00026606	6/91
KEGG Pathway	hsa04215	Apoptosis - multiple species	2.1076E-11	9/33
KEGG Pathway	hsa05014	Amyotrophic lateral sclerosis (ALS)	5.6495E-11	10/51
KEGG Pathway	hsa05134	Legionellosis	5.8484E-08	8/55
KEGG Pathway	hsa05416	Viral myocarditis	1.0301E-07	8/59
KEGG Pathway	hsa05016	Huntington's disease	0.00290332	7/193
KEGG Pathway	hsa04728	Dopaminergic synapse	3.717E-11	14/130
KEGG Pathway	hsa05031	Amphetamine addiction	3.185E-07	8/68
KEGG Pathway	hsa05030	Cocaine addiction	4.5761E-07	7/49
KEGG Pathway	hsa00380	Tryptophan metabolism	0.00688909	3/40
KEGG Pathway	hsa00590	Arachidonic acid metabolism	4.3298E-10	10/62
