# Supplementary material for: Network Pharmacology Reveals That Resveratrol Can Alleviate COVID-19-Related Hyperinflammation
Source: Dis Markers. 2021 Sep 22;2021:4129993. doi: 10.1155/2021/4129993 (PMC8463930; doi:10.1155/2021/4129993)
Supplement: Supplementary 6 — Supplementary Table S6: SARS-CoV-2 DEGs KEGG-enriched terms. [file 4129993.f6.pdf]

SARS-CoV-2 DEGs KEGG enriched terms

Category	Term	Description	P.value	InTerm_InList
KEGG Pathway	hsa04060	Cytokine-cytokine receptor interaction	2.61742E-19	35/270
KEGG Pathway	hsa04657	IL-17 signaling pathway	9.59366E-15	19/93
KEGG Pathway	hsa04668	TNF signaling pathway	1.69723E-13	19/108
KEGG Pathway	hsa05323	Rheumatoid arthritis	1.19606E-11	16/90
KEGG Pathway	hsa04621	NOD-like receptor signaling pathway	6.07599E-10	19/170
KEGG Pathway	hsa05134	Legionellosis	5.45103E-09	11/55
KEGG Pathway	hsa05162	Measles	5.30173E-09	16/134
KEGG Pathway	hsa05164	Influenza A	2.00359E-07	16/173
KEGG Pathway	hsa05144	Malaria	2.95966E-07	9/49
KEGG Pathway	hsa05133	Pertussis	1.63392E-06	10/76
KEGG Pathway	hsa04064	NF-kappa B signaling pathway	1.8066E-06	11/95
KEGG Pathway	hsa05140	Leishmaniasis	9.37339E-06	9/73
KEGG Pathway	hsa05146	Amoebiasis	1.38036E-05	10/96
KEGG Pathway	hsa05321	Inflammatory bowel disease (IBD)	2.98386E-05	8/65
KEGG Pathway	hsa05132	Salmonella infection	3.57713E-05	9/86
KEGG Pathway	hsa05152	Tuberculosis	3.80298E-05	13/179
KEGG Pathway	hsa04630	Jak-STAT signaling pathway	4.29555E-05	12/156
KEGG Pathway	hsa05168	Herpes simplex infection	5.35116E-05	13/185
KEGG Pathway	hsa05418	Fluid shear stress and atherosclerosis	8.35101E-05	11/142
KEGG Pathway	hsa04640	Hematopoietic cell lineage	9.26294E-05	9/97
KEGG Pathway	hsa04933	AGE-RAGE signaling pathway in diabetic complications	0.000108539	9/99
KEGG Pathway	hsa04610	Complement and coagulation cascades	0.000122549	8/79
KEGG Pathway	hsa04620	Toll-like receptor signaling pathway	0.000158585	9/104
KEGG Pathway	hsa05202	Transcriptional misregulation in cancer	0.000169209	12/180
KEGG Pathway	hsa04380	Osteoclast differentiation	0.000184672	10/130
KEGG Pathway	hsa05161	Hepatitis B	0.000421378	10/144
KEGG Pathway	hsa05143	African trypanosomiasis	0.000477149	5/35
KEGG Pathway	hsa04015	Rap1 signaling pathway	0.000685656	12/210
KEGG Pathway	hsa04062	Chemokine signaling pathway	0.000715913	11/182
KEGG Pathway	hsa05160	Hepatitis C	0.000873872	9/131

KEGG Pathway	hsa05142	Chagas disease (American trypanosomiasis)	0.003232988	7/102
KEGG Pathway	hsa05150	Staphylococcus aureus infection	0.004062958	5/56
KEGG Pathway	hsa04931	Insulin resistance	0.004214676	7/107
KEGG Pathway	hsa04670	Leukocyte transendothelial migration	0.005948355	7/114
KEGG Pathway	hsa05332	Graft-versus-host disease	0.00731079	4/41
KEGG Pathway	hsa05219	Bladder cancer	0.00731079	4/41
KEGG Pathway	hsa04664	Fc epsilon RI signaling pathway	0.009230879	5/68
KEGG Pathway	hsa04010	MAPK signaling pathway	0.00950931	11/255
KEGG Pathway	hsa05166	HTLV-I infection	0.009773601	11/256
